# Nature of GaO_*x*_ Shells Grown on Silica by Atomic Layer Deposition

**DOI:** 10.1021/acs.chemmater.3c00923

**Published:** 2023-08-28

**Authors:** Zixuan Chen, Nora K. Zimmerli, Muhammad Zubair, Alexander V. Yakimov, Snædís Björgvinsdóttir, Nicholas Alaniva, Elena Willinger, Alexander B. Barnes, Nicholas M. Bedford, Christophe Copéret, Pierre Florian, Paula M. Abdala, Alexey Fedorov, Christoph R. Müller

**Affiliations:** † Laboratory of Energy Science and Engineering, ETH Zürich, 8092 Zürich, Switzerland; ‡ School of Chemical Engineering, The University of New South Wales, Sydney, NSW 2052, Australia; § Department of Chemistry and Applied Biosciences, ETH Zürich, 8093 Zürich, Switzerland; ∥ CNRS, CEMHTI UPR3079, Université d’Orléans, F-45071 Orléans, France

## Abstract

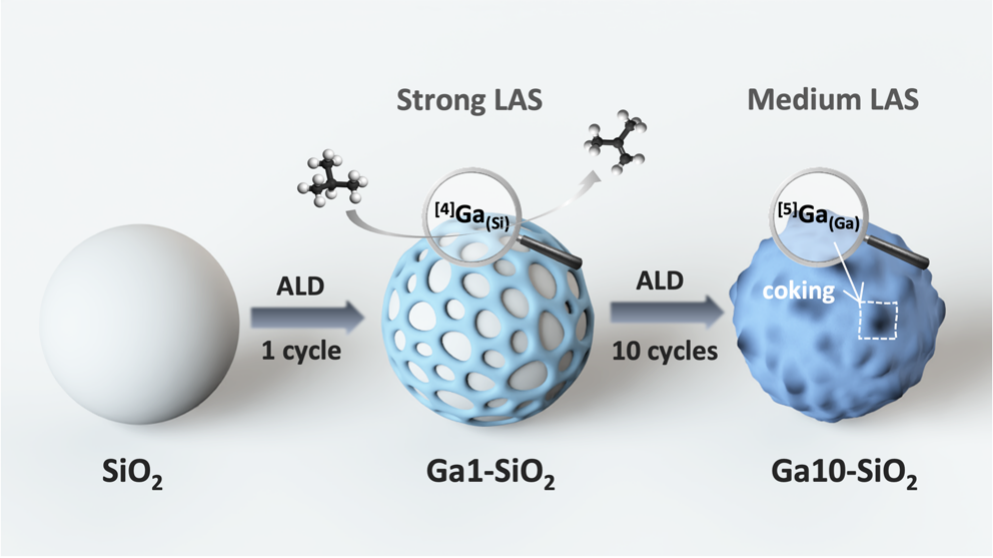

Gallia-based shells with a thickness varying from a submonolayer to ca. 2.5 nm were prepared by atomic layer deposition (ALD) using trimethylgallium, ozone, and partially dehydroxylated silica, followed by calcination at 500 °C. Insight into the atomic-scale structure of these shells was obtained by high-field ^71^Ga solid-state nuclear magnetic resonance (NMR) experiments and the modeling of X-ray differential pair distribution function data, complemented by Ga K-edge X-ray absorption spectroscopy and ^29^Si dynamic nuclear polarization surface enhanced NMR spectroscopy (DNP SENS) studies. When applying one ALD cycle, the grown submonolayer contains mostly tetracoordinate Ga sites with Si atoms in the second coordination sphere (^[4]^Ga_(Si)_) and, according to ^15^N DNP SENS using pyridine as the probe molecule, both strong Lewis acid sites (LAS) and strong Brønsted acid sites (BAS), consistent with the formation of gallosilicate Ga–O–Si and Ga–μ^2^-OH–Si species. The shells obtained using five and ten ALD cycles display characteristics of amorphous gallia (GaO_*x*_), i.e., an increased relative fraction of pentacoordinate sites (^[5]^Ga_(Ga)_), the presence of mild LAS, and a decreased relative abundance of strong BAS. The prepared Ga1-, Ga5-, and Ga10-SiO_2–500_ materials catalyze the dehydrogenation of isobutane to isobutene, and their catalytic performance correlates with the relative abundance and strength of LAS and BAS, viz., Ga1-SiO_2–500_, a material with a higher relative fraction of strong LAS, is more active and stable compared to Ga5- and Ga10-SiO_2–500_. In contrast, related ALD-derived Al1-, Al5-, and Al10-SiO_2–500_ materials do not catalyze the dehydrogenation of isobutane and this correlates with the lack of strong LAS in these materials that instead feature abundant strong BAS formed via the atomic-scale mixing of Al sites with silica, leading to Al–μ^2^-OH–Si sites. Our results suggest that ^[4]^Ga_(Si)_ sites provide strong Lewis acidity and drive the dehydrogenation activity, while the appearance of ^[5]^Ga_(Ga)_ sites with mild Lewis activity is associated with catalyst deactivation through coking. Overall, the atomic-level insights into the structure of the GaO_*x*_-based materials prepared in this work provide a guide to design active Ga-based catalysts by a rational tailoring of Lewis and Brønsted acidity (nature, strength, and abundance).

## Introduction

Materials based on gallium oxide (gallia) are used in various applications, e.g., as semiconductors, in optoelectronic devices, as well as heterogeneous catalysts, for instance, for the dehydrogenation of alkanes.^1−6^ In the context of heterogeneous catalysis, polymorphs of Ga_2_O_3_ (α-, β-, γ-, δ-, and ε-), in which surface Ga^3+^ sites reside in different coordination environments,^7−10^ have been studied with the aim to correlate specific surface properties (such as the Lewis acidity strength) with their catalytic performance in propane dehydrogenation (PDH).^11,12^ In particular, recent studies using unsupported α-, β-, and γ-Ga_2_O_3_ polymorphs have associated weak Lewis acidity (among these gallia polymorphs, β-Ga_2_O_3_ has the highest density of weak Lewis acid sites (LAS)) with high activity in PDH, while medium Lewis acidity in these unsupported gallia catalysts was related to deactivation by coking.^13,14^ In sharp contrast to unsupported Ga_2_O_3_-based PDH catalysts, the selective and stable active sites in silica-supported Ga-based catalysts are associated with strong LAS, viz., tetrahedral Ga sites as in the catalyst [(≡SiO)_3_Ga(XOSi≡)] (X is H or ≡Si).^15^ In general, industrial PDH catalysts rely on supports (Al_2_O_3_, SiO_2_, etc.) to yield a high dispersion of the active phase, and this often leads to a particle–support interaction and generation of interfacial sites.^16,17^ Therefore, understanding the role of and controlling the coordination geometry and acidity (Lewis and Brønsted) of surface and interfacial sites are necessary for the rational optimization of Ga-based dehydrogenation catalysts.^3,18^

Concerning the atomic-scale engineering of surface sites, atomic layer deposition (ALD) is a proven synthetic methodology that allows for a precise control over the growth process of a phase to be deposited onto a support.^19,20^ This control is enabled by the self-terminating nature of surface reactions between the gas-phase precursor and the surface functional groups (typically, hydroxyls); the following treatment (usually, with steam or ozone)^21,22^ allows the formation of new reactive surface functionalities and thereby enables the stepwise growth of tailored supported metal oxides upon repeating the process (reaction with molecular precursors—treatment to generate reactive surface sites).^23^ For instance, we have recently reported the atomic-scale structure of alumina layers obtained by ALD of trimethylaluminum (TMA) onto a partially dehydroxylated silica.^24^ With an increasing number of ALD cycles, the surface evolves from an aluminosilicate layer to a shell of amorphous alumina on top of a silica core (with an aluminosilicate interface). This structural variation leads to a decreasing abundance of strong Brønsted acid sites (BAS) with increasing thickness of the deposited layer. Such model AlO_*x*_/SiO_2_ supports allowed us to identify the role of strong BAS in the aromatization of ethylene and its oligomers for Ni-based ethene-to-propene catalysts.^25^

While reports on ALD-derived gallia films have utilized mostly flat substrates (Si-wafers, fused silica, or polyimide supports),^26−30^ studies on the deposition of GaO_*x*_ on powder substrates are limited.^31,32^ For instance, it has been demonstrated that the use of trimethylgallium (TMG) in combination with ozone is efficient for the growth of amorphous gallia films onto Si(100) or fused SiO_2_. Similarly, the deposition of gallium oxide onto mesoporous silica using TMG and steam leads to an amorphous GaO_*x*_ layer.^31^ However, our understanding of the atomic-scale structure of Ga sites in such ALD-derived GaO_*x*_ overcoatings, the dependence of the local Ga structure on the thickness of the overcoatings (i.e., the interface with silica or outer layers of thicker coatings), and the relationship between the local Ga structure/environment and surface acidity has to be improved to advance the rational design of catalytic materials. That being said, structural information about Ga sites may be gained by relying on dehydroxylated surfaces of supports used in the ALD process because dehydroxylated surfaces facilitate covalent attachment (grafting) of the ALD precursors onto the support (in preference to the reaction with the physisorbed water).^33^ Such an approach can enhance atomic-scale mixing at the interface between the deposited shell and the support.^24^

Here, we utilize ALD of TMG onto amorphous silica after dehydroxylation at 500 °C, in combination with ozone treatment, to prepare three Ga-SiO_2–500_ materials. Depending on the number of ALD cycles (1, 5, or 10 cycles, materials denoted as Ga1-, Ga5-, and Ga10-SiO_2–500_, respectively), the thickness of the grown GaO_*x*_ shells varies between a submonolayer in Ga1-SiO_2–500_ to ca. 1.5 and 2.5 nm in Ga5- and Ga10-SiO_2–500_. Fitting of high-field ^71^Ga magic-angle spinning solid-state nuclear magnetic resonance (MAS NMR) data obtained at 20.0 T and 28.2 T shows that all three prepared Ga-SiO_2–500_ materials contain mostly tetracoordinate Ga sites (^[4]^Ga), in addition to pentacoordinate Ga sites (^[5]^Ga), and only a minor amount of hexacoordinate Ga sites if present (ca. <5% ^[6]^Ga). The most probable local environment of ^[4]^Ga sites in Ga1-SiO_2–500_ is ^[4]^Ga(OGa)_1.2_(OSi)_2.8_ and it evolves to ^[4]^Ga(OGa)_2.5_(OSi)_1.5_ and ^[4]^Ga(OGa)_3.3_(OSi)_0.7_ in Ga5-SiO_2–500_ and Ga10-SiO_2–500_, respectively. In contrast to ^[4]^Ga sites, the most probable local environment of ^[5]^Ga sites does not change notably with the number of ALD cycles, suggesting that ^[5]^Ga sites interact less with the silica surface. The relative distributions of ^[4]^Ga, ^[5]^Ga and ^[6]^Ga sites obtained from NMR fittings are consistent with the results of Ga K-edge X-ray absorption spectroscopy (XAS) and differential pair distribution function (dPDF) analysis. Surface acidity studies using pyridine (Py) as a probe molecule for Fourier transform infrared spectroscopy (Py-FTIR) and ^15^N dynamic nuclear polarization surface enhanced NMR spectroscopy (DNP SENS) reveal the presence of a high relative abundance of strong BAS (i.e., protonated Py) mostly on Ga1-SiO_2–500_, owing to the presence of gallosilicate Ga–μ^2^-OH–Si species in this material. Applying further ALD cycles leads to the growth of an amorphous GaO_*x*_ phase on top of the gallosilicate layer, such that the relative abundance of strong BAS in Ga5- and Ga10-SiO_2–500_ decreases notably. The Ga-SiO_2–500_ materials prepared are active catalysts for isobutane dehydrogenation (BDH). Strong LAS, present on all three materials but most abundant in Ga1-SiO_2–500_, likely drive the BDH activity of the prepared Ga-based catalysts, while mild LAS increase coking. Our results highlight that ALD can be used to develop supported Ga-based dehydrogenation catalysts with a controllable relative abundance and strength of Lewis and Brønsted acidic sites and suggest that ^[4]^Ga_(Si)_ sites drive BDH activity while ^[5]^Ga_(Ga)_ sites increase coking.

## Experimental Section

### Materials

Silica agglomerates were prepared by wetting silica powder (AEROSIL 300, 99.9%, Evonik Industries) with deionized water to form a homogenous slurry followed by slow evaporation at 120 °C for 3 days. The silica agglomerates were then sieved to collect the fraction of particles in the size range 180–300 μm. The sieved silica support was heated to 500 °C (5 °C min^–1^), held overnight in static air, and then dehydroxylated at 500 °C (10^–5^ mbar, 20 h). The resulting material, denoted SiO_2–500_, has a surface area of 335 m^2^ g^–1^ and a pore volume of 2.0 mL g^–1^.

ALD of TMG (Pegasus Chemicals) onto SiO_2–500_ using ozone as an oxidant was performed on a PICOSUN R-200 system enclosed within an MBRAUN glovebox (O_2_, H_2_O < 1 ppm) at 300 °C using a varying number of ALD cycles (1, 5, or 10). The maximum loading of the sieved SiO_2–500_ power was limited to 300 mg to ensure the uniformity of the ALD coating. Each ALD cycle includes 20 pulses of TMG (0.1 s duration for each pulse) and 20 ozone pulses (5 s duration for each pulse). The number of TMG pulses was optimized to achieve a nearly complete consumption of the IR band due to isolated silanols (Figure S3). According to specifications, the ozone concentration expected is 10% w/w (140 g Nm^–3^). Each TMG or ozone pulse was followed by a 15 s N_2_ (99.999%) purge. The TMG source was kept at ambient temperature. The as-prepared materials after 1, 5, or 10 ALD cycles are denoted as TMG1-SiO_2–500_, TMG5-SiO_2–500_, and TMG10-SiO_2–500_, respectively. These materials were subsequently calcined in synthetic air at 500 °C for 4 h (5 °C min^–1^) and gave the three materials denoted as Ga1-SiO_2–500_, Ga5-SiO_2–500_, and Ga10-SiO_2–500_. FTIR spectra obtained from various batches of the prepared materials, either as-deposited or after calcination, were consistent with each other. In addition, comparable inductively coupled plasma optical emission spectroscopy (ICP-OES) results were obtained from various batches, showing a Ga wt % difference of less than 5% relative to the deposited Ga amount, providing overall robust evidence for the high reproducibility of the developed ALD protocol. All materials were handled and characterized without exposure to air, using air-tight flow reactors and glovebox techniques, except for the N_2_ physisorption, ICP-OES, electron microscopy, and X-ray powder diffraction (XRD) measurements.

### Methods

FTIR was performed on an Alpha II spectrometer (Bruker) operated inside an MBRAUN glovebox (O_2_, H_2_O < 1 ppm). The Ga content in the materials was determined using ICP-OES experiments. The surface area and pore volume of the materials were determined by N_2_ adsorption/desorption (Quantachrome NOVA 4000e) using the Brunauer–Emmett–Teller (BET) and Barrett–Joyner–Halenda (BJH) models, respectively. Transmission electron microscopy (TEM), scanning transmission electron microscopy (STEM) with a high-angle annular dark-field (HAADF) detection, and energy-dispersive X-ray spectroscopy (EDX) were carried out on an FEI Talos F200X transmission electron microscope. A JEOL JEM-ARM300F GRAND ARM scanning transmission electron microscope that was operated at 300 kV was also used. This microscope is equipped with a dual EDS system (two large area SDD EDX detectors with 100 mm^2^ active area; total solid angle: 1.6 sr). In order to avoid beam damage effects, all the shown annular dark-field scanning transmission electron microscopy (ADF-STEM) micrographs have been recorded under low-dose conditions.

XAS measurements were carried out at the BM31 station of the Swiss-Norwegian beamline (SNBL) at the European Synchrotron Radiation Facility (ESRF), Grenoble, France. All spectra were collected at the Ga K-edge using continuous scanning in transmission mode with a double-crystal Si(111) monochromator. To avoid contact with air, all sample pellets were sealed in two aluminized plastic bags using an impulse sealer inside a glovebox. The outside sealing layer was removed just before the measurement, while the inner sealing remained unmodified. Data treatment and analysis were carried out with Demeter software. The spectral energy was calibrated using a Zn foil (9658.6 eV). The extended X-ray absorption fine structure (EXAFS) spectra were fitted in R-space between 1 and 3 Å with a k-weight of 3. Fourier transformations were performed using a *k*-space window between 3 and 12 Å^–1^. The continuous Cauchy wavelet transform (CCWT) analysis was performed with MATLAB software using an open script downloaded from http://www.univ-mlv.fr/~farges/waw.^34^ The Cauchy order was set to 20 while analyzing the long-range EXAFS data in R space from 0.5 to 4 Å. To yield a better resolution of the CCWT images, we increased the Cauchy order to 150 for short-range R-space (2–4 Å) analysis.

XRD data were obtained using a Panalytical Empyrean diffractometer with a Cu Kα radiation source set at 45 kV and 40 mA. The detector used was an ultrafast line X’Celerator Scientific with Bragg–Brentano HD optics. The diffractograms were acquired between 5 and 70° (2θ; the step size was 0.0167° using 0.4 s step^–1^ acquisition time). In situ XRD was performed in the same instrument using an Anton Paar XRK 900 reactor chamber, in the range of 5–70° from room temperature to 900 °C (10 °C min^–1^) under synthetic air (50 mL min^–1^).

X-ray total scattering data were acquired on a laboratory goniometer-based X-ray scattering instrument (Empyrean by Malvern Panalytical) equipped with a Ag X-ray tube (λ = 0.56 Å for AgK_α_) and a hybrid pixel detector (GaliPIX) with a CdTe sensor. In a typical experiment, ca. 20 mg of the sample (Ga1-SiO_2–500_, Ga5-SiO_2–500_, Ga10-SiO_2–500_, and pristine SiO_2–500_ support) was placed between two quartz wool plugs in a 1 mm quartz capillary (0.01 mm wall thickness), which was subsequently sealed with wax inside an MBRAUN glovebox (O_2_, H_2_O < 1 ppm). Data from an empty capillary were obtained and subtracted from the scattering signal of the samples. The data were acquired in the 2θ range of 5–145° with a step size of 0.07° resulting in an instrumental *Q*_max_ = 21.4 Å^–1^, where *Q* = 4πsin(θ)/λ is the magnitude of the scattering vector. The total acquisition time was 22 h. Due to the relatively low GaO_*x*_ content in Ga1-SiO_2–500_, six measurements (22 h each) were averaged to increase the signal-to-noise ratio for this material. The pair distribution functions (PDFs or G(*r*)) were calculated from the total scattering data using PDFgetX3 V. 2.2.1^35^ over a Q-range from 1 to 18 Å^–1^, with *r*_poly_ = 1.0 and *r*_step_ = 0.03 Å, using the data from the empty capillary as the background (*r*_poly_ is the *r*-limit for the maximum frequency in the *F*(*Q*) correction polynomial, whereas *r*_step_ is the spacing of the grid on which *G*(*r*) is calculated). *F*(*Q*) is the reduced scattering function obtained from the X-ray total scattering data, and the details of the PDF data reduction have been described in the literature.^35^ For comparison and to calculate the differential PDF (dPDF), the intensities of the PDF were normalized by the maximum of the peak at ca. 1.5–1.6 Å corresponding to the first Si–O atomic pair. Subsequently, the PDF of pristine SiO_2–500_ was subtracted from the PDF of Ga1-, Ga5-, and Ga10-SiO_2–500_ to obtain the respective dPDF (Figure S27). A model-free dPDF analysis in the range 1–4 Å of the dPDF was performed using the SrMise software tool,^36^ which allows us to deconvolute the peaks and thus determine their positions and intensities assuming Gaussian peak functions in an automated manner. For the peak extraction, manually defined baselines were used and the data was sampled at the Nyquist frequency *Q*_max_/π (with *Q*_max_ = 18 Å^–1^) to achieve minimal correlation between the data points.^37^

Reverse Monte Carlo (RMC) simulations were performed on the dPDFs of Ga1-, Ga5-, and Ga10-SiO_2–500_ using the program fullrmc by applying nonperiodic boundary conditions with a random move generator for the Ga, O, and Si atom groups.^38^ The initial three-dimensional structures were constructed by placing β-Ga_2_O_3_ as an outer layer of spherical SiO_2_.^39,40^ The simulation sizes were selected based on the crystallite sizes observed under TEM. To construct the models for Ga1-, Ga5-, and Ga10-SiO_2–500_, SiO_2_ nanoparticles of diameter 6 nm were modified by placing on top layers of β-Ga_2_O_3_ with a thickness of 5 Å, 2 nm, and 4 nm, respectively. The RMC models were refined by introducing oxygen point defects through the atom removal generator in fullrmc,^41,42^ as the RMC fits obtained before applying the O atom removal generator were not satisfactory (Figure S30). Furthermore, the RMC simulation was guided by constraints such as intramolecular pair distances and average coordination numbers (CNs) (Table S5). To calculate the dPDFs from the modeled structures, the contributing weightage of the Si–Si and Si–O pairs was set to zero. Furthermore, the RMC simulated structures were analyzed by defining the bond distance as a proxy for the coordination environment. Average bond distances and CNs of relevant atomic pairs were calculated from the RMC-generated structures.

^71^Ga solid-state magic-angle spinning nuclear magnetic resonance (MAS NMR) experiments on Ga1-, Ga5-, and Ga10-SiO_2–500_ were performed on a Bruker NEO 20.0 T spectrometer operating at a frequency of 259.3 MHz. Materials were packed in an argon-filled glovebox inside 1.3 mm diameter zirconia rotors and were spun at 64 kHz under pure nitrogen. In order to increase the signal-to-noise ratio, the signal acquisition has been performed with a Carr–Purcell–Meiboom–Gill (CPMG) pulse sequence,^43^ coadding 128 echoes separated by 125 μs (i.e., eight rotor periods) and using radio-frequency fields set to 150 kHz with an optimized T_90_ pulse duration of 0.6 μs and a recycle delay of 0.5 s. These irradiation conditions did not produce any significant pulse bandwidth issues, as seen with the complementary short pulse echo (T_90_-τ-T_90_) and the variable-offset cumulative spectrum (VOCS) acquisition schemes (Figure S12).^44^ That being said, we did use the VOCS procedure (from 1.0 MHz to −800 kHz with a step of 100 kHz) for Ga5-SiO_2–500_ and Ga10-SiO_2–500_, but only CPMG was used for Ga1-SiO_2–500_ due to the low signal-to-noise ratio for this material. Total experimental times were 4.5, 8.5, and 12 h for Ga10-SiO_2–500_, Ga5-SiO_2–500_, and Ga1-SiO_2–500_, respectively.

Additional CPMG experiments were performed using the Ga10-SiO_2–500_ material and a Bruker AVANCE NEO spectrometer, operating at a Larmor frequency of 1200.956 MHz for ^1^H and 366.250 MHz for ^71^Ga (28.2 T). Here, Ga10-SiO_2–500_ was packed in a 1.3 mm outer diameter zirconia rotor inside a glovebox and closed with vespel caps. A modified 1.3 mm probe was used, spinning at 64 kHz with dry N_2_ gas, with echo delays of 312 μs (20 rotor periods) and coadding 128 echoes. The temperature of the probe head was maintained at 290 K using a Bruker VTU unit. The T_90_ pulse length was 15.0 μs (i.e., ν_rf_ ca. 30 kHz). The VOCS procedure was used, spanning a frequency range from −275 to 275 kHz by a step of 18.3 kHz (ca. 50 ppm), i.e., coadding 31 spectra, each with a recycle delay of 0.5 s. Note that the small pulse bandwidth did produce small line shape distortions in this case (Figure S13). Chemical shifts were referenced to a 1 M solution of Ga(NO_3_)_3_ in H_2_O (for both fields).

Simulations were performed using Dmfit software, in particular, the Czjzek model (or the Gaussian isotropic model, GIM) was used under the assumption of finite spinning speed, i.e., taking into account the spinning sidebands of the central transition arising from the second-order quadrupolar effects.^45,46^

For ^29^Si DNP SENS measurements, Ga1-, Ga5-, and Ga10-SiO_2–500_ materials were impregnated in a glovebox (O_2_ and H_2_O < 0.5 ppm) with 16 mM TEKPol in 1,1,2,2-tetrachloroethane (TCE) solution.^47,48^ The impregnated materials were introduced into a sapphire rotor (outer diameter 3.2 mm) and closed with a zirconia cap. The rotor was placed quickly in a cold DNP probe (109 K). The measurements were performed on a Bruker 600 MHz (14.1 T) instrument equipped with a 3.2 mm Bruker DNP double-resonance probe coupled to a 395 GHz gyrotron microwave source (output power = 6–10 W) to drive the DNP cross-effect. The static magnetic field was externally referenced by setting the ^1^H NMR signal of TCE to 6.9 ppm. In all experiments, a MAS rate of 8 kHz was used. A signal ^1^H solvent enhancement in the range of 29–33 was observed for all samples. ^1^H-^29^Si cross polarization (CP) pulse sequence with a contact time of 3 ms was used for ^29^Si NMR measurements. The DNP buildup time (τ_DNP_) was measured by a ^1^H saturation-recovery experiment with the microwaves turned on.

Direct excitation ^29^Si NMR experiments were performed on a Bruker Avance II 400 MHz spectrometer using 4 mm triple-resonance probe at 10 kHz MAS. The measurement was performed using a high-power decoupling (HPDEC) pulse sequence with a recycle delay of 313 s (measured by ^29^Si saturation-recovery experiment). The chemical shifts were indirectly referenced by setting most deshielded peaks of adamantane to 38.5 ppm.

Surface acidity of Ga-SiO_2–500_ materials was determined using pyridine as a probe molecule. ^15^N-pyridine (99% isotopic enrichment) was purchased from CortecNet Corp., dried over CaH_2_ at 60 °C for 2 days, and degassed by three freeze-pump-thaw cycles prior to use. The calcined materials were evacuated (10^–5^ mbar) and then exposed to the vapor pressure of ^15^N-Py at ambient temperature followed by outgassing (RT or 150 °C, 5 °C min^–1^, 10^–5^ mbar). The FTIR spectra were collected on an ALPHA II spectrometer (Bruker) operated inside a N_2_ glovebox using self-supporting pellets. For the ^15^N DNP SENS measurements, a similar procedure was followed as for the ^29^Si DNP SENS experiments described above, with the exception that a triple-resonance 3.2 mm Bruker DNP probe was used. The DNP-enhanced ^15^N NMR spectra were obtained using a ^1^H-^15^N CPMAS pulse sequence, employing a contact time of 2 ms.

### Catalytic Tests

BDH was tested in a benchtop Microactivity EFFI reactor (PID Eng&Tech). The prepared catalysts (50 mg) diluted with SiC (1.2 g, 46 grit, Alfa Aesar) were placed between two plugs of quartz wool on a quartz frit in a 13 mm ID quartz reactor. The reactor was sealed inside a N_2_ glovebox and purged with N_2_ via a bypass prior to the catalytic test. After reaching the desired reaction temperature (500 °C/550 °C, 10 °C min^–1^) under a flow of N_2_ (20 mL min^–1^), 10% of C_4_H_10_ in N_2_ (30 mL min^–1^, ambient pressure) was introduced into the reactor. The gas flow rate was controlled by mass flow controllers, which were calibrated to the desired gases. The temperature was controlled by a thermocouple immersed inside a catalyst bed. The products at the outlet of the reactor were analyzed by a four-channel compact gas chromatograph (CompactGC 4.0, Global Analyser Solutions) equipped with two thermal conductivity detectors (TCD) and two flame ionization detectors (FID). The data points were collected every 7 min. Catalyst regeneration was performed after 20 h of the BDH reaction by flowing synthetic air (50 mL min^–1^) for 1 h and flushing with N_2_ (50 mL min^–1^) for 10 min.

The fractions of gas-phase products (*C*_*x*, out_) were determined by calibrating the GC with a gas mixture of known composition (Carbagas). The formation rate of products (*F*_*x*, out_, mol min^–1^) was calculated based on the gas fractions in the outlet flow. The isobutane conversion (*X*_C_4_H_10__), the selectivity to gas-phase products (*S_x_*), and the carbon balance were calculated according to following equations (*N_x_* is the number of carbon atoms in the respective gas-phase product):
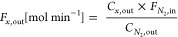

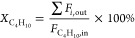

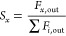

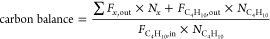


## Results and Discussion

### Material Synthesis

**Scheme 1:**
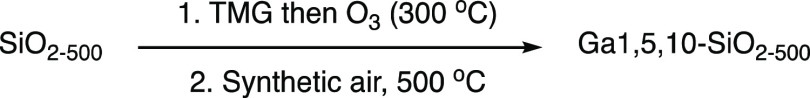
Synthesis of Ga1-, Ga5-, and Ga10-SiO_2–500_ Materials (1, 5, or 10 ALD Cycles, Respectively) Using Partially Dehydroxylated Silica and Alternating Pulses of TMG and Ozone

TMG1-, TMG5-, and TMG10-SiO_2–500_ were prepared using 1, 5, or 10 ALD cycles of TMG (300 °C deposition temperature). Amorphous silica was used as a support; it was first partially dehydroxylated at 500 °C (denoted SiO_2–500_), and ozone was used as the oxidant in the ALD cycle (Scheme 1).^28^ We chose ozone in preference to steam to minimize the rehydroxylation of the silica surface. Note that the interaction of TMG with the dehydroxylated silica in solution was reported to yield well-defined dimeric Ga species, formed via grafting of TMG onto silanol groups as well as opening siloxane bridges of silica.^49,50^ Calcination of the as-deposited TMG1-, TMG5-, and TMG10-SiO_2–500_ materials in synthetic air at 500 °C gave Ga1-, Ga5-, and Ga10-SiO_2–500_, respectively.

### N_2_ Physisorption and Elemental Analysis

ICP-OES measurements show that the weight loadings of Ga in Ga1-, Ga5-, and Ga10-SiO_2–500_ are 4.9, 18.2, and 31.9%, respectively, that is, the Ga content in the material increases with the number of applied ALD cycles. In addition, the BET specific surface area (*S*_BET_) of Ga1-, Ga5-, and Ga10-SiO_2–500_ is, respectively, 293, 202, and 160 m^2^ g^–1^ (Figure S1 and Table S1). BJH analysis of the N_2_ physisorption data shows that all of the three Ga-SiO_2–500_ materials contain only the intergranular porosity of the SiO_2_ support, i.e., there is no additional porosity in the coating layer. The pore volume as well as the pore size decrease with increasing thickness of the GaO_*x*_ shells (Figure S2 and Table S1).

### FTIR Spectroscopy

**Figure 1:**
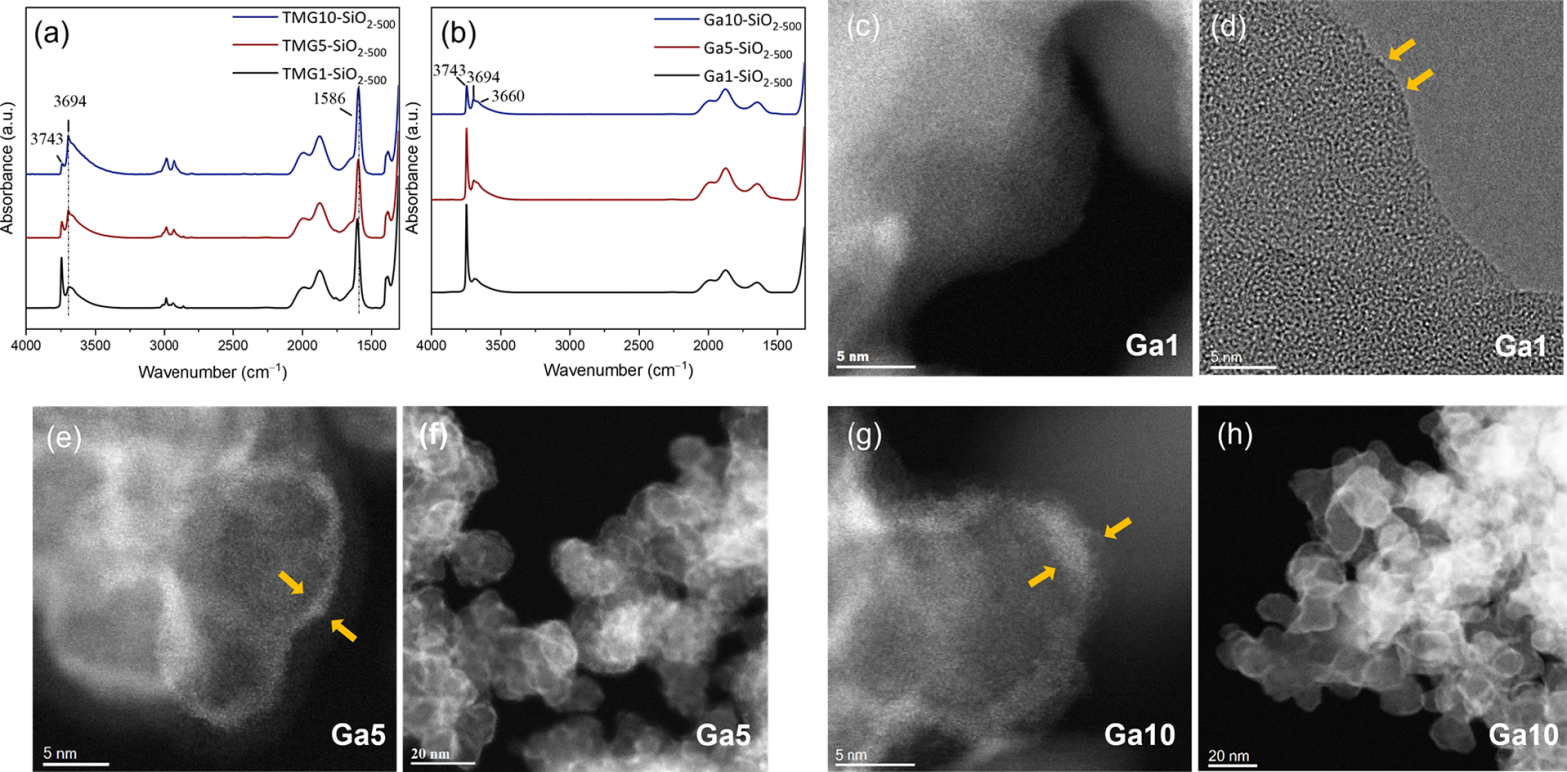
Transmission FTIR spectra of (a) TMG1-, TMG5-, and TMG10-SiO_2–500_ (black, dark red, and blue, respectively) and (b) Ga1-, Ga5-, and Ga10-SiO_2–500_ (black, dark red, and blue, respectively). ADF-STEM and HRTEM images of Ga1-SiO_2–500_ (c and d, respectively) and ADF-STEM images of Ga5-SiO_2–500_ (e,f) and Ga10-SiO_2–500_ (g,h). The yellow arrows indicate the ALD-grown film.

FTIR spectra of the as-deposited materials TMG1-, TMG5-, and TMG10-SiO_2–500_ show that the intensity of the IR band due to isolated silanols at ca. 3743 cm^–1^ decreases continuously with the increasing number of ALD cycles, whereas the intensity of an emerging broad band centered at ca. 3694 cm^–1^ increases, suggesting an interaction between the deposited Ga species and adjacent OH groups (Figures 1a and S4).^49,51^ The IR band at 1586 cm^–1^ is present in all three TMG-SiO_2–500_ materials and it is likely due to carbonate species formed by the interaction between the surface and the CO_2_ released during ozonolysis (Figure 1a).^52,53^ However, formate species can be intermediates on the pathway for oxidation of methyl groups to carbonates, and the presence of a low intensity band at 2856 cm^–1^ in all three TMG-SiO_2–500_ suggests the coexistence of minor amounts of formates.^28,54^ In addition, IR bands at 2830–3040 cm^–1^ (ν_CH_ stretching modes) and 1430–1350 cm^–1^ (δ_CH_ bending modes) indicate that the oxidation of the deposited TMG by ozone at 300 °C is incomplete (Figure 1a).^24^ It should be noted that while a complete oxidation of methyl groups in the grafted TMG species through ozonolysis has been reported previously, this study utilized a higher ozonolysis temperature (350 °C).^28^ However, an additional calcination treatment of the three TMG-SiO_2–500_ materials at 500 °C in synthetic air removes all bands associated with residual alkyl groups and surface carbonates (Figure 1b). The IR band due to isolated silanols is partially regenerated after the calcination and the intensity of this band decreases with the number of ALD cycles in Ga1-, Ga5-, and Ga10-SiO_2–500_. The above-described results are qualitatively similar to those observed for the deposition of TMA onto SiO_2–500_.^24^ Interestingly, after calcination of the TMG-SiO_2–500_ materials, the band at 3694 cm^–1^ partially remains and becomes narrower. The band at 3694 cm^–1^ is more pronounced in Ga10-SiO_2–500_ relative to Ga5- and Ga1-SiO_2–500_ (Figure S4). The position of this band is very close to that of GaOH in γ/β-Ga_2_O_3_ nanoparticles (3690–3693 cm^–1^).^13,14^ An additional broad band centered at 3660 cm^–1^ is observed in Ga1-, Ga5-, and Ga10-SiO_2–500_. It has been reported that β-Ga_2_O_3_ also features a band at ca. 3660 cm^–1^ and thus it may be attributed to GaOH.^13,14^ An alternative explanation for this broad band is its attribution to silanol groups interacting with Lewis acidic Ga^3+^ surface sites.^55,56^

### Transmission Electron Microscopy and X-ray Powder Diffraction

ADF-STEM, high-resolution TEM (HRTEM), EDX imaging, and EDX line mapping were performed to further investigate the coatings deposited onto SiO_2–500_. While the shell grown on Ga1-SiO_2–500_ is too thin to be visualized clearly in the STEM mode (Figure 1c), HRTEM images are consistent with the formation of a submonolayer Ga coating in Ga1-SiO_2–500_ (Figures 1d and S5). A core–shell morphology can be clearly visualized in the ADF-STEM images of Ga5-SiO_2–500_ and Ga10-SiO_2–500_ (Figure 1e,f and g,h, respectively; yellow arrows in Figure 1e,g indicate the ALD-derived coatings). The thicknesses of the shells are ca. 1.5 nm in Ga5-SiO_2–500_ and ca. 2.5 nm in Ga10-SiO_2–500_. Complementary EDX line mappings of Ga10-SiO_2–500_ confirm a core–shell microstructure, i.e., the majority of the Ga signal is located in the shell and the intensity of the Si signal in the shell is weaker (Figure S8). The presence of a core–shell microstructure is further verified by EDX mapping of Ga10-SiO_2–500_ (Figure S7). We note that while Ga1-SiO_2–500_ appeared to be stable during HRTEM measurements, the shells in Ga5-SiO_2–500_ and Ga10-SiO_2–500_ were prone to electron beam damage, i.e., the amorphous Ga-rich coatings evolved during the measurement into crystalline planes, which are found to coexist with regions of an amorphous Ga-rich shell (Figure S6). Inferences from microscopy results are consistent with the analysis of XRD data that show the absence of crystalline phases in all three prepared Ga-SiO_2–500_ materials, viz., only XRD amorphous halos due to diffuse scattering from silica and gallia are detected (Figure S9).

### ^71^Ga Solid-State NMR

**Figure 2:**
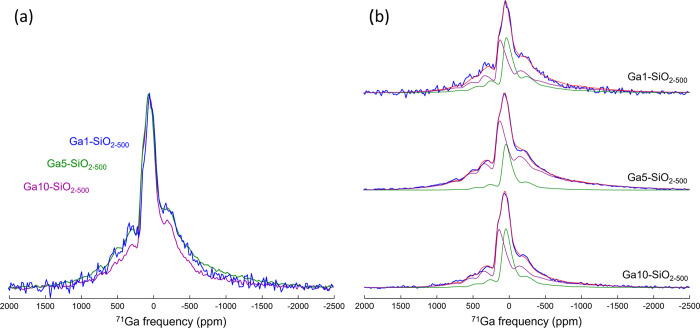
Experimental ^71^Ga MAS NMR CPMG spectra (20.0 T, spinning rate 64 kHz). (a) Ga1-, Ga5-, and Ga10-SiO_2–500_ shown in blue, green, and purple, respectively, after normalization to their maxima. (b) Top, middle, and bottom in blue are the spectra of Ga1-, Ga5-, and Ga10-SiO_2–500_, respectively. A two-site simulation is presented in red on the right side, with the individual fitted components shown in purple (^[4]^Ga) and green (^[5]^Ga).

A quantitative analysis of the local coordination of Ga in our ALD-derived materials was performed using ^71^Ga MAS NMR. Note that the ^71^Ga isotope (natural abundance is 39.6%) features large quadrupolar interactions resulting in a significant line broadening of the ^71^Ga NMR signal, which lowers the detection sensitivity even with fast magic-angle spinning.^57^Figure 2 presents the ^71^Ga NMR spectra of Ga1-, Ga5-, and Ga10-SiO_2–500_ collected at 20.0 T, along with the two-site simulations of these spectra. Only minor differences in the line shapes are noted between the spectra of Ga1-, Ga5-, and Ga10-SiO_2–500_. In fact, these spectra are dominated by a set of intense spinning sidebands arising from the strong second-order quadrupolar broadening of the central transition and, due to the structural disorder, display a distribution of this interaction. While the spinning sidebands can be well separated in a quadrupolar phase-adjusted spinning sideband (QPASS) experiment (Figure S14), the limitations with the pulse length prevent the recovery of an undistorted line shape when using the QPASS sequence. This result demonstrates the notable strength of the quadrupolar interaction experienced by the ^71^Ga nuclei, resulting in the observed poor resolution. It can be expected that when using a higher magnetic field, this effect is reduced, however, as will be discussed in more detail below; the spectrum of Ga10-SiO_2–500_ obtained at 28.2 T suggests that the distribution of quadrupolar couplings has a major impact on the linewidth, in addition to the chemical shift anisotropy (that is proportional to the magnetic field), which also plays a significant role in the intensity of the spinning sidebands such that a higher resolution is not obtained at 28.2 T when spinning at 64 kHz.

While the ^71^Ga spectra of Ga1-, Ga5-, and Ga10-SiO_2–500_ at 20.0 T presented in Figure 2a do not exhibit clearly resolved features, simulations using a GIM (Czjzek model) allow us to differentiate the individual components and retrieve relevant spectroscopic parameters, i.e., the mean isotropic chemical shift δ̅_iso_, the width of its Gaussian distribution Δδ_iso_, the variance of the distribution of the quadrupolar tensor elements (σ_Q_), and the relative population of each component. To determine if a two-component or three-component fitting of the ^71^Ga MAS NMR spectra is more appropriate, an additional NMR measurement was conducted at a higher magnetic field (28.2 T) for Ga10-SiO_2–500_, which has the highest Ga loading of the three materials (31.9 wt %). The simulation using two components is presented in Figure S15, and it accounts well for the line shape obtained at 20.0 T; however, it accounts only partially for the line shape at 28.2 T. That being said, the three-component simulation for Ga10-SiO_2–500_ presented in Figure S15 improves the simulation at 20.0 T but it does not lead to a significant improvement of the simulation at 28.2 T. The difficulty in obtaining a reasonable line shape fitting for the spectrum at 28.2 T arises from the line shape distortions (Figure S13); in addition, our model does not take into account the chemical shift anisotropy. Both two- and three-component simulations give two major components with the average isotropic chemical shift δ̅_iso_at ca. 190 ppm and 100 ppm (Table S2), assigned to ^[4]^Ga and ^[5]^Ga coordination environments, respectively.^58^ The three-site simulation for Ga10-SiO_2–500_ provides an additional component with δ̅_iso_ = 44 ppm, which is consistent with hexacoordinate ^[6]^Ga sites.^12,59^ However, it should be noted that the variance of the quadrupolar coupling distribution (σ_Q_) is significantly lower for this site than the ones found for ^[4]^Ga and ^[5]^Ga sites, indicating that the three-site simulation may be unreliable. Moreover, the relative fraction of the ^[6]^Ga site is small (4%) and its impact on the fitting of the spectrum at 28.2 T is negligible. Therefore, these results show the presence of structurally disordered ^[4]^Ga and ^[5]^Ga sites and suggest the lack of detectable amounts of ^[6]^Ga sites (ca. <5%). In what follows, we discuss the results of the two-component simulations presented in Table 1.

**Table 1:** ^71^Ga NMR Parameters Retrieved from the Two-Component Simulations: Relative Fraction, Average Isotropic Chemical Shift δ̅_iso_, and Full Width at Half Maximum of the Distribution of δ_iso_ (Δδ_iso_) and Variance of the Distribution of Quadrupolar Tensor Elements (σ_Q_)^a^

material	^[4]^Ga	δ̅_iso_	Δδ_iso_	σ_Q_	^[5]^Ga	δ̅_iso_	Δδ_iso_	σ_Q_	^[av]^Ga
	%	ppm	ppm	MHz	%	ppm	ppm	MHz	
Ga1-SiO_2–500_	59	176	48	12.7	41	94	72	10.5	4.41
Ga5-SiO_2–500_	81	187	61	13.7	19	97	70	8.6	4.19
Ga10-SiO_2–500_	63	194	49	11.9	37	103	79	8.8	4.37

a ^[av]^Ga denotes the average coordination number.

Three spectra obtained at 20.0 T were fitted with a two-component fit: one component with a δ̅_iso_ in the range of 176–194 ppm and a second component with a δ̅_iso_ in the range of 94–103 ppm (Figure 2b), ascribed to ^[4]^Ga and ^[5]^Ga sites, respectively.^59−61^ Comparison of the three fitted spectra reveals an increase of the chemical shifts for both components as the number of ALD cycles increases (Table 1). For instance, the δ̅_iso_ of ^[4]^Ga sites increases from 176 ppm in Ga1-SiO_2–500_ to 187 and 194 ppm in Ga5-SiO_2–500_ and Ga10-SiO_2–500_, respectively, consistent with an increased substitution of silicon by gallium in the second coordination sphere, that is, a gradual change from a Ga–O–Si to Ga–O–Ga local environment with increasing number of ALD cycles.^62^ More specifically, it has been reported that a substitution of one Al atom by one Si atom in the second coordination sphere of Al results in a −3 ppm shift in the ^27^Al NMR spectra,^63^ and this shift corresponds to a ca. −8.5 ppm shift for a substitution of one Ga atom by one Si atom in the second coordination sphere of Ga.^58^ Since the isotropic chemical shift of ^27^Al nuclei is not highly sensitive to the presence of OH groups (i.e., aluminols),^64^ we assume that δ̅_iso_ of ^71^Ga is relatively insensitive to the presence of OH groups as well and therefore assign the variations of δ̅_iso_ to changes of the Si/Ga ratio in the second coordination sphere. Considering that the ^[4]^Ga site in β-Ga_2_O_3_ is found at 200 ppm, the δ̅_iso_ of 176 ppm found for Ga1-SiO_2–500_ translates to ^[4]^Ga(OGa)_1.2_(OSi)_2.8_ being the most probable local environment in Ga1-SiO_2–500_. On the other hand, the δ̅_iso_ = 187 ppm found for Ga5-SiO_2–500_ is consistent with a ^[4]^Ga(OGa)_2.5_(OSi)_1.5_ local environment and the δ̅_iso_ = 194 ppm determined for Ga10-SiO_2–500_ relates to a ^[4]^Ga(OGa)_3.3_(OSi)_0.7_ environment. Similar differences in δ_iso_ have been reported for Ga sites inside a silicious zeolite framework (168.0 and 171.9 ppm, ^[4]^Ga(OSi)_4.0_ environments) and outside of the framework (176.2 ppm, ^[4]^Ga(OSi)_<4.0_ environments).^62^ Interestingly, in contrast with ^[4]^Ga sites, ^[5]^Ga sites are found to not evolve significantly with the number of ALD cycles, suggesting that the ^[5]^Ga sites do not interact strongly with the silica surface. We also observe that the average Ga CN, ^[av]^Ga, increases from 4.19 in Ga5-SiO_2–500_ to 4.37 in Ga10-SiO_2–500_. Owing to the relatively low Ga loading in Ga1-SiO_2–500_ (4.9 wt %), the signal-to-noise ratio in the spectrum of this material was notably lower than in Ga5- and Ga10-SiO_2–500_ and for this reason (i.e., a higher experimental error), the average Ga CN for Ga1-SiO_2–500_ might be higher than that for Ga5- and Ga10-SiO_2–500_.

To summarize, simulations of the ^71^Ga NMR data of Ga1-, Ga5-, and Ga10-SiO_2–500_ suggest that disordered ^[4]^Ga sites in Ga1-SiO_2–500_ interact strongly with Si atoms, i.e., forming Ga–O–Si linkages. Subsequent ALD cycles provide a coating containing gallium sites in an amorphous GaO_*x*_ environment composed of distorted ^[4]^Ga and ^[5]^Ga sites (major and minor, respectively). Notably, the fraction of ^[6]^Ga sites does not exceed ca. 5% in all three prepared materials. This insight will be considered for the analysis of the dPDF and Ga K-edge XAS results as discussed below.

### Ga K-Edge X-ray Absorption Near Edge Structure

**Figure 3:**
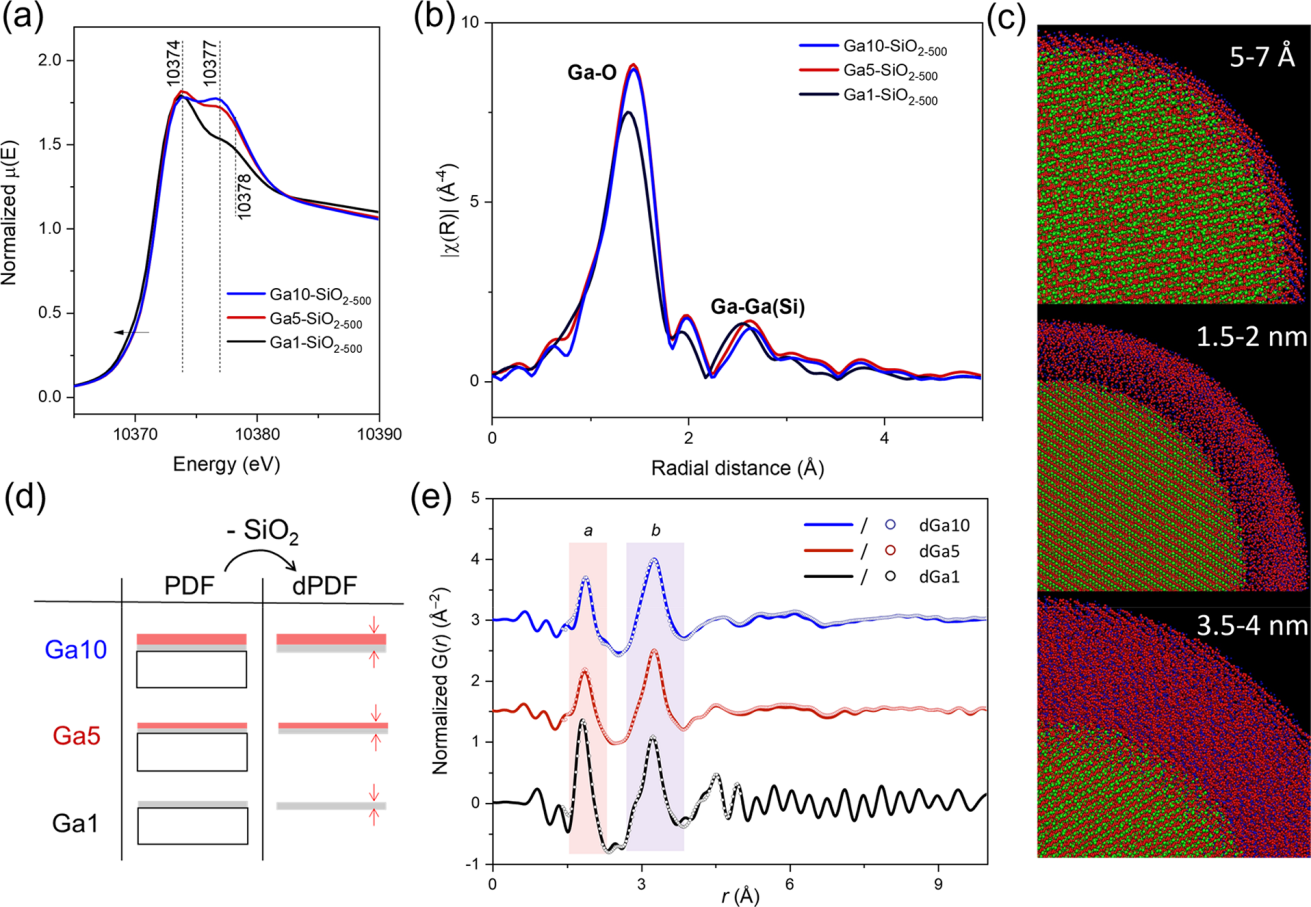
Ga K-edge (a) XANES spectra and (b) Fourier transformed (FT) EXAFS functions (nonphase-corrected) of Ga1-, Ga5-, and Ga10-SiO_2–500_ (black, red, and blue, respectively). (c) Structural models utilized for the PDF fittings. (d) Schematic of Ga1-, Ga5-, and Ga10-SiO_2–500_, viz., a bulk SiO_2_ support coated with a layer of GaO_*x*_, for PDF and dPDF analysis. (e) Experimental dPDF for Ga1-, Ga5-, and Ga10-SiO_2–500_ (black, red, and blue, respectively) and their corresponding RMC fitting results (empty circles).

X-ray absorption near edge structure (XANES) data at the Ga K-edge provided additional element-specific information on the Ga coordination in the prepared Ga-SiO_2–500_ materials.^65^ Ga1-SiO_2–500_ displays a main feature in the white line region at ca. 10374 eV and a second, notably less intense feature at ca. 10378 eV (Figure 3a). These two features are also present in Ga5-SiO_2–500_ and Ga10-SiO_2–500_. While the relative intensity between the two features (i.e., at 10374 and 10377 eV) is comparable in Ga10-SiO_2–500_ and Ga5-SiO_2–500_ (slightly lower relative intensity of the 10377 eV feature is observed in Ga5-SiO_2–500_), the relative intensity of the feature at 10377 eV is significantly lower in Ga1-SiO_2–500_. We note that the white line features at 10374 and 10377 eV are typically ascribed to ^[^^4]^Ga and ^[^^6]^Ga sites, respectively, yet these assignments relate to Ga sites in zeolites (^[4]^Ga sites, Figure S16)^66,67^ and crystalline Ga_2_O_3_ phases (^[4]^Ga and ^[^^6]^Ga sites).^68,69^ According to the fittings of the high field ^71^Ga MAS NMR results discussed above, all three Ga-SiO_2–500_ materials lack significant amounts (i.e., beyond ca. 5%) of ^[6]^Ga sites and therefore we attribute the white line feature at 10377–10378 eV to ^[5]^Ga sites. The higher energy (10378 eV) and the lower intensity of the ^[5]^Ga feature in Ga1-SiO_2–500_ relative to Ga5- and Ga10-SiO_2–500_ (10377 eV) indicates a lower abundance of ^[^^5]^Ga sites, possibly due to a higher abundance of ^[4]^Ga–O–Si linkages relative to ^[5]^Ga–O–Ga linkages in this material. Interestingly, Ga1-SiO_2–500_ has a slightly lower absorption edge energy relative to Ga5- and Ga10-SiO_2–500_ (10371.7, 10371.9, and 10372.0 eV, respectively) explained by the on average more abundant interaction with the silica support and the formation of a gallosilicate surface layer in Ga1-SiO_2–500_ (Figure 3a). We fitted the XANES white line peaks using Gaussian and arctangent functions to evaluate the relative distribution of ^[4]^Ga and ^[^^5]^Ga coordination environments in the series of Ga-SiO_2–500_ materials. The fitting results show a gradual decrease in the relative abundance of ^[4]^Ga sites, i.e., from ca. 75% in Ga1-SiO_2–500_ to 62% in Ga5-SiO_2–500_ and 57% in Ga10-SiO_2–500_ (Table S3 and Figure S17), in general agreement with the trend observed by ^71^Ga MAS NMR.

### Ga K-Edge EXAFS

The FT EXAFS functions of Ga1-, Ga5-, and Ga10-SiO_2–500_ are shown in Figure 3b, and the fitting results are summarized in Table 2. All three materials feature predominantly a first coordination shell, corresponding to a Ga–O scattering path (Figures S18–S20). The fitted Ga–O bond distance increases with the growing thickness of the GaO_*x*_ layer, i.e., Ga1-SiO_2–500_ yields a Ga–O distance of 1.83(1) Å whereas Ga5-SiO_2–500_ and Ga10-SiO_2–500_ feature a longer Ga–O distance of 1.86(3) and 1.87(3) Å, respectively. The average CN for the first shell is 4.4(2) in Ga1-SiO_2–500_ and 4.6(3) in Ga5- and Ga10-SiO_2–500_. This observation indicates that ^[4]^Ga sites, with a shorter Ga–O distance than ^[5]^Ga sites, are most abundant in Ga1-SiO_2–500_, as also indicated by XANES.^70^ With the growth of the GaO_*x*_ layer, ^[5]^Ga sites become more abundant, which leads to an increase in the Ga–O distances and CNs. The second sphere, dominated by the Ga–Ga scattering paths (and with some additional contribution from the Ga–Si paths), has a low intensity in all of the three Ga-SiO_2–500_ materials when compared with crystalline Ga_2_O_3_,^13^ consistent with an amorphous nature of the ALD-grown layers.^70^ The fitted Ga–Ga distance increases slightly from 2.80(2) Å for Ga1-SiO_2–500_ (Table S4) to 2.90(1) Å in both Ga5- and Ga10-SiO_2–500_ (Table 2). Worthy of note, including a Ga–Si path to the fitting of the EXAFS spectra of Ga1-SiO_2–500_ allows for a decrease in the *R*-factor (i.e., the agreement between the experimental and the calculated data) from 0.05 to 0.03 relative to the fittings that consider only Ga–Ga paths (Figure S21 and Tables 2 and S4). This result points to the presence of Si atoms (i.e., Ga–O–Si linkages) in the second coordination sphere of Ga atoms in Ga1-SiO_2–500_.

**Table 2:** Best Fits of the Structural Parameters Obtained from Ga K-Edge EXAFS FT^a^

material	neighbor	CN	*r* (Å)	σ^2^ (Å^2^)	*E*_0_ (eV)	*R*-factor
Ga1-SiO_2–500_	O	4.4(2)	1.83(1)	0.007(1)	6(1)	0.003
	Si	0.2(2)	2.60(3)	0.0076^b^		
	Ga	0.5(1)	2.80(2)	0.0076^b^		
Ga5-SiO_2–500_	O	4.6(3)	1.86(3)	0.008(4)	7.4(9)	0.004
	Ga	0.8(5)	2.90(1)	0.008(1)		
Ga10-SiO_2–500_	O	4.6(3)	1.87(3)	0.008(1)	7.4(9)	0.004
	Ga	0.8(6)	2.90(1)	0.008(1)		

a All samples were measured at ambient temperature. *S*_0_^2^ was fixed to 1 as obtained by fitting a β-Ga_2_O_3_ reference. CN refers to the coordination number; *E*_0_ refers to edge energy uncertainty.

b σ^2^, the mean squared displacement of the half path length, was constrained to the same value for the Ga–Ga and Ga–Si paths.

To further probe for the formation of a gallosilicate phase in Ga1-, Ga5-, and Ga10-SiO_2–500_, we assessed the contribution of Ga–Ga and Ga–Si scattering paths in the second sphere by performing a CCWT analysis of the EXAFS data.^34^ Such analysis provides a correlation of *r* (interatomic distances) and the *k* (wave vector) spaces, thus allowing the distinction between two different scattering atoms positioned at similar distances from the Ga center. Atoms with higher atomic numbers scatter more strongly at higher *k* values than atoms with lower atomic numbers. The CCWT analysis of the EXAFS data of a reference-unsupported β-Ga_2_O_3_ shows two distinct features. One feature with a maximum intensity in the range of *r* = 1.2–1.4 Å and *k* = 4.8–5.6 Å^–1^ is ascribed to a Ga–O scattering path and the second feature in the range of *r* = 2.5–3.0 Å and *k* = 9.5–10.5 Å^–1^ is ascribed to a Ga–Ga path (Figure S25). CCWT analyses of Ga1-, Ga5-, and Ga10-SiO_2–500_ show a predominant feature due to a Ga–O scattering path and a rather weak second coordination sphere (Figures S22–S24). Performing the CCWT analysis in a more narrow R range around the second coordination sphere, i.e., 2–4 Å instead of 0.5–4 Å, allows us to exclude the first Ga–O coordination sphere and to focus on features due to the Ga–Ga and Ga–Si scattering paths in Ga1-, Ga5-, and Ga10-SiO_2–500_ (Figures S22–S24).^15^ All three materials show a main feature centered at *r* = 2.4–2.6 Å and *k* = 8.0–10.0 Å^–1^, assigned to Ga–Ga scattering paths. Ga1-SiO_2–500_ shows a clearly observable feature at *r* = 2.4–2.6 Å and *k* = 5.0–8.0 Å^–1^,^15^ assigned to a Ga–Si scattering path and in line with the formation of a gallosilicate interface in Ga1-SiO_2–500_. The Ga–Si scattering path in Ga5- and Ga10-SiO_2–500_ cannot be excluded by this analysis but it is clearly weaker than in Ga1-SiO_2–500_.

### dPDF Analysis

X-ray total scattering was used to gain further insight into the atomic-scale structure of our ALD-derived materials. Figure S27 shows the X-ray total scattering data for Ga1-, Ga5-, Ga10-SiO_2–500_, and SiO_2–500_. All patterns contain no Bragg peaks and display diffuse X-ray scattering, consistent with the absence of crystalline phases, as discussed above (Transmission Electron Microscopy and X-ray Powder Diffraction section). To investigate in more detail the local structure of the formed GaO_*x*_ layers, we turn to real space analysis of the X-ray total scattering data and perform a differential PDF analysis by subtracting the signal of SiO_2–500_, after normalization, from the signals of Ga1-, Ga5-, Ga10-SiO_2–500_ (Figures 3d,e and S26, see the Supporting Information (SI) for details). The subtraction of the signal of SiO_2–500_ allows us to isolate the contribution of the ALD layer to the scattering data. The dPDF traces contain, in the region between 1 and 4 Å, two prominent features, labeled *a* and *b* (Figures 3e and S28). The peak *a* is due to Ga–O atomic pairs,^10,13^ while the peak *b* contains contributions from Ga–Ga (dominant), Ga–O, O–O, and, as indicated by ^71^Ga MAS NMR, ^29^Si DNP SENS (vide infra), and EXAFS analysis, Ga–Si atomic pairs. There are no evident atomic pairs for *r* exceeding ca. 5 Å, in line with the lack of a long-range order in the prepared materials, indicating a characteristic local structure that extends up to ca. 5 Å. An initial assessment of the average interatomic distances was performed through a peak fitting analysis (model free) of the dPDF data (Figure S28), which reveals a lower Ga–O interatomic distance in Ga1-SiO_2–500_ relative to Ga5-SiO_2–500_ and Ga10-SiO_2–500_ (*r_a_* = 1.85(1), 1.88(1), and 1.88(1) Å, respectively), which is likely due to a higher relative fraction of ^[4]^Ga sites in Ga1-SiO_2–500_, as indicated by XANES and EXAFS analyses discussed above. Ga sites with lower CNs feature shorter Ga–O distances relative to higher coordinated Ga sites.^71−73^ The average Ga–O distances in our ALD-made materials are also shorter than that observed for β-Ga_2_O_3_ (comprising 50% ^[4]^Ga and 50% ^[6]^Ga, *r* = 1.885 Å), possibly due to the higher abundance of ^[4]^Ga sites, the presence of ^[5]^Ga sites in our materials, and the lack of significant amounts of ^[6]^Ga sites. Moreover, the intensity of the peak *b* relative to peak *a* increases in the order Ga1-SiO_2–500_ < Ga5-SiO_2–500_ ≈ Ga10-SiO_2–500_, which may be due to a lower CN of the Ga–Ga shell and/or due to a higher fraction of Ga–Si paths in Ga1-SiO_2–500_.^74^

Next, to describe quantitatively the local structure of the prepared materials (i.e., the statistical distribution of Ga sites, average CNs, and interatomic distances), the dPDF data were modeled with an RMC method.^41,75^ The RMC simulation was performed in a differential mode, i.e., neglecting the contribution from Si–O and Si–Si pairs (due to SiO_2_) in the dPDF profiles. In addition, constraints on Ga–O distances and CNs are used as specified in Table S5 and an O atom removal generator was used to assess the presence of any point defects in the ALD-grown GaO_*x*_ (see the SI for details).^41,42^ The resulting RMC fits are depicted in Figures 3e and S29, while the applied structural models of the materials are shown in Figure 3c. The quality of the RMC-calculated dPDF profiles is assessed by the total standard errors, which are 0.196, 0.064, and 0.185 for Ga1-, Ga5-, and Ga10-SiO_2–500_, respectively, indicating a good agreement between the simulations and the experimental data (Table 3). Subsequently, the RMC-generated structures were used to extract the structural parameters of the materials. These results demonstrate that the calculated CNs and bond lengths for the Ga–O pairs are in a reasonable agreement with the values obtained from the EXAFS fittings, although the CNs for the second coordination shell are larger than those determined by EXAFS (Tables 2 and 3) due to the greater sensitivity of the PDF to higher coordination shells as compared to EXAFS.

**Table 3:** Summary of the Structural Parameters Including the Average Interatomic Distances (*r*) and Average CNs Extracted from RMC Simulations

material	scattering pair	*r* (Å)	avg. CN	^[4]^Ga (%)	^[5]^Ga (%)	^[6]^Ga (%)	std. error
Ga1-SiO_2–500_	Ga–O	1.8(1)	4.3(5)	63	28	9	0.196
	Ga–Ga	3.2(1)	5.5(9)				
	Ga–Si	3.2(4)	1.2(4)				
Ga5-SiO_2–500_	Ga–O	1.9(1)	4.5(6)	60	26	14	0.064
	Ga–Ga	3.2(2)	5.7(9)				
	Ga–Si		<0.1				
Ga10-SiO_2–500_	Ga–O	1.9(1)	4.5(7)	60	25	15	0.185
	Ga–Ga	3.2(1)	6(1)				
	Ga–Si		<0.1				

Furthermore, we observed an increase in the Ga–Ga CNs in Ga5- and Ga10-SiO_2–500_ with respect to Ga1-SiO_2–500_, likely due to a lower relative fraction of Ga atoms at the surface with respect to Ga atoms within the GaO_*x*_ shell (considering that Ga at the surface is less coordinated than Ga in the bulk) and due to the presence of Ga–Si paths in Ga1-SiO_2–500_ (Table 3). Furthermore, the distribution of ^[4]^Ga, ^[5]^Ga, and ^[6]^Ga sites in the Ga–O path extracted from the RMC models shows an increase of ^[6]^Ga sites from 9% in Ga1-SiO_2–500_ to 14 and 15% in Ga5- and Ga10-SiO_2–500_, respectively, which is also linked with the increase in the Ga–Ga CNs. That being said, in all three materials the fraction of ^[6]^Ga sites is notably lower than that of the ^[4]^Ga and ^[5]^Ga sites and this is generally consistent with the obtained ^71^Ga NMR results and the lower average Ga–O distances when compared to β-Ga_2_O_3_, as determined by both dPDF and EXAFS analyses. Lastly, it is to be noted that the CNs for the Ga–Si pairs as obtained from RMC modeling are substantial only in Ga1-SiO_2–500_, with an average CN of ca. 1.2(4), while the contribution of Ga–Si neighbors in Ga5- and Ga10-SiO_2–500_ is negligible due to the dominant signal arising from the GaO_*x*_ shells.

### ^29^Si Dynamic Nuclear Polarization Nuclear Magnetic Resonance

**Figure 4:**
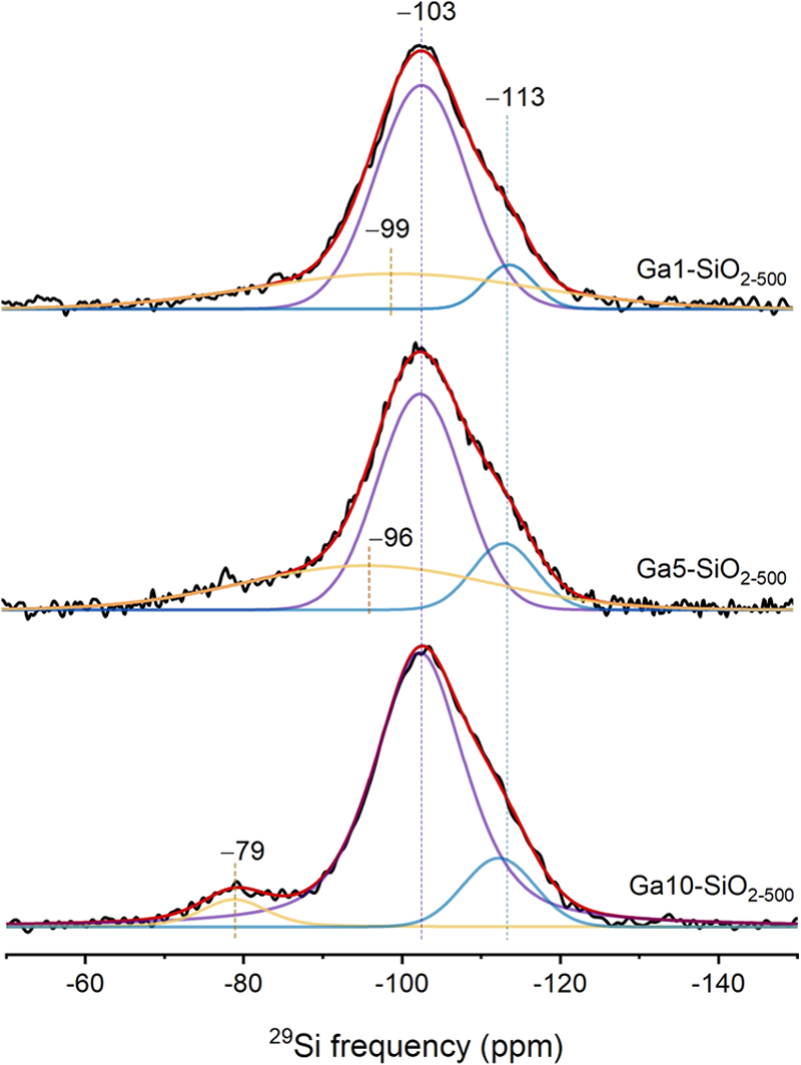
^29^Si DNP SENS experimental (black) and simulated (red) spectra of Ga1-, Ga5-, and Ga10-SiO_2–500_. All spectra were recorded at 14.1 T. Fitted components are shown in purple, blue, and yellow.

In order to gain further insight into the coordination geometry of Si atoms that have Ga atoms in the second coordination sphere (Si_(Ga)_ sites), we also acquired ^29^Si DNP SENS data. This surface-sensitive NMR method relies on the transfer of polarization, at a temperature of ca. 100 K, from the hyperpolarized electrons of an added organic radical to the surrounding protons and subsequently to the nuclei of interest, in this case, via ^1^H-^29^Si CP.^76^ The ^29^Si DNP SENS data of Ga1-, Ga5-, and Ga10-SiO_2–500_ and the respective fittings are presented in Figure 4. The spectrum of Ga1-SiO_2–500_ was fitted with three components (unconstrained fit) that entail chemical shifts of −99, −103, and −113 ppm. The two latter components correspond, respectively, to Q_3_ and Q_4_ silicon sites originating from surface silanols and near-surface layers of the silica core.^77,78^ The additional intensity at a higher chemical shift is fitted with a broad component centered at −99 ppm. We attribute this feature to the presence of a combination of Si_(4Ga)_, Si_(3Ga)_, Si_(2Ga)_, and Si_(1Ga)_ sites that display distinct chemical shifts.^39,79^ Interestingly, with increasing thickness of the GaO_*x*_ coating, the center of the broad component shifts to lower fields, viz., it is centered at −96 ppm Ga5-SiO_2–500_. This is explained by a higher relative fraction of Si_(3Ga)_ and Si_(2Ga)_ sites relative to Si_(1Ga)_ sites. In this context, in addition to Q_3_ and Q_4_ silicon sites, the experimental spectrum of Ga10-SiO_2–500_ can be fitted with a narrow component centered at −79 ppm, attributed to Si_(4Ga)_ sites.^56^ This observation indicates that Si atoms likely diffuse inside the growing GaO_*x*_ film.

Overall, analysis of the ^29^Si DNP SENS data shows that the coordination environment of Si at the interface with GaO_*x*_ in Ga1-SiO_2–500_ (ca. 4.9 wt % Ga loading) includes a distribution of Si_(Ga)_ sites spanning from one to four Ga atoms in the second coordination shell. With increasing thickness of the GaO_*x*_ layer in Ga5-SiO_2–500_ (ca. 18.2 wt % Ga loading) the distribution of sites shift to Si atoms with an increased number of Ga in the second coordination shell (such as Si_(3Ga)_ and Si_(2Ga)_ sites), converging to mostly to Si_(4Ga)_ in Ga10-SiO_2–500_ (ca. 31.9 wt % Ga loading). While these Si_(Ga)_ sites are observed by ^29^Si DNP SENS, their contribution to all Ga sites in Ga5-SiO_2–500_ and Ga10-SiO_2–500_ is low, due to the high loading of Ga in these materials, making their detection by Ga K-edge XAS and dPDF methods challenging, as was discussed above.

### Surface Acidity by Py-FTIR and ^15^N DNP SENS

The catalytic performance (activity, selectivity, and stability) of Ga-based catalysts in alkane dehydrogenation is linked to their surface acidity properties.^13^ Therefore, first we studied the acidity of the surface sites in Ga1-, Ga5-, and Ga10-SiO_2–500_ by FTIR using pyridine (Py) as the probe molecule.^80^

In Py-FTIR, the characteristic vibrational modes of pyridine coordinated to LAS appear at ca. 1630–1600 cm^–1^ (the higher wavenumbers are associated with stronger Lewis acidity) and at ca. 1450 cm^–1^.^81^ The pyridinium ion (PyH^+^), formed upon protonation of pyridine by strong BAS, gives IR bands at ca. 1640 and ca. 1545 cm^–1^.^82,83^ The region at ca. 1490 cm^–1^ contains overlapping bands of Py adsorbed on LAS (Py-L) and PyH^+^, while pyridine bound to weak BAS exhibits bands between ca. 1570 and 1590 cm^–1^ and at ca. 1440 cm^–1^.^13,84^

**Figure 5:**
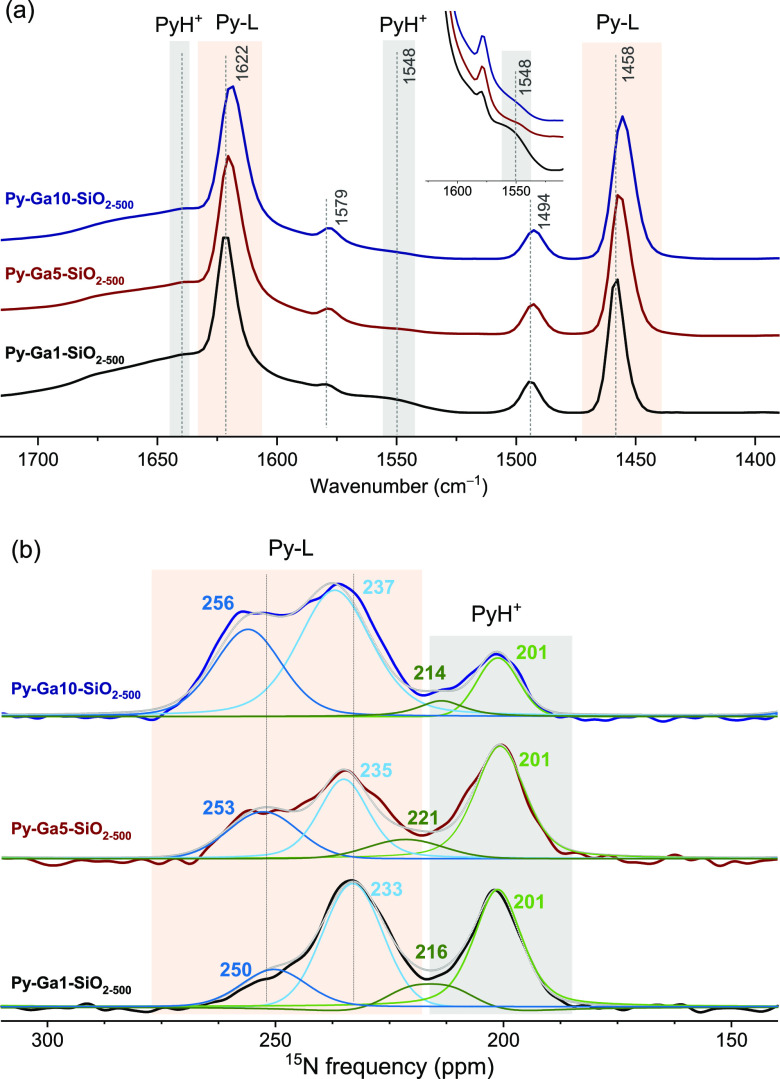
(a) FTIR spectra of Py-Ga1-SiO_2–500_ (black), Py-Ga5-SiO_2–500_ (red), and Py-Ga10-SiO_2–500_ (blue) after desorption of pyridine at 150 °C and (b) corresponding ^15^N DNP SENS spectra obtained in a 14.1 T magnetic field, 8 kHz MAS rate, and a temperature of 109 K. PyH^+^ and Py-L are highlighted in light gray and brown, respectively. L indicates a Lewis acid site. The number of components in the fit was limited to four, one for strong LAS, one for medium LAS, and two for strong BAS.

Figure 5a shows the FTIR spectra of pyridine that remains adsorbed on the surface of Ga1-, Ga5-, and Ga10-SiO_2–500_ after desorption at 150 °C. Characteristic features of Py bound to LAS at ca. 1619–1622, 1492–1494, and 1456–1458 cm^–1^ are observed in all three materials; however, their exact position and the bandwidth vary slightly with the thickness of the GaO_*x*_ overlayer. On Ga1-SiO_2–500_, adsorbed pyridine exhibits bands at ca. 1622, 1494, and 1458 cm^–1^, and these bands broaden and shift gradually to lower wavenumbers with an increasing number of ALD cycles (Figure 5a). This observation points to the presence of at least two types of LAS in Ga5- and Ga10-SiO_2–500_. Py-FTIR data indicate that with the growth of the GaO_*x*_ overlayer, Ga-SiO_2–500_ materials become less Lewis acidic. The inset in Figure 5a shows IR bands at ca. 1545 cm^–1^ corresponding to PyH^+^. This band is clearly visible in Ga1-SiO_2–500_ but is less intense in Ga5-SiO_2–500_ and in particular Ga10-SiO_2–500_.

Insights gained from the Py-FTIR data were further refined via ^15^N DNP SENS measurements.^85^ The ^15^N chemical shift of protonated pyridine (due to strong BAS) is found at ca. 200–220 ppm,^86−88^ while the ^15^N resonance of pyridine bonded to LAS appears between ca. 230 and 280 ppm, with peaks at a lower chemical shift corresponding to Py bound to stronger LAS. H-bonded pyridine on weak BAS is found between 290 and 260 ppm and physisorbed pyridine at 317 ppm.^83,89^

The ^15^N DNP SENS spectra of the Ga1-, Ga5-, and Ga10-SiO_2–500_ materials after the desorption of ^15^N-Py at 150 °C are presented in Figure 5b. Details of the fittings are provided in Table S6. Four resonances can be distinguished through the fitting of the experimental data, and the relative intensity of these peaks varies with the thickness of the GaO_*x*_ coating. The resonance centered at 201 ppm, intensive in Py-Ga1-SiO_2–500_ and Py-Ga5-SiO_2–500_ but notably weaker in Py-Ga10-SiO_2–500_, is assigned to PyH^+^ and indicates the presence of strong BAS. The presence of related strong BAS has been reported previously for silica-supported amorphous GaO_*x*_ nanoparticles prepared by the impregnation of gallium nitrate; however, the intensity of the corresponding peak in the nitrate-impregnated material was notably lower when compared to Ga1- and Ga5-SiO_2–500_.^13^ This implies that the ALD approach allows us to maximize the interaction between gallia and SiO_2_, generating thereby more abundant gallosilicate sites that are associated with strong BAS. Based on previous reports, also the broad resonance around 214–221 ppm can be assigned to PyH^+^,^24^ indicating therefore the presence of two distinct, strong BAS of varying acidity strength.

Turning now to adducts of Py with Lewis sites, while Py-Ga5-SiO_2–500_ and Py-Ga10-SiO_2–500_ show distinct peaks due to strong LAS (235 ppm and 237 ppm, respectively) and medium LAS (253 and 256 ppm, respectively), Py-Ga1-SiO_2–500_ features the signature due to medium strength LAS at 250 ppm only as a minor peak when compared to the peak at 233 ppm that is due to strong LAS. Pyridine bonded to weak LAS is not observed in any of our Ga-SiO_2–500_ materials (*T*_des_ = 150 °C). Interestingly, both peaks due to Py on strong and medium LAS undergo a continuous deshielding (i.e., weakening of the Lewis acidity) with the increasing thickness of the GaO_*x*_ coating.

Overall, both Py-FTIR and ^15^N DNP SENS results show that the nature of the surface acid sites evolves with the growth of the amorphous GaO_*x*_ layer. Abundant, strong Brønsted acidity, likely due to the presence of pseudobridging Ga–μ^2^-OH–Si sites, is found mostly in Ga1-SiO_2–500_ and, according to Py-FTIR, to a lesser extent in Ga5-SiO_2–500_. With the growth of the GaO_*x*_ layer, the relative fraction of BAS with respect to that of LAS decreases, reaching its lowest ratio in Ga10-SiO_2–500_. Furthermore, also the fraction of medium LAS increases with the growing thickness of the GaO_*x*_ layer.

### Catalytic Performance

**Figure 6:**
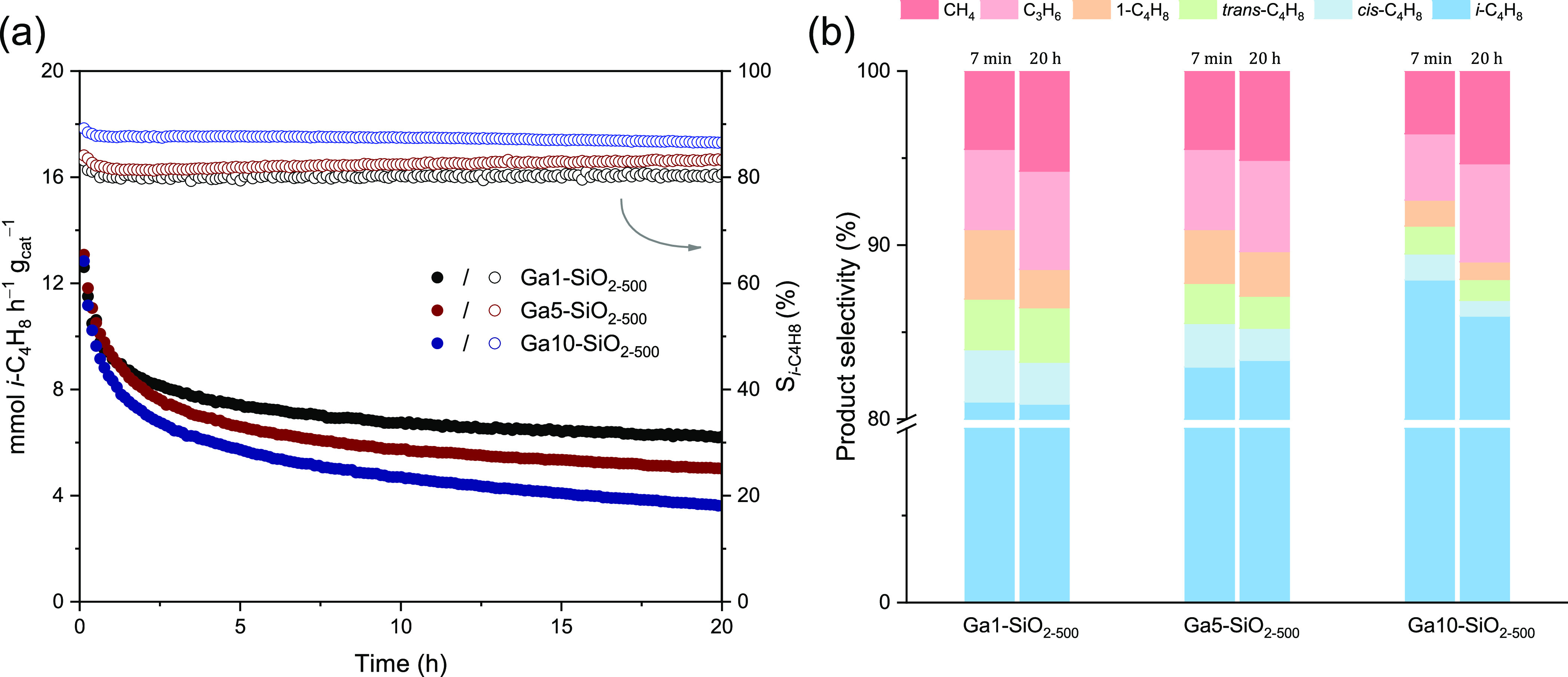
(a) STY of isobutene (mmol *i*-C_4_H_8_ h^–1^ g_cat_^–1^) over 20 h TOS and (b) partial product selectivity (i.e., selectivity among shown products) on Ga1-, Ga5-, and Ga10-SiO_2–500_ after TOS = 7 min and 20 h. Reaction condition: 10% of *i-*C_4_H_10_ in N_2_, WHSV = 8.5 h^–1^, *T* = 500 °C. See the SI for details.

Ga1-, Ga5-, and Ga10-SiO_2–500_ were tested in a model reaction, i.e., BDH, in a plug-flow reactor at 500 °C using 10% *i*-C_4_H_10_ in N_2_ (WHSV = 8.5 h^–1^). A summary of the catalytic results is presented in Table 4 and Figure 6 shows changes in the conversion of isobutane and the selectivity to isobutene during 20 h time-on-stream (TOS). While all three catalysts are active for BDH and show a similar initial space time yield (STY), expressed as mmol *i*-C_4_H_8_ h^–1^ g_cat_^–1^, their deactivation rates with TOS differ (Figure 6a and Table 4). Specifically, Ga1-SiO_2–500_ deactivates to a lesser extent over 20 h TOS in comparison to Ga5-SiO_2–500_ and Ga10-SiO_2–500_ (*k*_d_ = 0.037, 0.051, and 0.067 s^–1^, respectively, see Table 4 for the definition of *k*_d_). The STY of Ga1-, Ga5-, and Ga10-SiO_2–500_ at TOS = 7 min is 12.6, 13.1, and 12.8 mmol *i*-C_4_H_8_ h^–1^ g_cat_^–1^, respectively, and decrease to 6.1, 4.9, and 3.5 mmol *i*-C_4_H_8_ h^–1^ g_cat_^–1^ after 20 h TOS.

**Table 4:** Results of the N_2_ Physisorption Experiments (BET), ICP, and Catalytic Tests^a^

material	*S*_BET_ (m^2^ g^–1^)	Ga content (wt%)	isobutane conversion (%)	isobutene selectivity (%)	carbon balance (%)	*k*_d_ (s^–1^)	rate of isobutene production	
							mmol h^–1^ g_cat_^–1^	mmol h^–1^ mol_Ga_^–1^ m^–2^
Ga1-SiO_2–500_	293	4.9	9.8 (4.9)	81 (81)	100 (97)	0.037	13.2 (6.1)	63.7 (31.0)
Ga5-SiO_2–500_	202	18.2	10.2 (3.9)	83 (83)	100 (97)	0.051	13.1 (4.9)	24.8 (11.3)
Ga10-SiO_2–500_	160	31.9	9.6 (2.7)	88 (86)	100 (97)	0.067	12.8 (3.5)	15.7 (4.8)

a Catalytic data are presented after 7 min TOS and in parentheses, after 20 h TOS. Reaction condition: 10% of *i*-C_4_H_10_ in N_2_, WHSV = 8.5 h^–1^, T = 500 °C. *k*_d_ = [ln(1 – conv_end_/conv_end_) – ln(1 – conv_start_/conv_start_)]/*t*.^90^

It should be noted that the Ga content in the tested catalysts, as determined by ICP-OES, is notably different, i.e., it increases with the number of ALD cycles. Hence, to allow for a better comparison of different catalysts, we normalized the activity of Ga1-, Ga5-, and Ga10-SiO_2–500_ by the Ga content and the surface area (determined by BET analysis). The initial normalized activities of Ga1-SiO_2–500_, Ga5-SiO_2–500_, and Ga10-SiO_2–500_ are ca. 63.7, 24.8, and 15.7 mmol *i*-C_4_H_10_ h^–1^ mol_Ga_^–1^ m^–2^ (Table 4 and Figure S33), i.e., the activity of Ga1-SiO_2–500_ is significantly higher than that of Ga5-SiO_2–500_ and Ga10-SiO_2–500_. However, the normalized activity of the latter two catalysts can be underestimated since they contain a higher fraction of Ga sites in subsurface layers relative to Ga1-SiO_2–500_. At TOS = 20 h, the activity of Ga1-, Ga5-, and Ga10-SiO_2–500_ reaches 31.0, 11.3, and 4.8 mmol *i*-C_4_H_10_ h^–1^ mol_Ga_^–1^ m^–2^, respectively, while Ga10-SiO_2–500_ shows a slightly higher selectivity to isobutene compared to Ga5-SiO_2–500_ and Ga1-SiO_2–500_ (ca. 88, 83, and 81%, respectively, see Figure 6b and Table 4). Noteworthy, the selectivity to isobutene is stable with TOS, i.e., it decreased by merely 2% for Ga10-SiO_2–500_ after 20 h TOS and remained unchanged for Ga1- and Ga5-SiO_2–500_ during the entire catalytic test. A further discussion of the catalytic tests as well as results of regeneration studies are provided in the SI file.

**Scheme 2:**
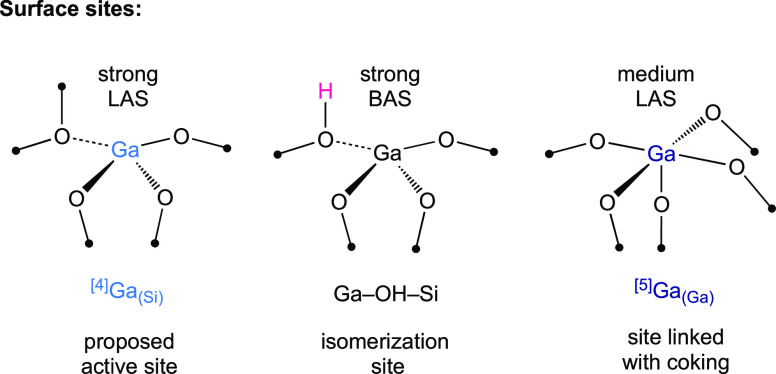
Schematic Illustration of Surface Sites in Ga1-, Ga5-, and Ga10-SiO_2–500_ Catalysts and Their Proposed Roles in the BDH Reaction

Overall, Ga1-SiO_2–500_, which features a larger relative fraction of strong LAS than Ga5- and Ga10-SiO_2–500_, exhibits higher catalytic activity and stability than Ga5- and Ga10-SiO_2–500_. Therefore, we conclude that strong LAS, attributed to tetracoordinate Ga^3+^ sites in our materials, are the most active sites for BDH,^15^ while mild LAS, attributed to pentacoordinate Ga^3+^ sites, contribute to coke formation and thus catalyst deactivation. More abundant strong BAS in Ga1-SiO_2–500_ relative to Ga5- and Ga10-SiO_2–500_ are likely responsible for the slightly lower isobutene selectivity of Ga1-SiO_2–500_ (Scheme 2).

It is also interesting to note that the materials Al1-, Al5-, and Al10-SiO_2–500_, prepared in a similar way by ALD of TMA onto dehydroxylated silica,^24^ exhibit barely any activity in BDH, with the isobutane conversion below 1% and isobutene selectivity at ca. 50%, which is similar to what is observed for the diluent SiC (Table S8). A likely explanation for such diverging alkane dehydrogenation activity trends between Al- and Ga-based materials is the lack of active sites in Al-based materials, i.e., the lack of strong tetracoordinate Al^3+^ LAS (^4^Al_(Si)_) owing to their transformation into Al–μ^2^-OH–Si sites with strong Brønsted acidity, i.e., pseudobridging silanols^91^ (Figure S43). In other words, a larger extent of atomic-scale mixing in Al1-SiO_2–500_ relative to Ga1-SiO_2–500_ yields more abundant Brønsted acidity in Al1-SiO_2–500_ but at the same time consumes strong LAS, which is the likely active site in alkane dehydrogenation reactions of the catalyst family considered in this work (note that peaks of Py bound to the strongest LAS in Al1-SiO_2–500_ and Ga1-SiO_2–500_ are found at 239 and 233 ppm, respectively).^24^

## Conclusions

We have utilized ALD of TMG (Me_3_Ga) onto partially dehydroxylated silica, followed by calcination, to prepare silica-supported GaO_*x*_ shells. The thickness of the grown shells varies from a submonolayer in Ga1-SiO_2–500_ up to a thickness of, respectively, ca. 1.5 and 2.5 nm in Ga5- and Ga10-SiO_2–500_. High field ^71^Ga solid-state NMR, Ga K-edge XAS, and dPDF supported by RMC modeling were applied to elucidate the atomic-scale structure of ALD-grown GaO_*x*_ films. Ga1-SiO_2–500_ features a submonolayer coating containing gallosilicate species with gallium in an environment that is composed mostly of tetracoordinate Ga sites, along with a lower amount of pentacoordinate Ga sites. The application of additional ALD cycles reduces the relative abundance of ^[4]^Ga sites, i.e., from ca. 81% in Ga5-SiO_2–500_ to ca. 63% in Ga10-SiO_2–500_, which is offset by an increasing amount of ^[5]^Ga sites. The evolution of the surface from a mostly gallosilicate to a predominantly amorphous GaO_*x*_ shell leads to a variation in the type and strength of surface acidity. Strong BAS, i.e., Ga–μ^2^-OH–Si sites, form due to the atomic-scale mixing of strong LAS (such as ^[4]^Ga_(2–3Si)_ sites) with the silanol groups of the silica support and therefore are found preferentially in materials that are prepared using a low number of ALD cycles. Such surfaces also feature strong LAS due to ^[4]^Ga_(Si)_ centers that are not engaged in the interaction with a silanol group and therefore retain their strong Lewis acidic properties. In contrast, the amount of mild LAS increases with a growing thickness of the GaO_*x*_ layer, and this correlates with an increased relative abundance of ^[5]^Ga sites. The relative strength of both strong and mild LAS decreases with a growing thickness of the GaO_*x*_ layer, explained by the gradual replacement of surface sites with a ^[4]^Ga_(Si)_ local environment by ^[4]^Ga_(Ga)_ sites (i.e., replacement of Si with a Ga environment in the second coordination sphere).

We also provided evidence that the type of surface acidities of the prepared Ga-SiO_2–500_ materials correlates with their catalytic activity in isobutane dehydrogenation. Ga1-SiO_2–500_, the catalyst that contains a larger relative amount of ^[4]^Ga centers as well as more abundant strong LAS, is found to be more active and stable in BDH, while a higher fraction of ^[5]^Ga centers (in Ga5 and Ga10-SiO_2–500_) correlates with a higher fraction of mild LAS, leading in turn to an increased degree of coking.^13^ Additionally, the comparison between ALD-made Al- and Ga-based surface silicates shows that the formation of strong Brønsted acidic sites is less pronounced for Ga (i.e., a lower extent of atomic-scale mixing than in the case of Al), rendering strong LAS more abundant for Ga-based materials, which correlates with the presence dehydrogenation activity for Ga-based silicates and the lack of dehydrogenation activity for Al-based silicates. Overall, our study reports an approach to control the relative fraction of strong and mild LAS, as well as strong BAS, in silica-supported, Ga-based alkane dehydrogenation catalysts.

## Supporting Information Available

Experimental procedures, additional data analyses, N_2_ adsorption and desorption isotherms, IR spectra, HRTEM and ADF-STEM images, XRD patterns, ^71^Ga ssNMR, PDF, XANES, EXAFS, Wavelet transform analysis of EXAFS data, RMC fittings, and catalytic results (PDF)
