# Supplementary material for: Nature of GaOx Shells Grown on Silica by Atomic Layer Deposition
Source: Chem Mater. 2023 Aug 28;35(18):7475–90. doi: 10.1021/acs.chemmater.3c00923 (PMC10536998; doi:10.1021/acs.chemmater.3c00923)
Supplement: Supplementary file 1 — cm3c00923_si_001.pdf [file cm3c00923_si_001.pdf]

Supporting Information

Nature of GaO_x Shells Grown on Silica by Atomic Layer Deposition

Zixuan Chen[1], Nora K. Zimmerli[1], Muhammad Zubair[2], Alexander V. Yakimov[3], Snædís Björgvinsdóttir[3], Nicholas Alaniva[3], Elena Willinger[1], Alexander B. Barnes[3], Nicholas M. Bedford[2], Christophe Copéret[3], Pierre Florian[4,*], Paula M. Abdala[1,*], Alexey Fedorov[1,*], Christoph R. Müller[1,*]

[1] Laboratory of Energy Science and Engineering, ETH Zürich, 8092 Zürich, Switzerland
[2] School of Chemical Engineering, the University of New South Wales, Sydney, NSW 2052, Australia
[3] Department of Chemistry and Applied Biosciences, ETH Zürich, 8093 Zürich, Switzerland
[4] CNRS, CEMHTI UPR3079, Université Orléans, F-45071 Orléans, France

Emails:
pierre.florian@cnrs-orleans.fr
abdalap@ethz.ch
fedoroal@ethz.ch
muelchri@ethz.ch

FIGURES

Figure S1. N_2 adsorption and desorption isotherms of Ga1-, Ga5- and Ga10-SiO_{2-500} and SiO_{2-500}. 4
Figure S2. BJH pore size distribution of Ga1-, Ga5- and Ga10-SiO_{2-500}, and SiO_{2-500} materials obtained from N_2 isotherms (Figure S1).....4
Figure S3. FTIR spectra of the materials obtained after 2 pulses (black), 10 pulses (red) and 20 pulses (blue) of TMG, deposited onto SiO_{2-500} (green).....5
Figure S4. Transmission FTIR spectra of the hydroxyl region in Ga1-SiO_{2-500} (black), Ga5-SiO_{2-500} (dark red), and Ga10-SiO_{2-500} (blue). 6
Figure S5. HRTEM image of Ga1-SiO_{2-500}......6
Figure S6. HRTEM of Ga10-SiO_{2-500}.....7
Figure S7. ADF-STEM image and EDX mapping of Ga10-SiO_{2-500} (Ga L-green, Si K-red). 7
Figure S8. EDX line scan analysis of Ga10-SiO_{2-500}.....8
Figure S9. XRD patterns of Ga1- (black), Ga5-(red), Ga10-SiO_{2-500} (navy) and SiO_{2-500} (grey).....8
Figure S10. Calcination of Ga10-SiO_{2-500} from 300 °C to 900 °C followed by *in situ* XRD. 9
Figure S11. Comparison between the subtracted XRD patterns of Ga10-SiO_{2-500} calcined at 900 °C with experimental XRD patterns of γ- and β-Ga_2O_3 reproduced from published literature.[2].....10
Figure S12. ^{71}Ga MAS NMR experimental spectra of Ga5-SiO_{2-500} recorded at 20.0 T using a CPMG (purple), CPMG + VOCS (green) and short-pulse Full Hahn echo (blue) acquisition schemes.....10
Figure S13. Comparison of the ^{71}Ga MAS NMR CPMG VOCS spectra of Ga10-SiO_{2-500} at 20.0 T (left) and at 28.2 T (right) showing (enlarged) the individual spectra obtained at each offset.....11
Figure S14. Comparison of the ^{71}Ga 20.0 T QPASS spectra of Ga10-SiO_{2-500} (left) and Ga5-SiO_{2-500} (right) showing the projection along the MAS dimension (blue) compared with the CPMG spectra (red).....11
Figure S15. ^{71}Ga MAS NMR experimental (black) and simulated (red) spectra of Ga10-SiO_{2-500} recorded at 20.0 T or 28.2 T. The various components are shown in different colors: $^{[4]}$Ga (purple), $^{[5]}$Ga (blue) and $^{[6]}$Ga (yellow). 12
Figure S16. XANES of Ga1-, Ga5- and Ga10-SiO_{2-500} compared with Ga-Zeolite-L (Linde) and β-Ga_2O_3. 13
Figure S17. XANES fittings for Ga1-, Ga5-, Ga10-SiO_{2-500} and β-Ga_2O_3. Purple and blue lines correspond to the Gaussian and arctangent functions used for the two different Ga sites. 14
Figure S18. EXAFS fittings of Ga1-SiO_{2-500} in imaginary (left) and real (right) R space.....15
Figure S19. EXAFS fittings of Ga5-SiO_{2-500} in imaginary (left) and real (right) R space.....15
Figure S20. EXAFS fittings of Ga10-SiO_{2-500} in imaginary (left) and real (right) R space.....15
Figure S21. EXAFS fittings of Ga1-SiO_{2-500} in imaginary (left) and real (right) R space including a Ga−Si path. 16
Figure S22. The continuous Cauchy wavelet transform (CCWT) analysis of EXAFS data for Ga1-SiO_{2-500} in the R range: 0.5-4 Å (left) and 2-4 Å (right).....16
Figure S23. The continuous Cauchy wavelet transform (CCWT) analysis of EXAFS data for Ga5-SiO_{2-500} in the R range: 0.5-4 Å (left) and 2-4 Å (right).....17
Figure S24. The continuous Cauchy wavelet transform (CCWT) analysis of EXAFS data for Ga10-SiO_{2-500} in the R range: 0.5-4 Å (left) and 2-4 Å (right).....17
Figure S25. The continuous Cauchy wavelet transform (CCWT) analysis of EXAFS data for β-Ga_2O_3 in the R range: 0.5-4 Å (left) and 2-4 Å (right). 17
Figure S26. PDF and dPDF of Ga1-, Ga5- and Ga10-SiO_{2-500}. 18
Figure S27. (A) X-ray total scattering intensities I(Q) and (B) the reduced structure functions F(Q) of SiO_{2-500}, Ga1-, Ga5- and Ga10-SiO_{2-500}......18
Figure S28. (A)-(C) Peak fittings obtained with SrMise.* (D) Fitted positions of peaks a and b and their area ratio (b/a). 19
Figure S29. RMC fits of Ga1-, Ga5-, Ga10-SiO_{2-500} and the partial contribution of Ga–O and Ga–Ga interatomic pairs. 20

Figure S30. RMC fits of Ga1-, Ga5-, Ga10-SiO_{2-500} and the partial contribution of Ga–O and Ga–Ga interatomic pairs without the introduction of any O-atom point defects...20
Figure S31. Direct detection ^{29}Si NMR experimental (black) and simulated (red) spectra of Ga10-SiO_{2-500}. The various components are shown in different colors: Q_4 (blue), Q_3 (purple) and Si($_{4Ga}$) (yellow)...20
Figure S32. FTIR spectra of Py-Ga1-SiO_{2-500} (black), Py-Ga5-SiO_{2-500} (red), and Py-Ga10-SiO_{2-500} (blue) after desorption of Py at RT. PyH^+ and Py-L are highlighted in light gray and brown, respectively. L indicates a Lewis acid site...21
Figure S33. Isobutene formation rates normalized by the respective Ga loadings and surface areas (mmol i-C_4H_8 h^{-1} g_{Ga}^{-1} m^{-2}) over 20 h TOS on Ga1-, Ga5- and Ga10-SiO_{2-500}..................................23
Figure S34. Isobutane conversion and product selectivity to C_4H_8 (yellow), C_3H_6 (green) and alkanes (blue) on Ga1-SiO_{2-500} over 20 h TOS. 23
Figure S35. Isobutane conversion and product selectivity of C_4H_8 (yellow), C_3H_6 (green) and alkanes (blue) on Ga5-SiO_{2-500} over 20 h TOS. 24
Figure S36. Isobutane conversion and product selectivity of C_4H_8 (yellow), C_3H_6 (green) and alkanes (blue) on Ga10-SiO_{2-500} over 20 h TOS. 24
Figure S37. Formation rates to cracking products (combined rates to methane and propene, µmol h^{-1}) on Ga1-, Ga5-, Ga10-SiO_{2-500} and SiC..25
Figure S38. Space-time-yield (µmol h^{-1} g_{cat}^{-1}) of (a) benzene, (b) toluene and (c) xylenes over 20 h TOS for Ga1-, Ga5- and Ga10-SiO_{2-500}..25
Figure S39. Space-time-yield (mmol h^{-1} g_{cat}^{-1}) of isobutene, benzene, toluene and C_8 aromatics (ethylbenzene and *m*-, *p*-, *o*-xylene) and carbon balance on Ga1-(black), Ga5-(red), Ga10-SiO_{2-500} (blue) over 20 h (4 × 5 h) TOS including three regeneration cycles after every 5 h...........................26
Figure S40. Isobutene formation rates normalized by the respective Ga loadings and surface areas (mmol i-C_4H_8 h^{-1} g_{Ga}^{-1} m^{-2}) over 20 h TOS on Ga1-, Ga5- and Ga10-SiO_{2-500}..................................27
Figure S41. Space-time-yield (mmol i-C_4H_8 h^{-1} g_{cat}^{-1}) and selectivity of isobutene over 20 h TOS on Ga1-, Ga5- and Ga10-SiO_{2-500}..27
Figure S42. Initial product selectivity and i-C_4H_{10} conversion on Ga1-, Ga5-, and Ga10-SiO_{2-500}.....28
Figure S43. Comparison between ^{15}N DNP SENS spectra of Py-Al1,5,10-SiO_{2-500} (a) and Py-Ga1,5,10-SiO_{2-500} (b) after desorption of pyridine at 150 °C. Note that the ^{15}N DNP SENS spectra of Py-Al1,5,10-SiO_{2-500} have been reported by us previously and are reproduced here for comparison.[8] Copyright 2021, the American Chemical Society (ACS)...29

TABLES

Table S1. N_2 physisorption data, including BET surface area, pore volume and pore diameter for SiO_{2-500} and Ga1-, Ga5- and Ga10-SiO_{2-500}. 5
Table S2. ^{71}Ga MAS NMR parameters derived from the simulations of the one-pulse quantitative spectra of Ga10-SiO_{2-500}..13
Table S3. XANES fitting results...14
Table S4. Results of the EXAFS fittings of Ga1-SiO_{2-500} including Ga−Si paths. 16
Table S5. Constraints used in the reverse Monte Carlo (RMC) simulation. 19
Table S6. Fitting results of the ^{15}N DNP-enhanced NMR spectra of Py-Ga1,5,10-SiO_{2-500}................21
Table S7. Results from BET, ICP and catalytic tests. Catalytic data is presented after 7 min TOS and, in parentheses, after 20 h TOS. Reaction condition: 10% of i-C_4H_{10} in N_2, WHSV = 8.5 h^{-1}, T = 550 °C...26
Table S8. Results from BET, ICP and catalytic tests. Catalytic data is presented after 7 min TOS and, in parentheses, after 5h TOS. Reaction condition: 10% of i-C_4H_{10} in N_2, WHSV = 8.5 h^{-1}, T = 500 °C. 28

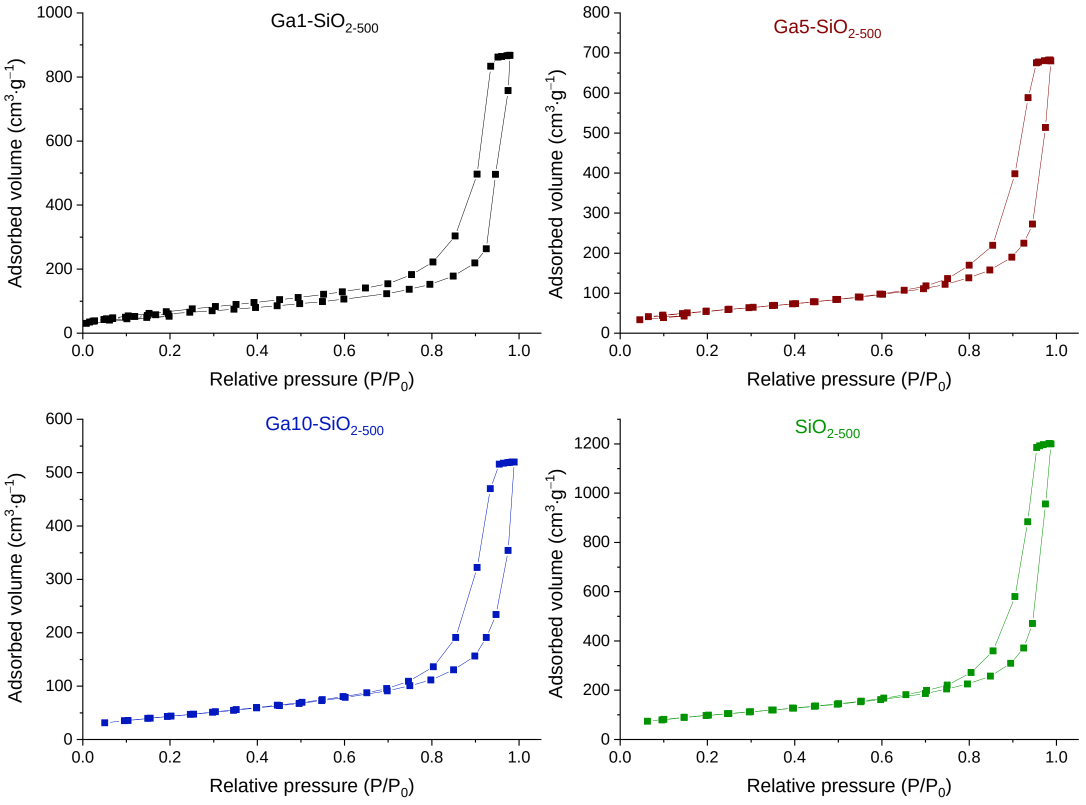

Figure S1. N_2 adsorption and desorption isotherms of Ga1-, Ga5- and Ga10-$SiO_{2\text{-}500}$ and $SiO_{2\text{-}500}$.

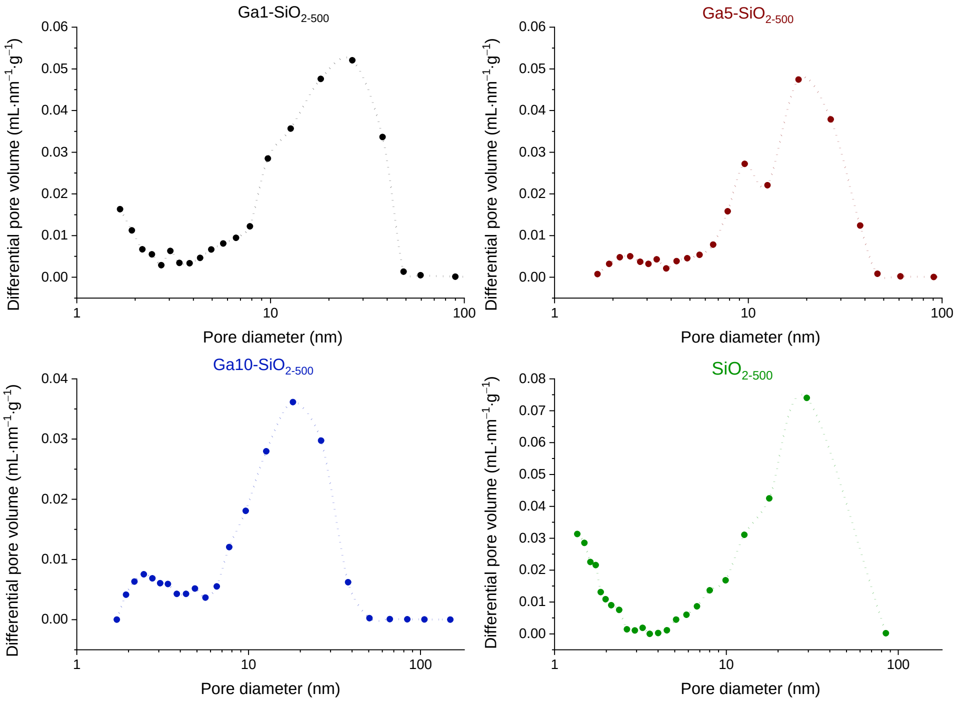

Figure S2. BJH pore size distribution of Ga1-, Ga5- and Ga10-$SiO_{2\text{-}500}$, and $SiO_{2\text{-}500}$ materials obtained from N_2 isotherms (Figure S1).

Table S1. N_2 physisorption data, including BET surface area, pore volume and pore diameter for SiO_{2-500} and Ga1-, Ga5- and Ga10-SiO_{2-500}.

Materials	BET surface area (m^2 g^{-1})	Pore volume (cm^3 g^{-1})	Pore diameter (nm)
SiO_{2-500}	374	2.0	29.4
Ga1-SiO_{2-500}	293	1.5	26.4
Ga5-SiO_{2-500}	202	1.0	20.3
Ga10-SiO_{2-500}	160	0.8	18.1

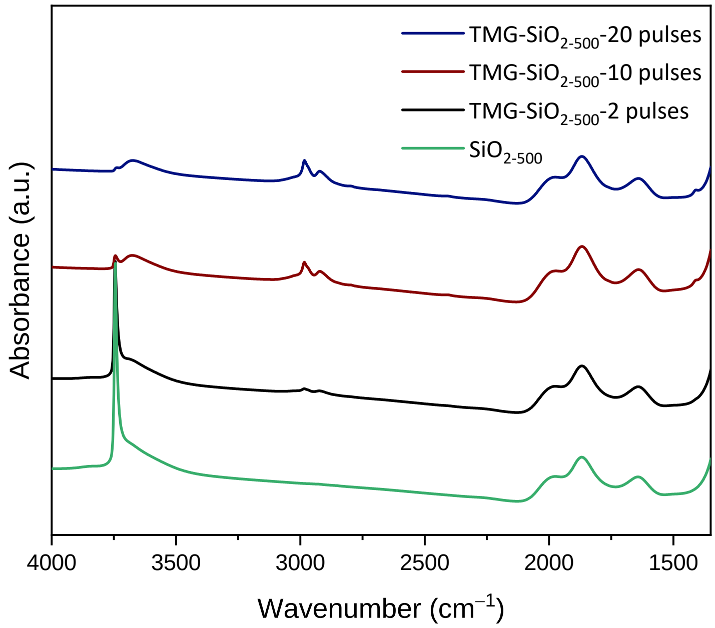

Figure S3. FTIR spectra of the materials obtained after 2 pulses (black), 10 pulses (red) and 20 pulses (blue) of TMG, deposited onto SiO_{2-500} (green).

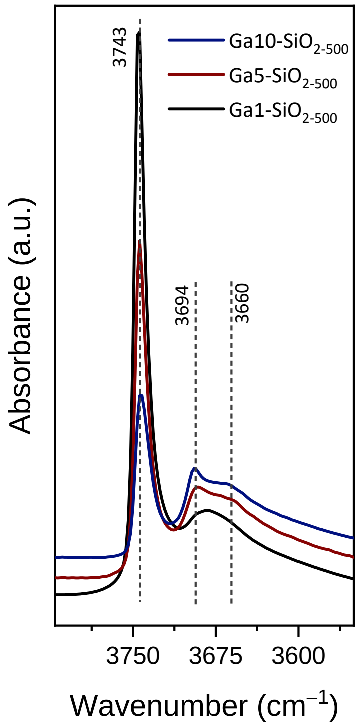

Figure S4. Transmission FTIR spectra of the hydroxyl region in Ga1-SiO_{2-500} (black), Ga5-SiO_{2-500} (dark red), and Ga10-SiO_{2-500} (blue).

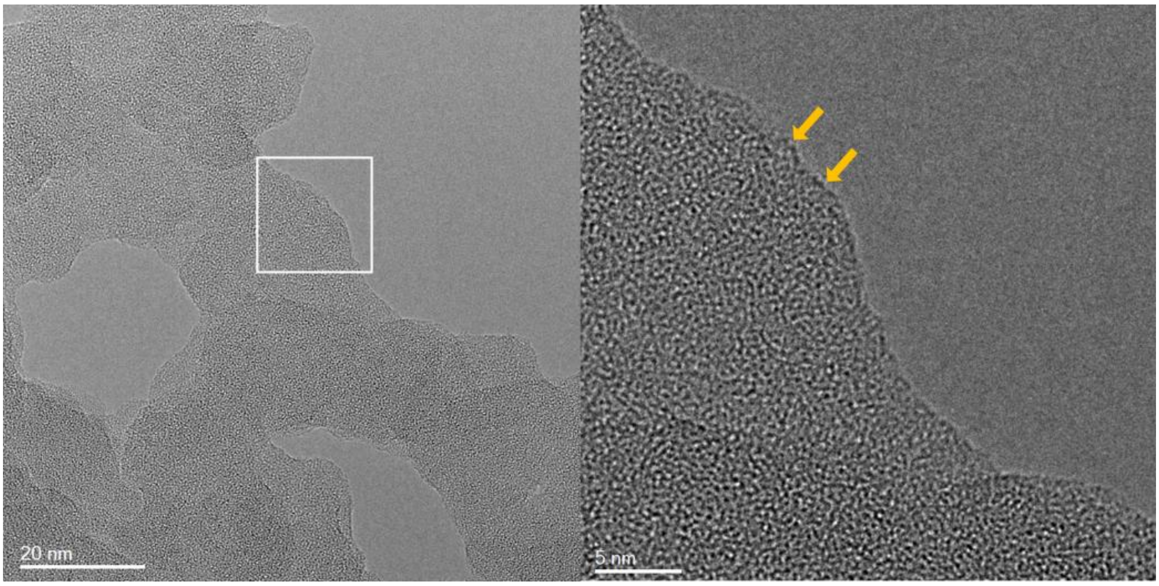

Figure S5. HRTEM image of Ga1-SiO_{2-500}.

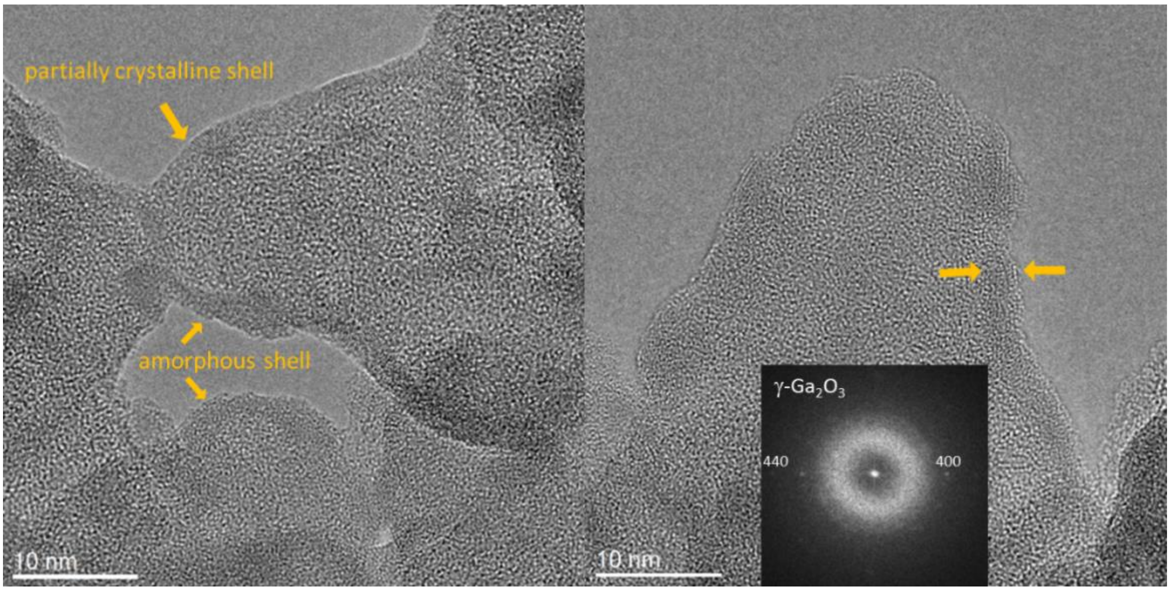

Figure S6. HRTEM of Ga10-SiO_{2-500}.

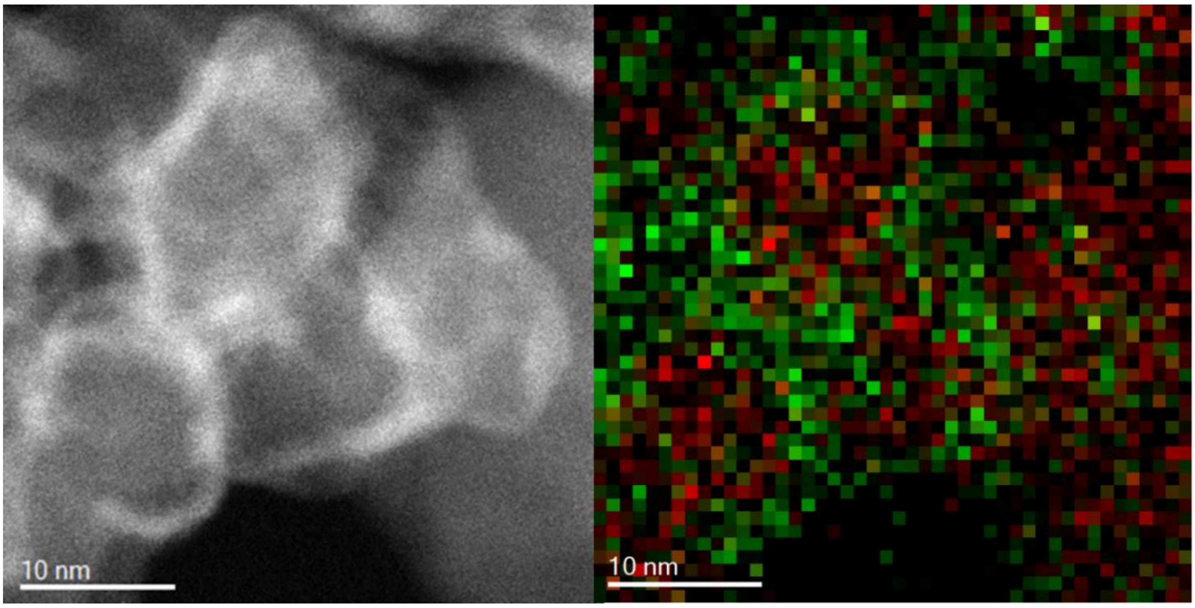

Figure S7. ADF-STEM image and EDX mapping of Ga10-SiO_{2-500} (Ga L-green, Si K-red).

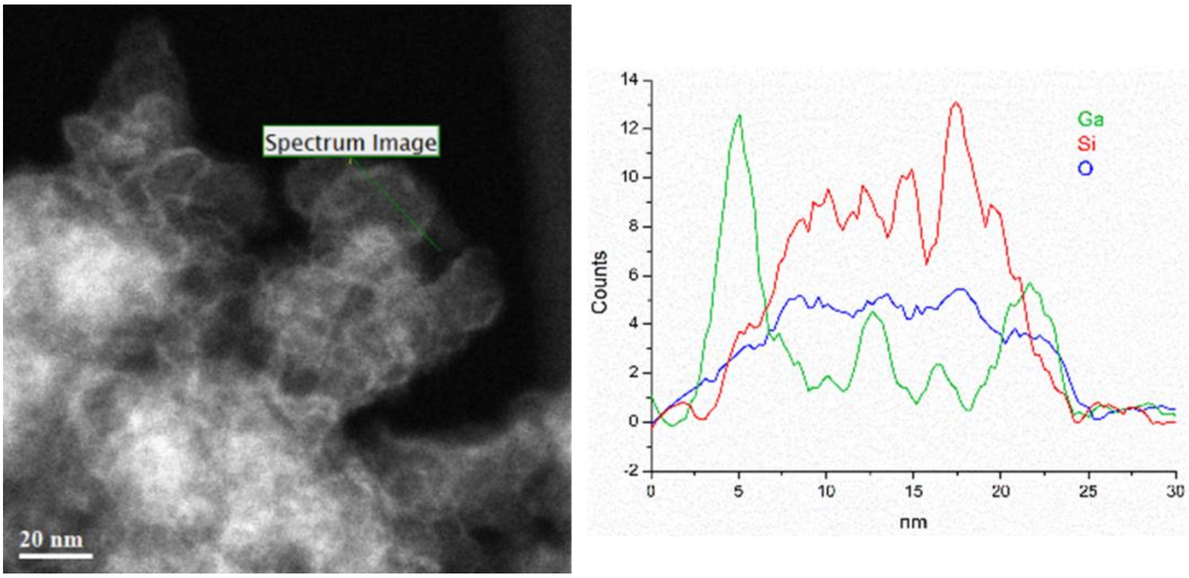

Figure S8. EDX line scan analysis of Ga10-$SiO_{2\text{-}500}$.

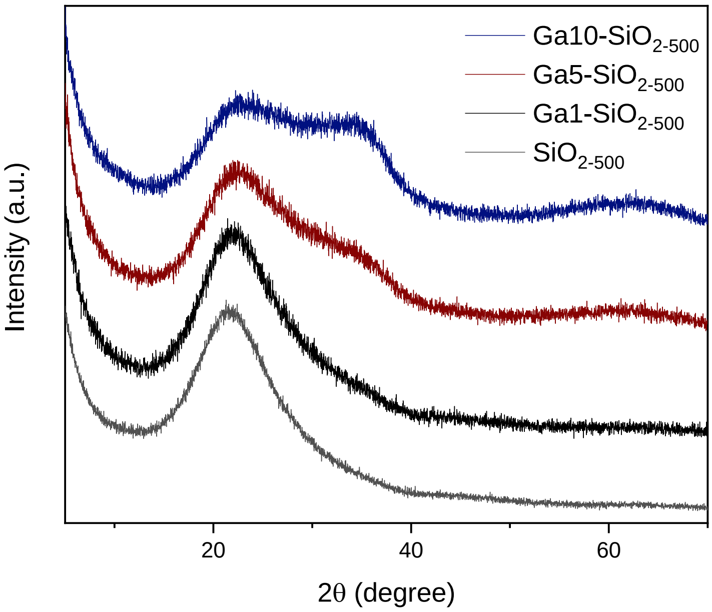

Figure S9. XRD patterns of Ga1- (black), Ga5-(red), Ga10-$SiO_{2\text{-}500}$ (navy) and $SiO_{2\text{-}500}$ (grey).

In situ XRD

The calcination of Ga10-$SiO_{2\text{-}500}$ from room temperature to 900 °C followed by *in situ* XRD (synthetic air, 50 ml min^{-1},10 °C min^{-1}), shows the formation of broad (owing to a short coherence length) Bragg peaks starting at ca. 820 °C; these peaks can be assigned to γ-Ga_2O_3 (Figure S11). It has been reported that amorphous gallia films deposited onto fused SiO_2 crystallize to β-Ga_2O_3 at about 900 °C.[1] However, our *in situ* XRD experiment suggests that in Ga10-$SiO_{2\text{-}500}$ the amorphous gallia shells on amorphous SiO_2 first crystallizes in a metastable γ-Ga_2O_3 polymorph, possibly, due to the partial formation of a gallosilicate phase. No β-Ga_2O_3 was observed by XRD in the studied temperature range (i.e., up to 900 °C), yet the presence of small (e.g., < 2 nm) nanodomains of β-Ga_2O_3 cannot be discarded by these measurements.[2] Overall, the results discussed above suggest that Ga1-, Ga5 and Ga10-$SiO_{2\text{-}500}$ contain an amorphous Ga-rich shell that covers the silica core (partial coverage in the case of Ga1-$SiO_{2\text{-}500}$). The shell remains amorphous, for Ga10-$SiO_{2\text{-}500}$, up to ca. 820 °C.

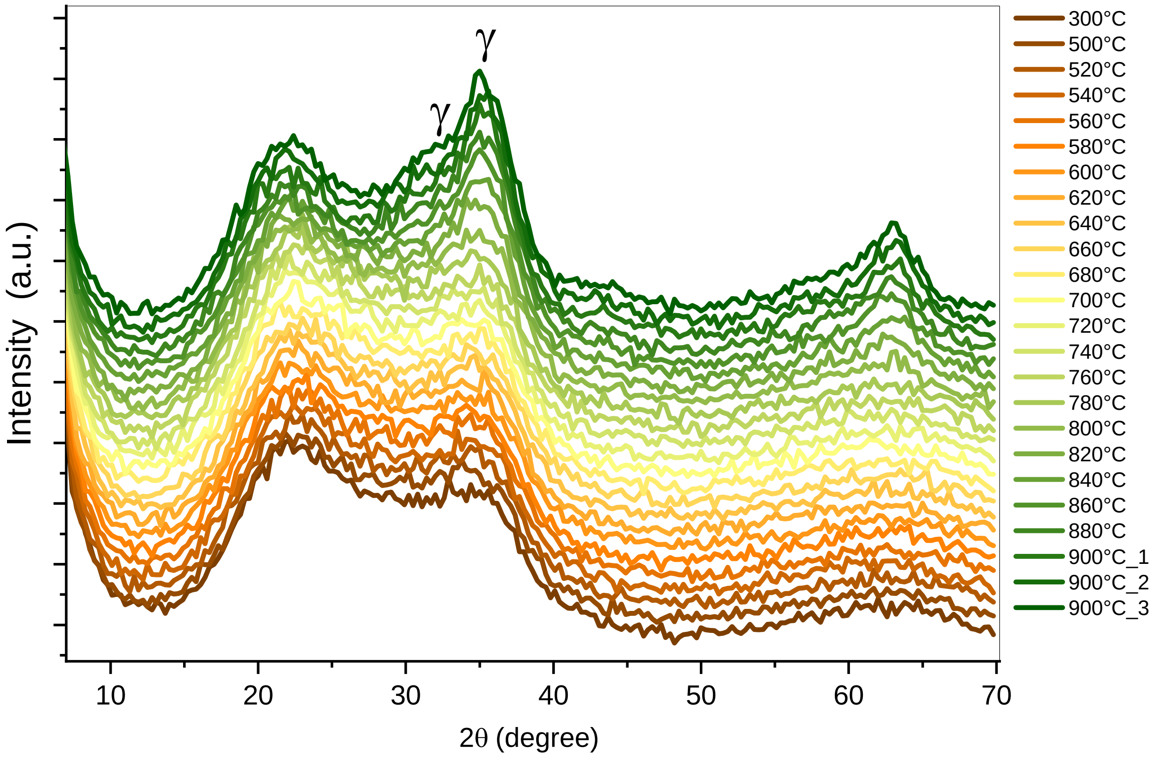

Figure S10. Calcination of Ga10-$SiO_{2\text{-}500}$ from 300 °C to 900 °C followed by *in situ* XRD.

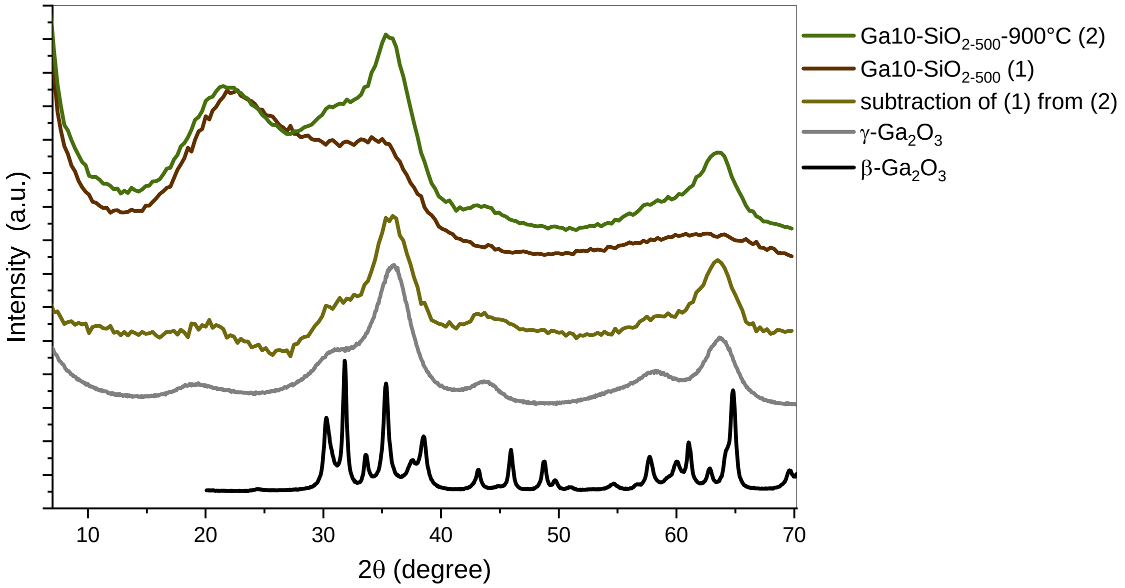

Figure S11. Comparison between the subtracted XRD patterns of Ga10-SiO_{2-500} calcined at 900 °C with experimental XRD patterns of γ- and β-Ga_2O_3 reproduced from published literature.[2]

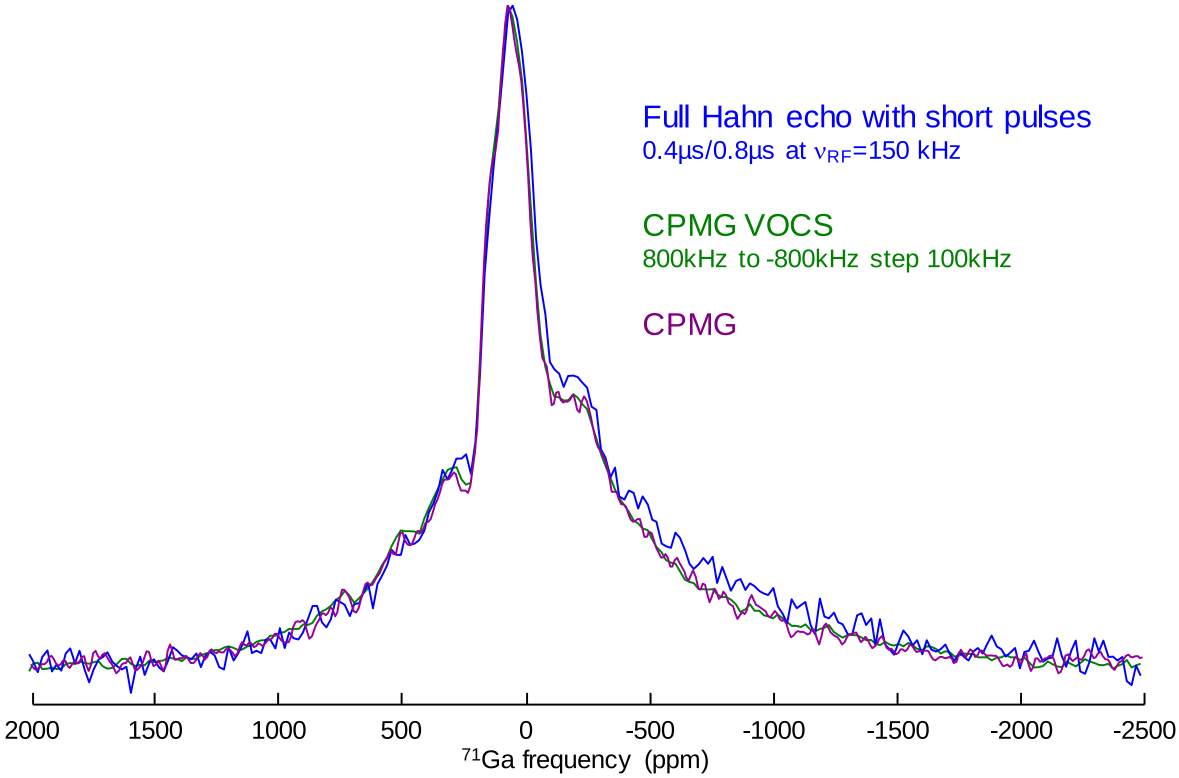

Figure S12. ^{71}Ga MAS NMR experimental spectra of Ga5-SiO_{2-500} recorded at 20.0 T using a CPMG (purple), CPMG + VOCS (green) and short-pulse Full Hahn echo (blue) acquisition schemes.

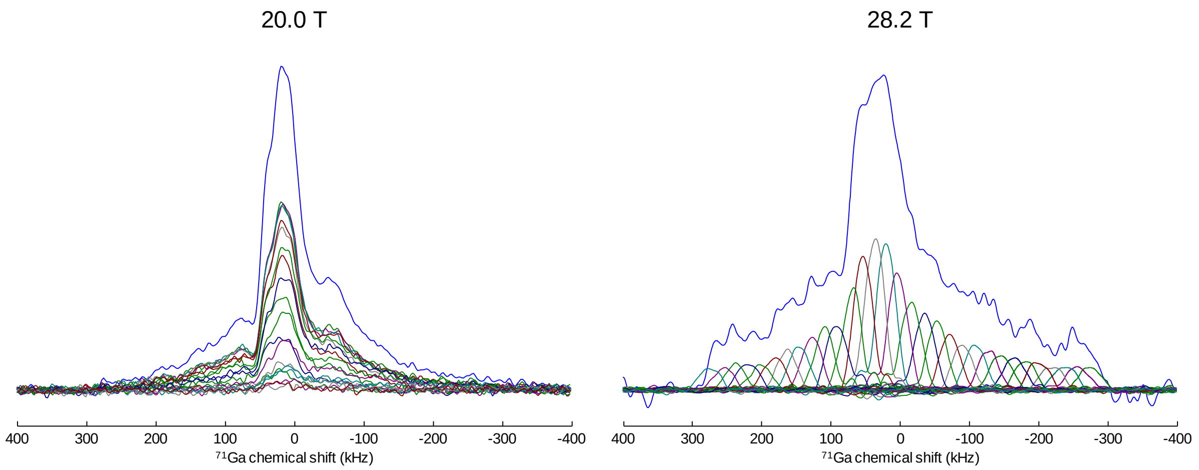

Figure S13. Comparison of the ^{71}Ga MAS NMR CPMG VOCS spectra of Ga10-SiO_{2-500} at 20.0 T (left) and at 28.2 T (right) showing (enlarged) the individual spectra obtained at each offset.

Note that small line shape distortions are seen between 0 kHz and 100 kHz in the 28.2 T experiment.

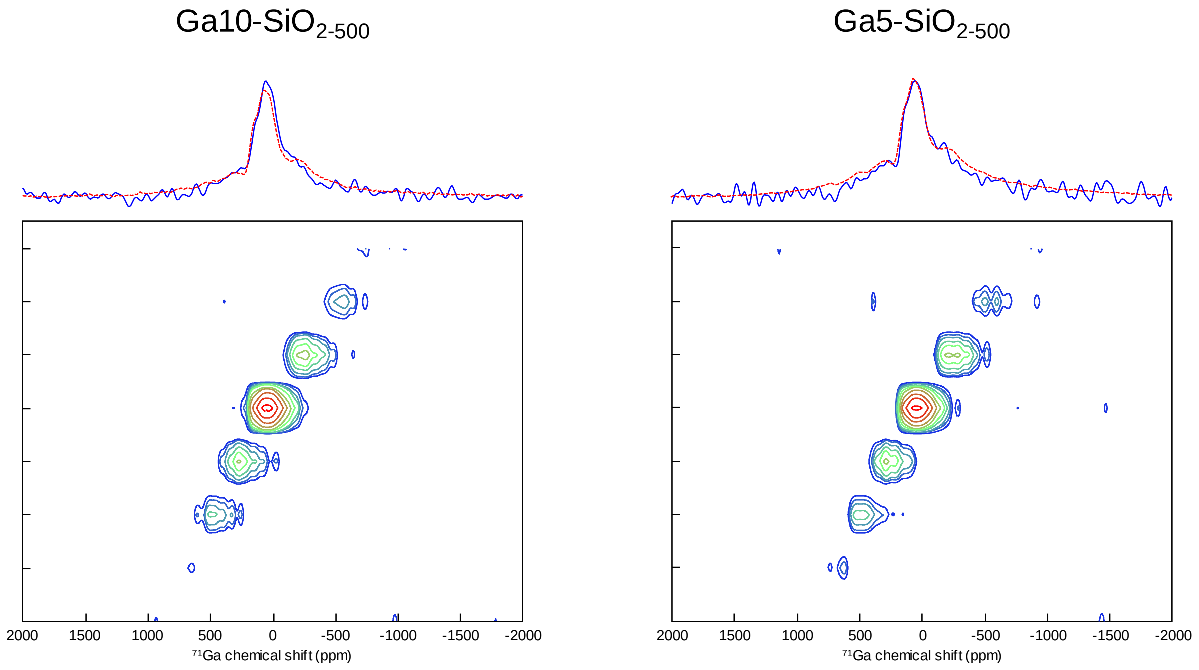

Figure S14. Comparison of the ^{71}Ga 20.0 T QPASS spectra of Ga10-SiO_{2-500} (left) and Ga5-SiO_{2-500} (right) showing the projection along the MAS dimension (blue) compared with the CPMG spectra (red).

This experiment leads to the loss of intensities on the right and left side of the main peak, particularly for Ga5-SiO_{2-500}.

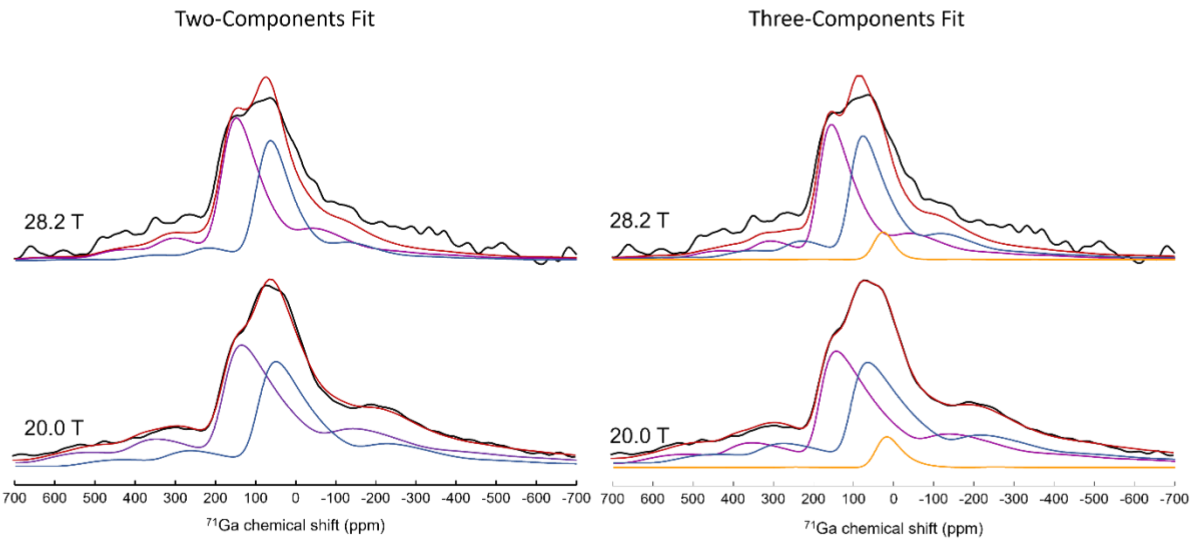

Figure S15. ^{71}Ga MAS NMR experimental (black) and simulated (red) spectra of Ga10-SiO_{2-500} recorded at 20.0 T or 28.2 T. The various components are shown in different colors: [4]Ga (purple), [5]Ga (blue) and [6]Ga (yellow).

Table S2. ^{71}Ga MAS NMR parameters derived from the simulations of the one-pulse quantitative spectra of Ga10-SiO_{2-500}.

site	%	$\bar{\delta}_{iso}$, (ppm)	$\Delta\delta_{iso}$ (ppm)	$\bar{C}_Q$ (MHz)
two-components fit on Ga10-SiO_{2-500}				
$^{[4]}Ga$	60 ± 3	188 ± 3	58	11.0 ± 0.1
$^{[5]}Ga$	40 ± 3	100 ± 3	58	9.6 ± 0.1
three-components fit on Ga10-SiO_{2-500}				
$^{[4]}Ga$	50 ± 3	192± 1	46	10.6 ± 0.1
$^{[5]}Ga$	47 ± 3	114 ± 3	46	10.9 ± 0.1
$^{[6]}Ga$	4 ± 1	44± 4	46	5.0 ± 0.2

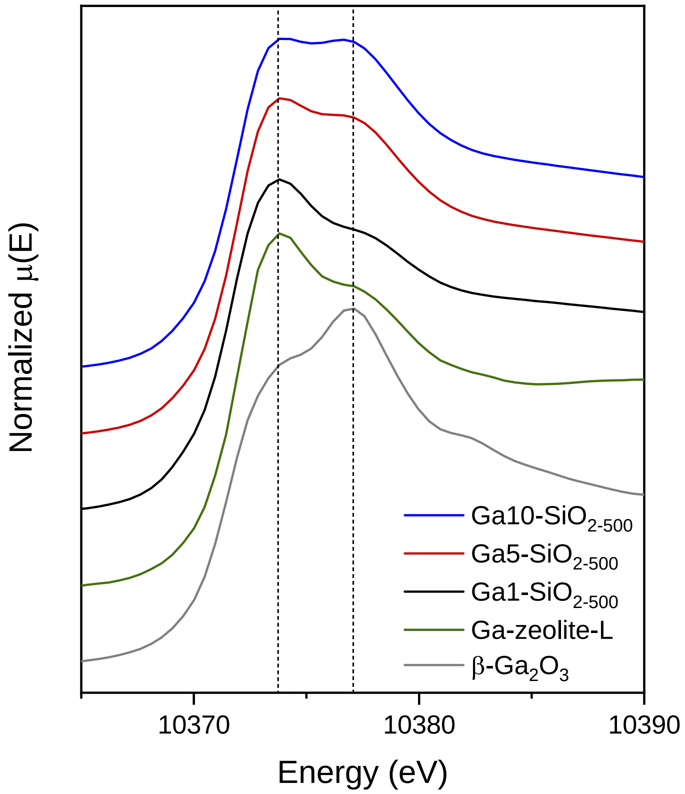

Figure S16. XANES of Ga1-, Ga5- and Ga10-SiO_{2-500} compared with Ga-Zeolite-L (Linde) and β-Ga_2O_3.

Table S3. XANES fitting results.

Material	$^{[4]}Ga$			$^{[5]}Ga/^{[6]}Ga$			*$^{[4]}Ga\%$
	Position (eV)	FWHM	$Area_{(1)}$	Position (eV)	FWHM	$Area_{(2)}$	
Ga1-$SiO_{2\text{-}500}$	10373.2	1.7	4.4	10377.0	1.8	1.5	75
Ga5-$SiO_{2\text{-}500}$	10373.2	1.6	4.3	10376.5	1.8	2.6	62
Ga10-$SiO_{2\text{-}500}$	10373.2	1.8	4.3	10376.5	1.8	3.2	57
β-Ga_2O_3	10373.2	2	4.5	10377.3	2	4.5	50

*Relative abundance of $^{[4]}Ga$ calculated according to: $(Area_{(1)})/[(Area_{(1)})+(Area_{(2)})]\cdot 100$

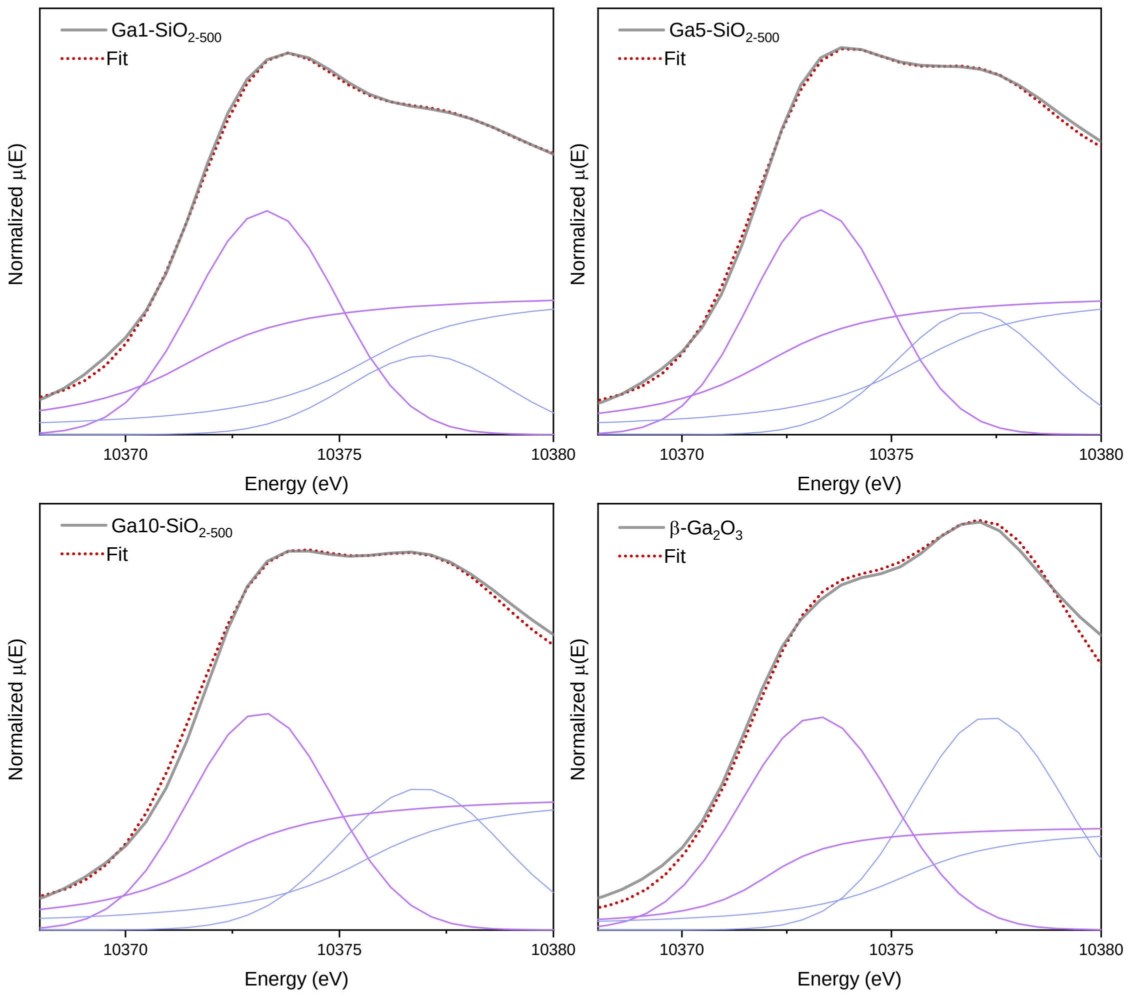

Figure S17. XANES fittings for Ga1-, Ga5-, Ga10-$SiO_{2\text{-}500}$ and β-Ga_2O_3. Purple and blue lines correspond to the Gaussian and arctangent functions used for the two different Ga sites.

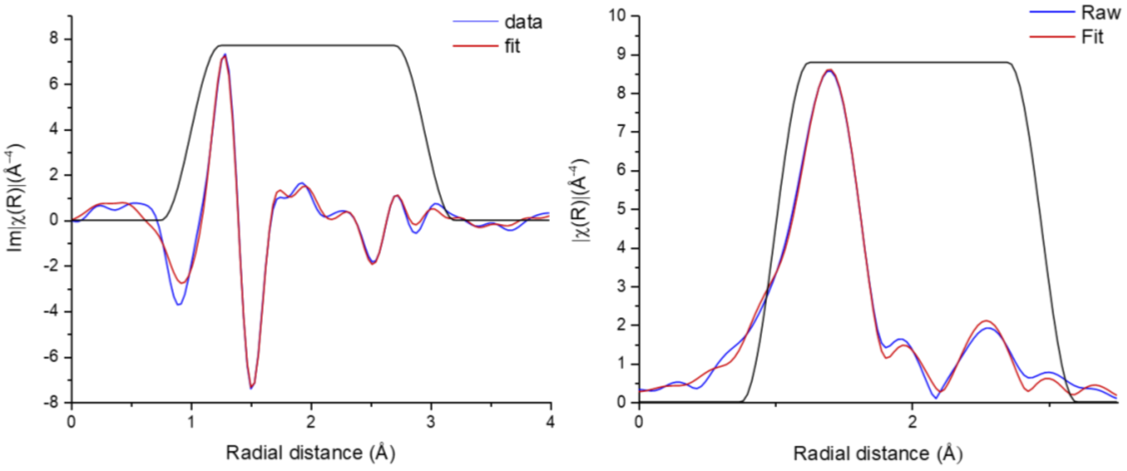

Figure S18. EXAFS fittings of Ga1-SiO_{2-500} in imaginary (left) and real (right) R space.

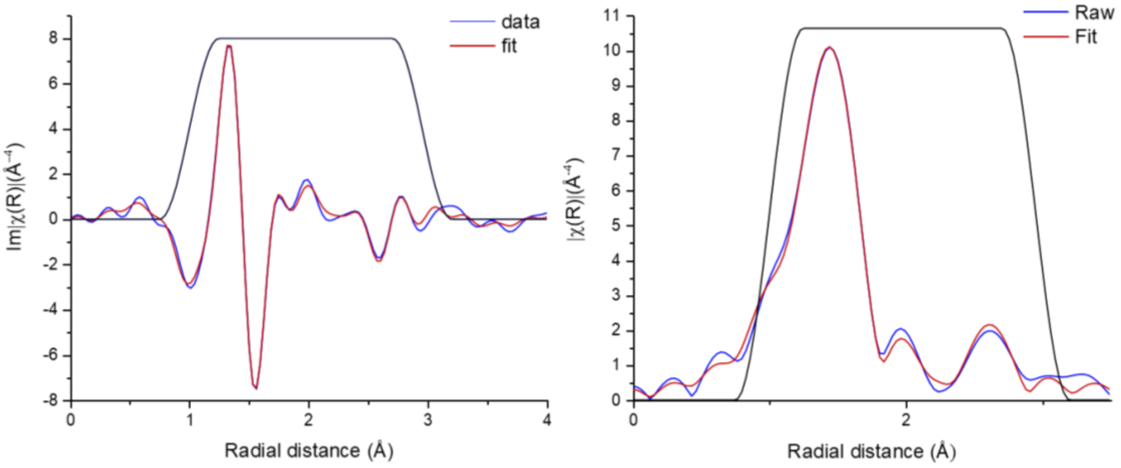

Figure S19. EXAFS fittings of Ga5-SiO_{2-500} in imaginary (left) and real (right) R space.

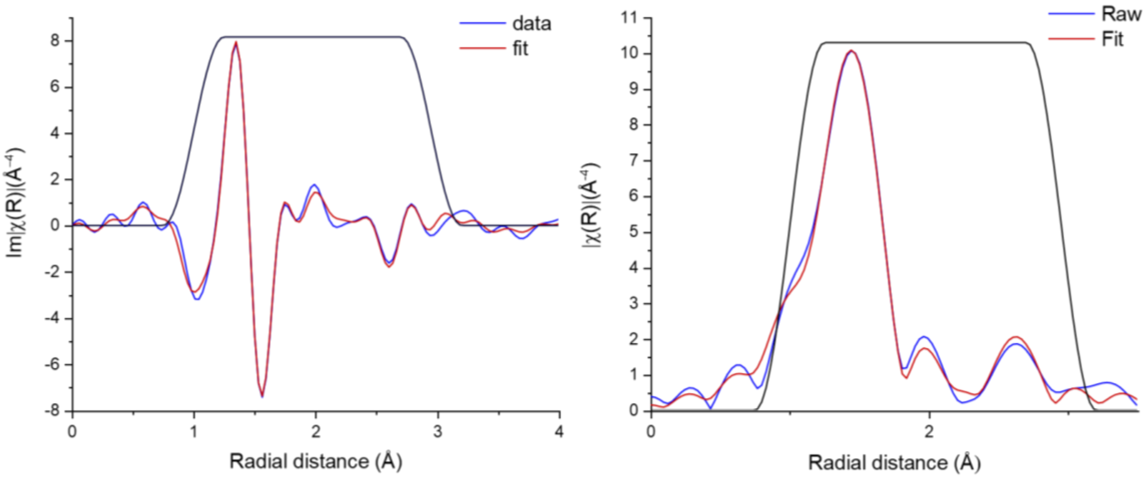

Figure S20. EXAFS fittings of Ga10-SiO_{2-500} in imaginary (left) and real (right) R space.

Table S4. Results of the EXAFS fittings of Ga1-SiO_{2-500} including Ga–Si paths.

Material	Neighbour	CN	r (Å)	σ^2 (Å^2)	E_0 (eV)	R-factor
Ga1-SiO_{2-500}	O	4.4(3)	1.83(1)	0.009(1)	6(1)	0.005
	Ga	0.7(1)	2.80(1)	0.076*		
Ga1-SiO_{2-500}	O	4.4(2)	1.83(1)	0.007(1)	6(1)	0.003
	Si	0.2(2)	2.60(3)	0.0076*		
	Ga	0.5(1)	2.80(2)	0.0076*		

All samples were measured at ambient temperature. S_0^2 was fitted to 1 as obtained by fitting a β-Ga_2O_3 reference. CN stands for the coordination number. *Debye-Waller factors σ^2 were constrained to the same value for the Ga–Ga and Ga–Si paths.

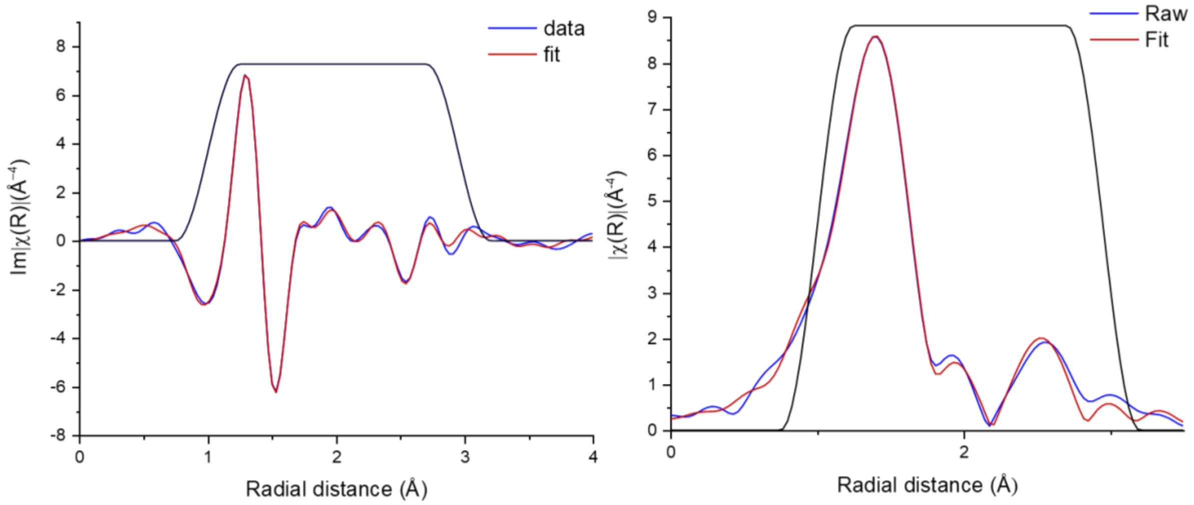

Figure S21. EXAFS fittings of Ga1-SiO_{2-500} in imaginary (left) and real (right) R space including a Ga–Si path.

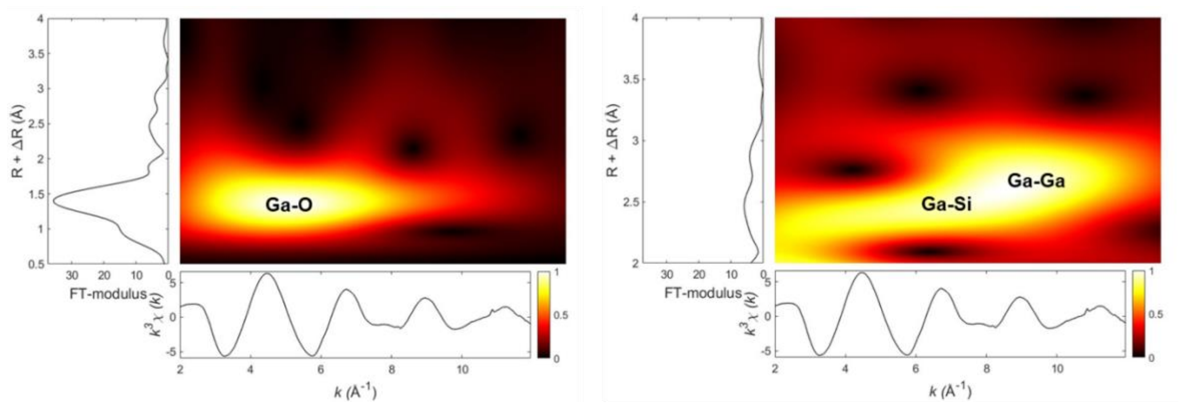

Figure S22. The continuous Cauchy wavelet transform (CCWT) analysis of EXAFS data for Ga1-SiO_{2-500} in the R range: 0.5-4 Å (left) and 2-4 Å (right).

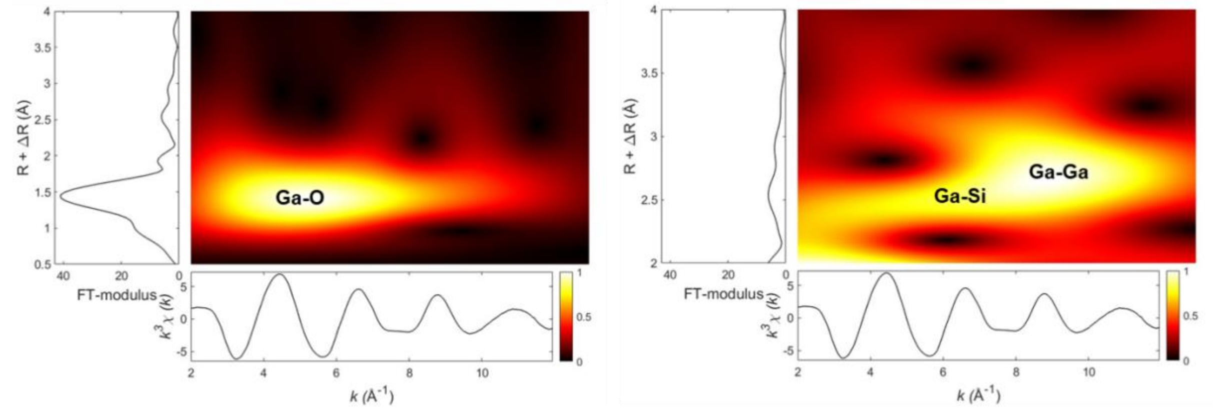

Figure S23. The continuous Cauchy wavelet transform (CCWT) analysis of EXAFS data for Ga5-$SiO_{2\text{-}500}$ in the R range: 0.5-4 Å (left) and 2-4 Å (right).

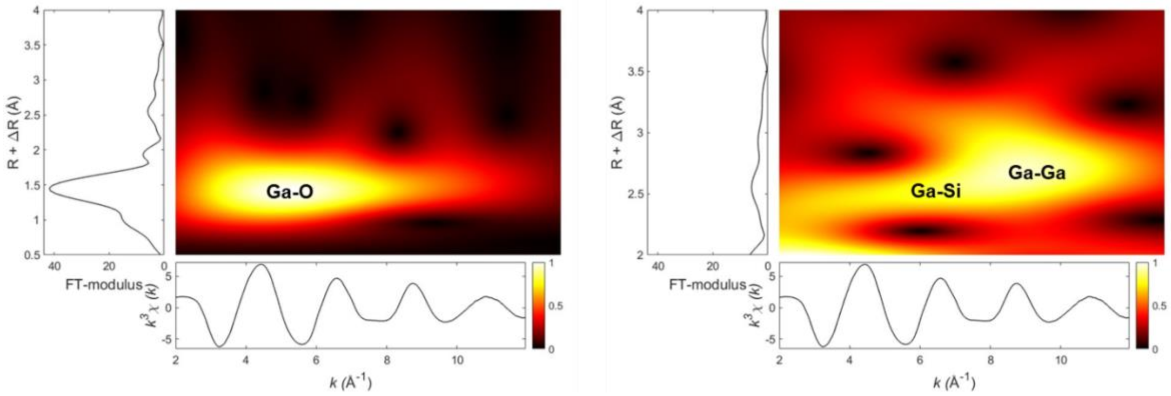

Figure S24. The continuous Cauchy wavelet transform (CCWT) analysis of EXAFS data for Ga10-$SiO_{2\text{-}500}$ in the R range: 0.5-4 Å (left) and 2-4 Å (right).

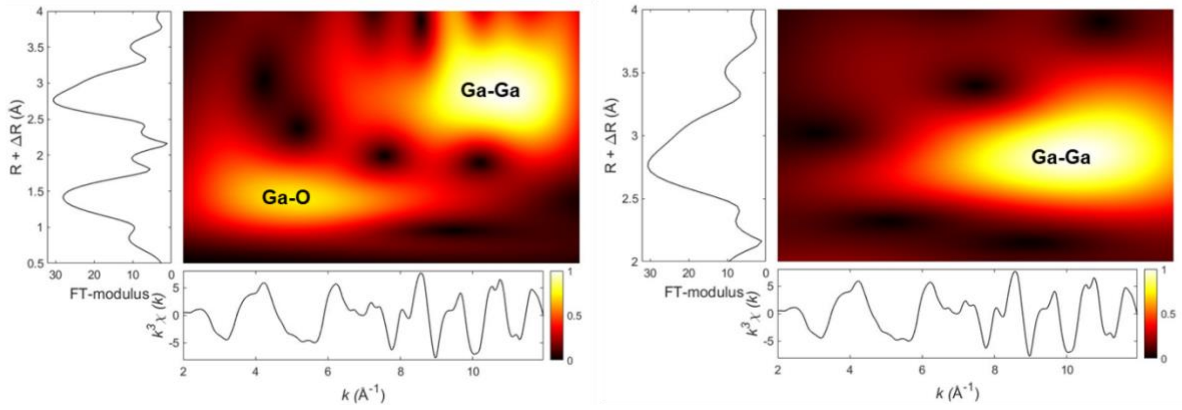

Figure S25. The continuous Cauchy wavelet transform (CCWT) analysis of EXAFS data for β-Ga_2O_3 in the R range: 0.5-4 Å (left) and 2-4 Å (right).

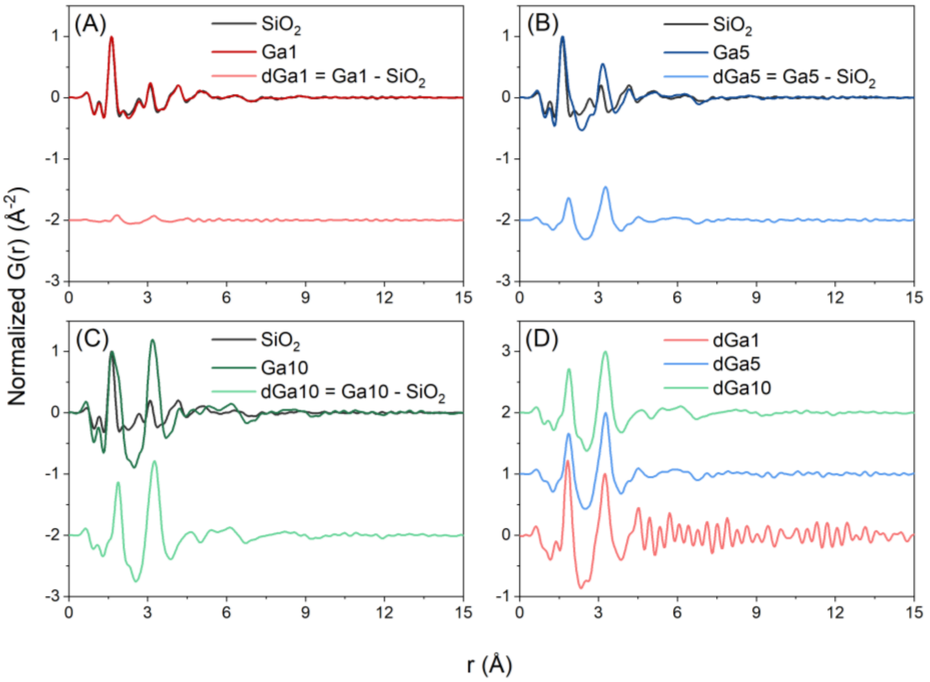

Figure S26. PDF and dPDF of Ga1-, Ga5- and Ga10-SiO_{2-500}.

The traces in Figure S26 were obtained by subtraction of the PDF of SiO_{2-500} (black line in A-C) from the PDF of Ga1- (A), Ga5- (B) and Ga10-SiO_{2-500} (C). For the subtraction, all of the PDFs were normalized to the peak at ca. 1.5-1.6 Å (due to the Si−O distance).

Due to the increasing Ga content in Ga1 < Ga5 < Ga10, the magnitude of the dPDF also increases in this order. For comparison, the intensity of dPDF in (D) were normalized to the maximum of the peak at ca. 3.2-3.3 Å.

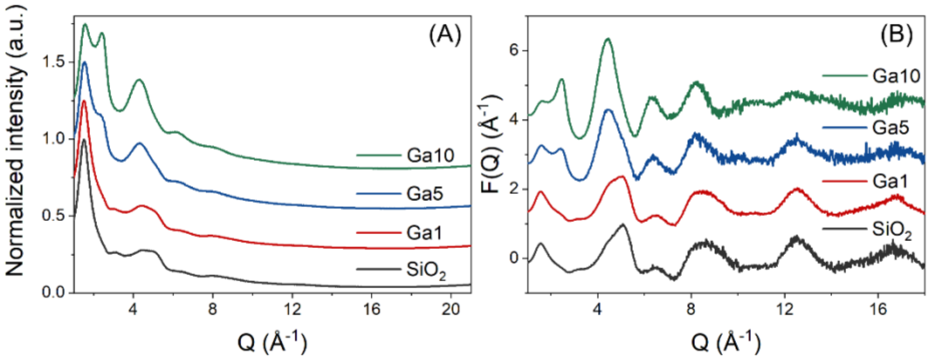

Figure S27. (A) X-ray total scattering intensities I(Q) and (B) the reduced structure functions F(Q) of SiO_{2-500}, Ga1-, Ga5- and Ga10-SiO_{2-500}.

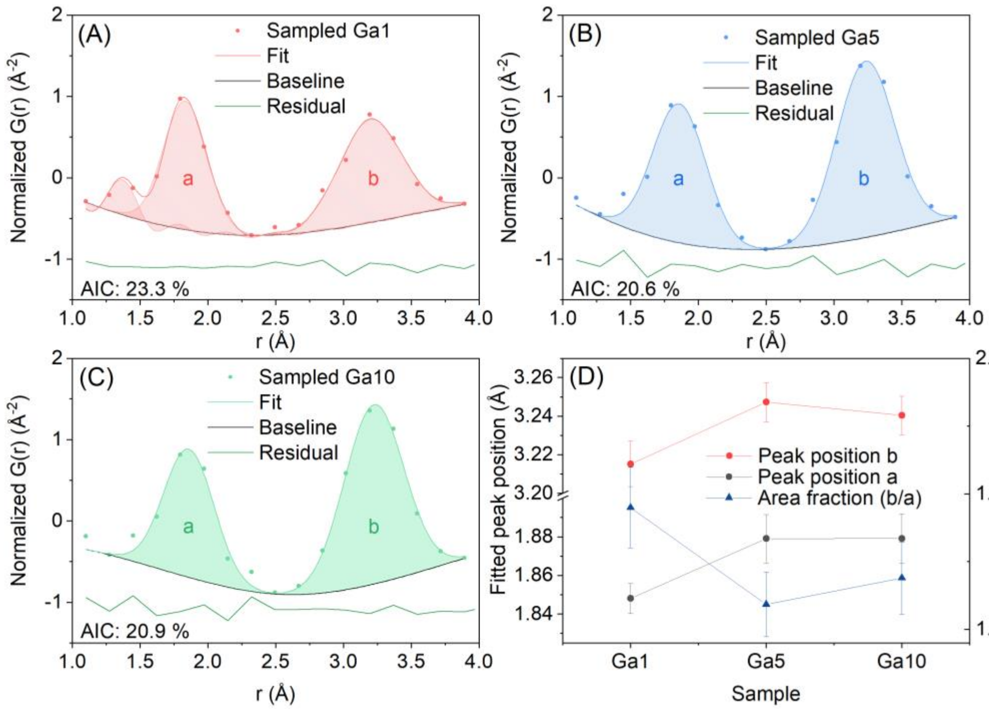

Figure S28. (A)-(C) Peak fittings obtained with SrMise.* (D) Fitted positions of peaks a and b and their area ratio (b/a).

* The quality of the peak fittings in SrMise is assessed using the Akaike information criterion (AIC), which is implemented in SrMise as AIC = χ^2 + 2k where χ^2 is the standard chi-square error ($\chi^2 = \sum_i \varepsilon_i^2/\sigma_i^2$ with residuals ε_i and uncertainties σ_i of the i-th data point) and k is the number of free parameters in the model. A lower value of AIC indicates a more plausible model, however only differences between the AIC of fittings of the same data are meaningful.[3]

Table S5. Constraints used in the reverse Monte Carlo (RMC) simulation.

Sr #	Atomic pair	Intramolecular distance constraint	Avg. CN constraint
1	Ga–O	≥ 1.4 Å	4.0 – 6.0
2	Ga–Ga	≥ 2.65 Å	Not constrained
3	Ga–Si	≥ 2.5 Å	Not constrained

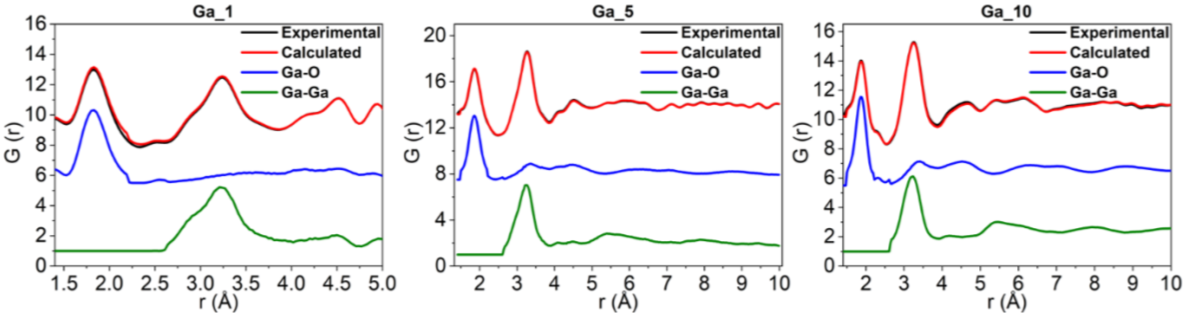

Figure S29. RMC fits of Ga1-, Ga5-, Ga10-$SiO_{2\text{-}500}$ and the partial contribution of Ga–O and Ga–Ga interatomic pairs.

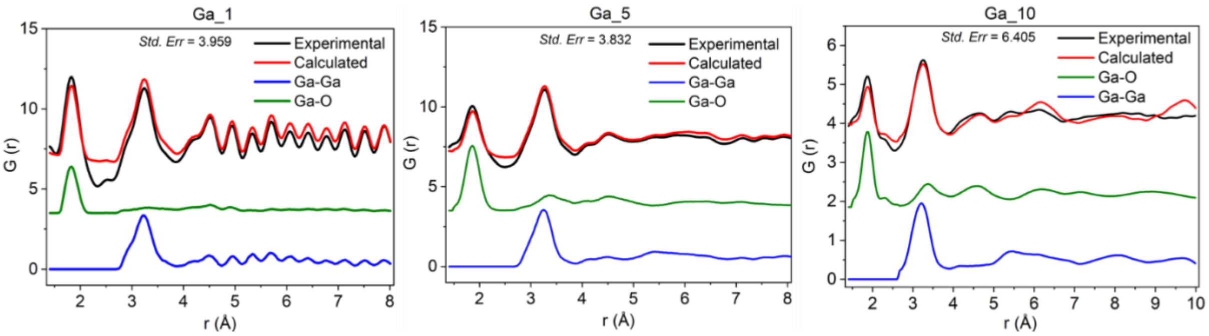

Figure S30. RMC fits of Ga1-, Ga5-, Ga10-$SiO_{2\text{-}500}$ and the partial contribution of Ga–O and Ga–Ga interatomic pairs without the introduction of any O-atom point defects.

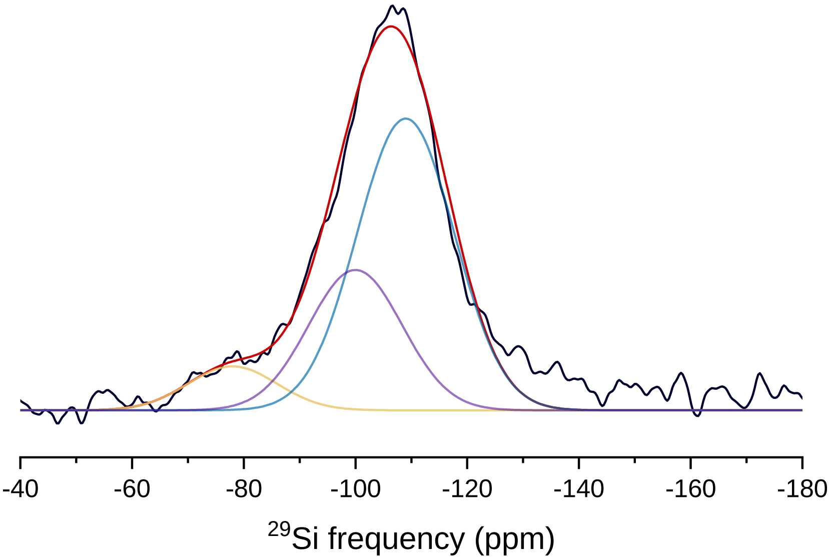

Figure S31. Direct detection ^{29}Si NMR experimental (black) and simulated (red) spectra of Ga10-$SiO_{2\text{-}500}$. The various components are shown in different colors: Q_4 (blue), Q_3 (purple) and $Si_{(4Ga)}$ (yellow).

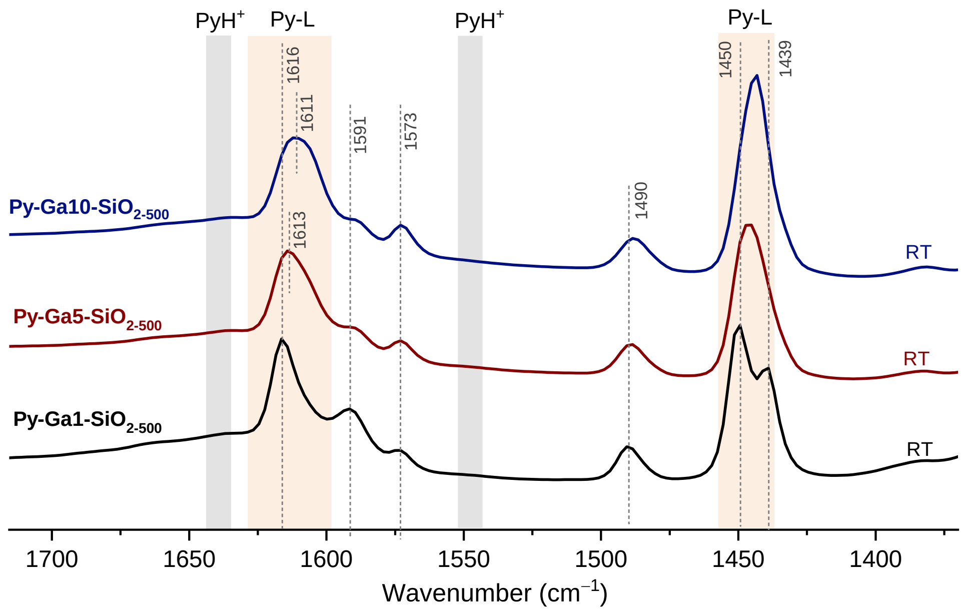

Figure S32. FTIR spectra of Py-Ga1-SiO_{2-500} (black), Py-Ga5-SiO_{2-500} (red), and Py-Ga10-SiO_{2-500} (blue) after desorption of Py at RT. PyH^+ and Py-L are highlighted in light gray and brown, respectively. L indicates a Lewis acid site.

Py-Ga1-SiO_{2-500} features relatively narrow Py-L bands at ca. 1616 cm^{-1} at T_{des} = RT (Figure S32), yet with an increasing number of ALD cycles, this band broadens and shifts to ca. 1613 cm^{-1} in Ga5-SiO_{2-500} and ca. 1611 cm^{-1} in Ga10-SiO_{2-500}. The broadening and the blue shift of the band suggest the presence of a larger number of distinct LAS of milder acidity on Ga5- and Ga10-SiO_{2-500} relative to Ga1-SiO_{2-500}. Mild or weak BAS due to H-Py are identified by the IR bands at 1591 and ca. 1439 cm^{-1} that are found in all prepared Ga-SiO_{2-500} materials (Figure S32). These bands disappear after Py desorption at 150 °C (Figure 5a).

Table S6. Fitting results of the ^{15}N DNP-enhanced NMR spectra of Py-Ga1,5,10-SiO_{2-500}.

Site	Py-Ga1-SiO_{2-500}		Py-Ga5-SiO_{2-500}		Py-Ga10-SiO_{2-500}	
	position (ppm)	height (a.u.)	position (ppm)	height (a.u.)	position (ppm)	height (a.u.)
Py-$L_{(1)}$	250	103	252	120	256	290
Py-$L_{(2)}$	233	341	235	235	237	420
$PyH^+_{(1)}$	216	63	221	55	214	52
$PyH^+_{(2)}$	201	325	201	311	201	195

Catalytic Performance in BDH

Minor products competing with the formation of isobutene are *cis*- and *trans*-2-butenes and 1-butene. Ga1-SiO_{2-500} shows an initial selectivity to *cis*- and *trans*-2-butenes and 1-butene of 12.6%, followed by Ga5-SiO_{2-500} (9.1%) and Ga10-SiO_{2-500} (5.2%). This selectivity trend correlates with the relative amount of strong BAS in Ga1-, Ga5- and Ga10-SiO_{2-500} catalysts. We also note that isomerization of 1-butene and 2-butenes to isobutene typically proceeds on strong BAS at 500 °C (Figure 6b).[4-6] The selectivity to isobutene isomers decreased on all three catalysts after 20 h TOS. In addition to C_4 olefins, propene and methane are formed in a 1:1 molar ratio for all catalysts and the formation rates of methane and propene on Ga1-, Ga5- and Ga10-SiO_{2-500} are comparable of that of pure SiC (diluent of the catalytic tests), indicating that propene and methane are thermal cracking products (Figure S37). Compared to isobutene and its isomers, the formation rate of methane and propene was more stable, which explains their increased selectivity after 20 h TOS (Figure 6b).

Aromatic products, including benzene, toluene, xylenes and ethylbenzene are also detected on all three catalysts (Figure S38). Interestingly, Ga5-SiO_{2-500} exhibits the highest STY of aromatics (formed in µmol amounts per h^{-1} g_{cat}^{-1}) compared to Ga1-SiO_{2-500} and Ga10-SiO_{2-500}, suggesting that a balance of both strong BAS and mild/strong LAS may play a role in the aromatization reaction. Among all aromatic products, the partial selectivity to C_8 aromatics exceeds 70%, explained by the aromatization of butene dimers. The STY of aromatics decreases with TOS on all three catalysts, but to a lower extent on Ga1-SiO_{2-500} compared to Ga5- and Ga10-SiO_{2-500}.

Reaction-regeneration cycles were carried out by passing synthetic air at 500 °C through the catalyst bed for 1 h after 5 h TOS (Figure S39). All three catalysts can be fully regenerated, specifically, there are no significant differences in STY and selectivity between the first and the third cycles for Ga1-SiO_{2-500}, whereas an increase in the initial catalytic activity was observed for Ga5- and Ga10-SiO_{2-500} after the first regeneration cycle. This increased activity is maintained in every subsequent regeneration cycle (Figure S39). Interestingly, the formation of toluene and C_8 aromatics exhibit a similar activation behavior with TOS as the formation rate to isobutene, while the formation rate to benzene shows a different trend. The underlying reason for these effects is currently unclear. In addition, while the carbon balance for all three catalysts, after the first 30 min of TOS, remained stable at ca. 96% (Figure S39), the color of spent Ga5- and Ga10-SiO_{2-500} catalysts changed from light grey to black after 20 h TOS, while the color of spent Ga1-SiO_{2-500} remained light grey. This observation can be explained as follows: the presence of more abundant mild LAS in Ga5- and Ga10-SiO_{2-500} contributes to a higher degree of coking on these catalysts. This observation is also consistent with the higher stability of Ga1-SiO_{2-500} in BDH owing to a lower degree of coking as compared to Ga5- and Ga10-SiO_{2-500}.

To better distinguish the three ALD-based Ga-SiO_{2-500} catalysts in terms of their catalytic performances, we further increased the BDH reaction temperature from 500 °C to 550 °C. Since *in situ* XRD shows no structural evolution of Ga10-SiO_{2-500} at least up to ca. 800 °C, no structural changes are expected to occur at 550 °C relative to 500 °C. While an increased isobutane conversion is observed on all three Ga-SiO_{2-500} catalysts at the higher reaction temperature, the selectivity to isobutene decreased significantly, owing to an enhancement of the cracking reactions (Table S7, Figure S40-S42). The initial selectivity to isobutene is 64%, 68% and 75% for Ga1-, Ga5- and Ga10-SiO_{2-500}, respectively. This selectivity trend correlates well with the relative abundance of strong BAS in the tested catalysts. Comparing the isobutene formation rate normalized by the Ga loading and surface area shows that the activity of Ga1-SiO_{2-500} (89.2 mmol h^{-1} mol_{Ga}^{-1} m^{-2}) is higher than that of Ga5-SiO_{2-500} (38.4 mmol h^{-1} mol_{Ga}^{-1} m^{-2}) and Ga10-SiO_{2-500} (28.6 mmol h^{-1} mol_{Ga}^{-1} m^{-2}). The stable formation of methane and propene leads to a decreased selectivity to butenes during the catalytic test (Figure S41). It is worth

noting that the initial selectivity to isomers of isobutene (i.e., *cis*-2-butene, *trans*-2-butene and 1-butenes) on Ga1-, Ga5-, and Ga10-$SiO_{2\text{-}500}$ is 12.5%, 10.1% and 6.1%, respectively. After 20 h TOS, the selectivity to isobutene isomers becomes negligible for Ga5- and Ga10-$SiO_{2\text{-}500}$, but stays at 8.5% on Ga1-$SiO_{2\text{-}500}$, suggesting that coke deposition blocks more isomerization sites on Ga5- and Ga10-$SiO_{2\text{-}500}$ relative to Ga1-$SiO_{2\text{-}500}$ (Figure S42). The lower deactivation constant (k_d) obtained for Ga1-$SiO_{2\text{-}500}$ (0.051 s^{-1}) in comparison to Ga5-$SiO_{2\text{-}500}$ (0.073 s^{-1}) and Ga10-$SiO_{2\text{-}500}$ (0.078 s^{-1}) is in agreement with results obtained at 500 °C (see the main text), where a higher fraction of mild LAS, ascribed to pentacoordinate Ga sites, correlated with coking.

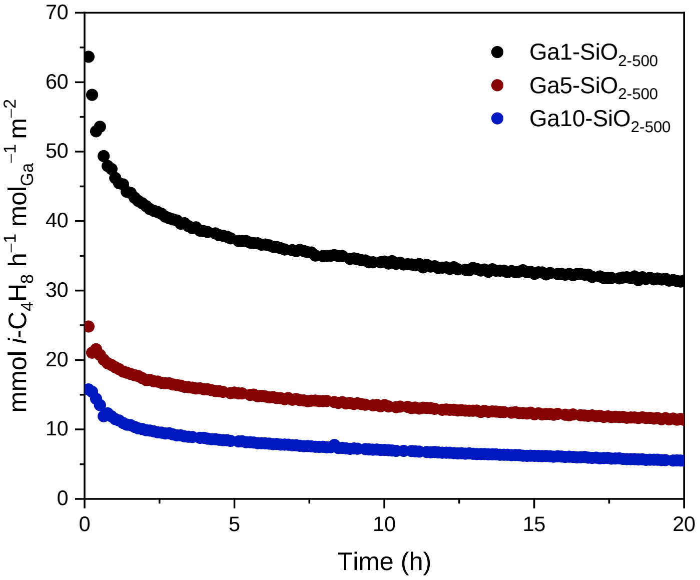

Figure S33. Isobutene formation rates normalized by the respective Ga loadings and surface areas (mmol i-C_4H_8 h^{-1} g_{Ga}^{-1} m^{-2}) over 20 h TOS on Ga1-, Ga5- and Ga10-$SiO_{2\text{-}500}$.

Reaction conditions: 10% of i-C_4H_{10} in N_2, WHSV = 8.5 h^{-1}, T = 500 °C.

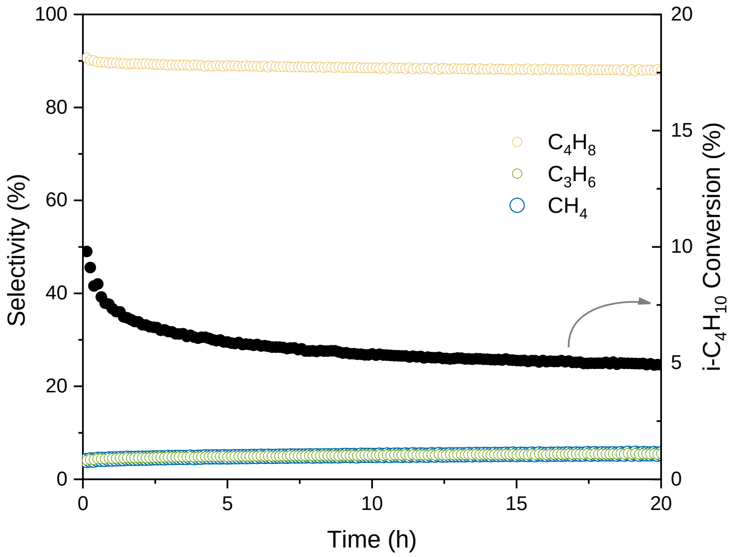

Figure S34. Isobutane conversion and product selectivity to C_4H_8 (yellow), C_3H_6 (green) and alkanes (blue) on Ga1-$SiO_{2\text{-}500}$ over 20 h TOS.

Reaction conditions: 10% of i-C_4H_{10} in N_2, WHSV = 8.5 h^{-1}, T = 500 °C.

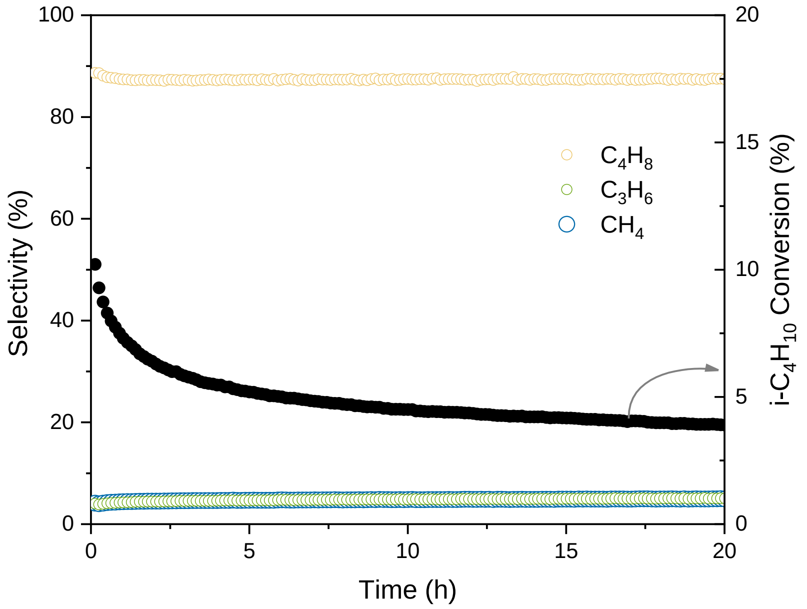

Figure S35. Isobutane conversion and product selectivity of C_4H_8 (yellow), C_3H_6 (green) and alkanes (blue) on Ga5-SiO_{2-500} over 20 h TOS.

Reaction conditions: 10% of i-C_4H_{10} in N_2, WHSV = 8.5 h^{-1}, T = 500 °C.

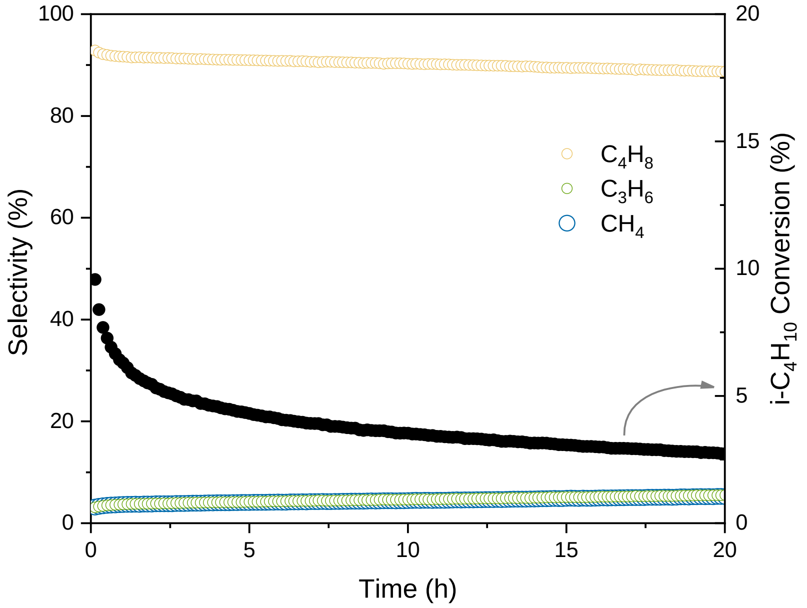

Figure S36. Isobutane conversion and product selectivity of C_4H_8 (yellow), C_3H_6 (green) and alkanes (blue) on Ga10-SiO_{2-500} over 20 h TOS.

Reaction conditions: 10% of i-C_4H_{10} in N_2, WHSV = 8.5 h^{-1}, T = 500 °C.

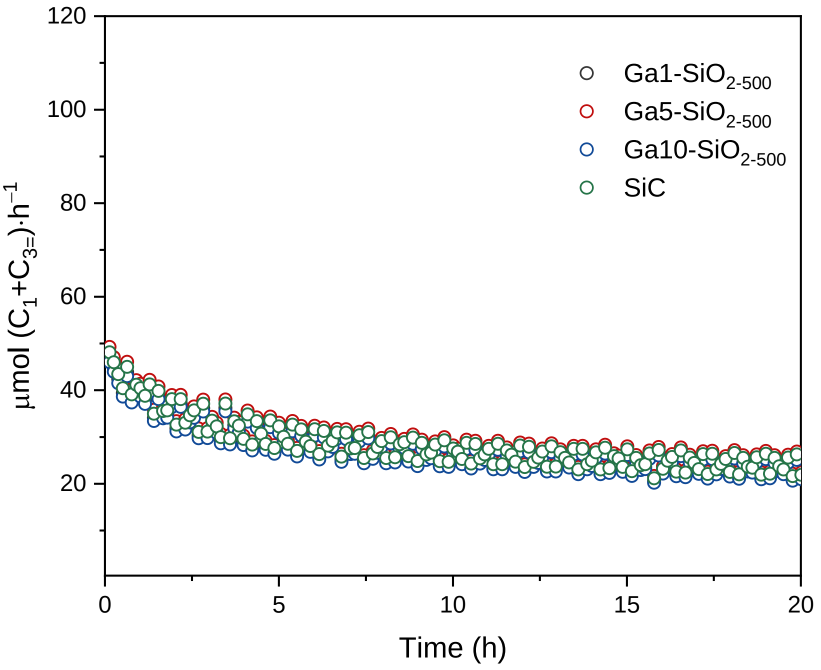

Figure S37. Formation rates to cracking products (combined rates to methane and propene, µmol h^{-1}) on Ga1-, Ga5-, Ga10-SiO_{2-500} and SiC.

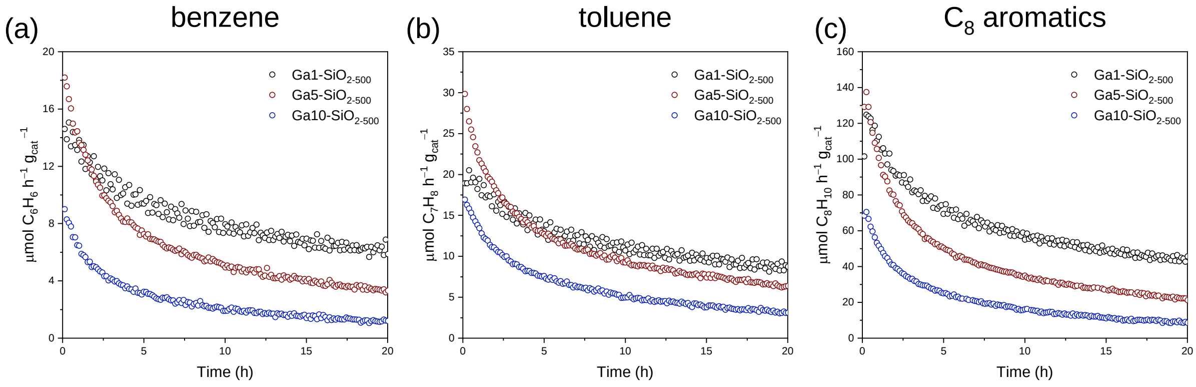

Figure S38. Space-time-yield (µmol h^{-1} $g_{cat}{}^{-1}$) of (a) benzene, (b) toluene and (c) xylenes over 20 h TOS for Ga1-, Ga5- and Ga10-SiO_{2-500}.

Reaction conditions: 10% of *i*-C_4H_{10} in N_2, WHSV = 8.5 h^{-1}, T = 500 °C.

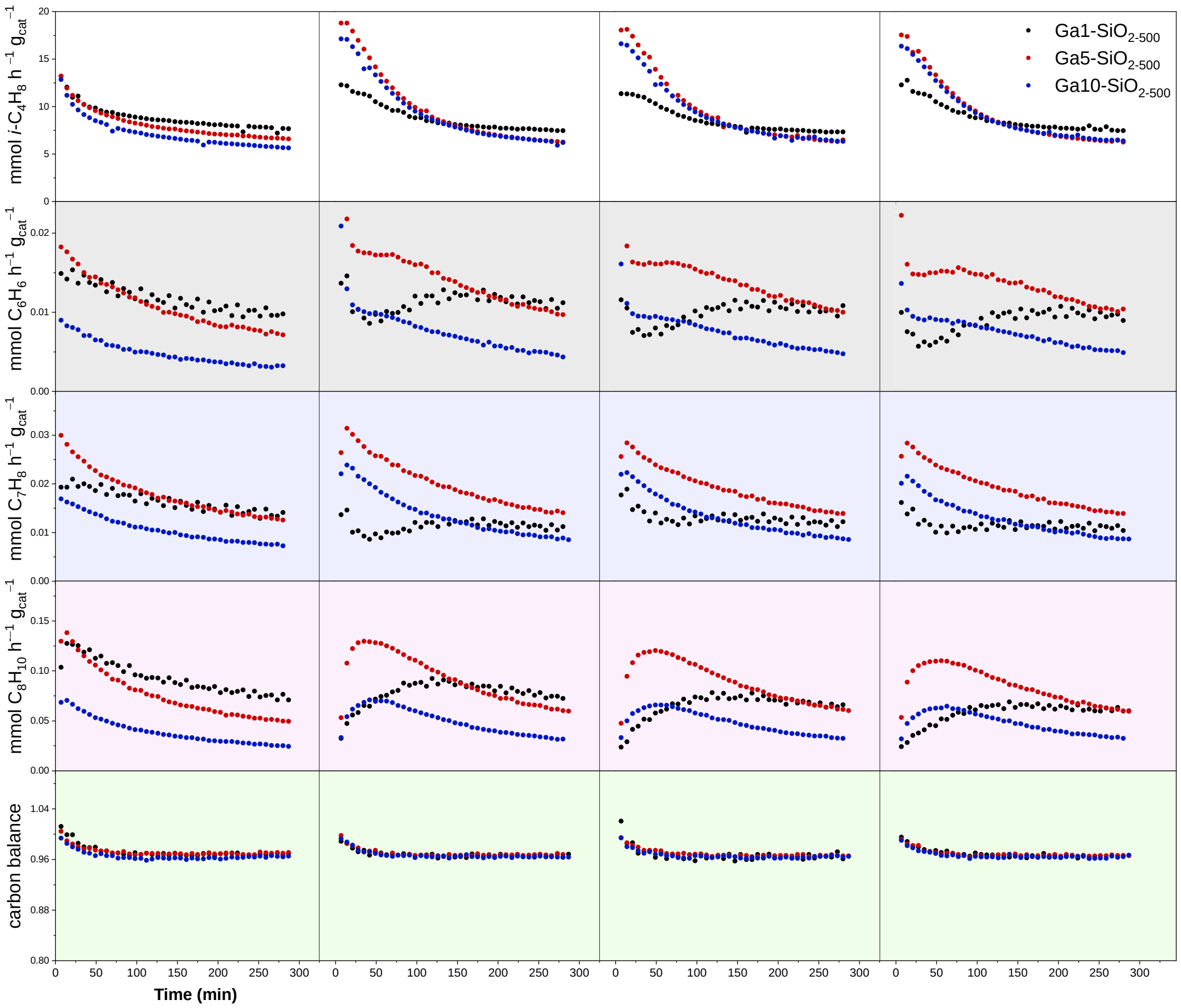

Figure S39. Space-time-yield (mmol h^{-1} g_{cat}^{-1}) of isobutene, benzene, toluene and C_8 aromatics (ethylbenzene and *m*-, *p*-, *o*-xylene) and carbon balance on Ga1-(black), Ga5-(red), Ga10-$SiO_{2\text{–}500}$ (blue) over 20 h (4 × 5 h) TOS including three regeneration cycles after every 5 h.

Table S7. Results from BET, ICP and catalytic tests. Catalytic data is presented after 7 min TOS and, in parentheses, after 20 h TOS. Reaction condition: 10% of *i*-C_4H_{10} in N_2, WHSV = 8.5 h^{-1}, T = 550 °C.

Material	S_{BET} (m^2 g^{-1})	Ga content (wt%)	Isobutane conversion (%)	Isobutene selectivity (%)	k_d (s^{-1})	Rate of isobutene production	
						mmol h^{-1} g_{cat}^{-1}	mmol h^{-1} mol_{Ga}^{-1} m^{-2}
Ga1-$SiO_{2\text{–}500}$	280	4.9	16.5 (6.6)	64 (53)	0.051	17.5 (6.4)	89.2 (32.3)
Ga5-$SiO_{2\text{–}500}$	200	18.2	18.1 (4.9)	68 (52)	0.073	16.1 (4.8)	38.4 (9.1)
Ga10-$SiO_{2\text{–}500}$	160	31.9	17.3 (4.2)	75 (49)	0.078	16.9 (3.9)	28.6 (5.5)

k_d=[ln(1 − $conv_{end}$/$conv_{end}$) − ln(1 − $conv_{start}$/$conv_{start}$)]/t.[7]

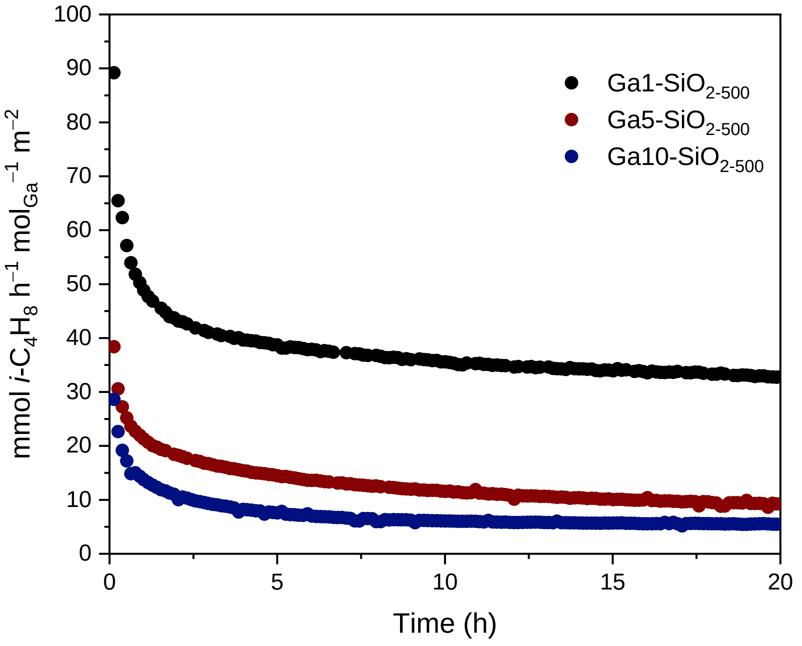

Figure S40. Isobutene formation rates normalized by the respective Ga loadings and surface areas (mmol i-C_4H_8 h^{-1} g_{Ga}^{-1} m^{-2}) over 20 h TOS on Ga1-, Ga5- and Ga10-$SiO_{2\text{-}500}$.

Reaction conditions: 10% of i-C_4H_{10} in N_2, WHSV = 8.5 h^{-1}, T = 550 °C.

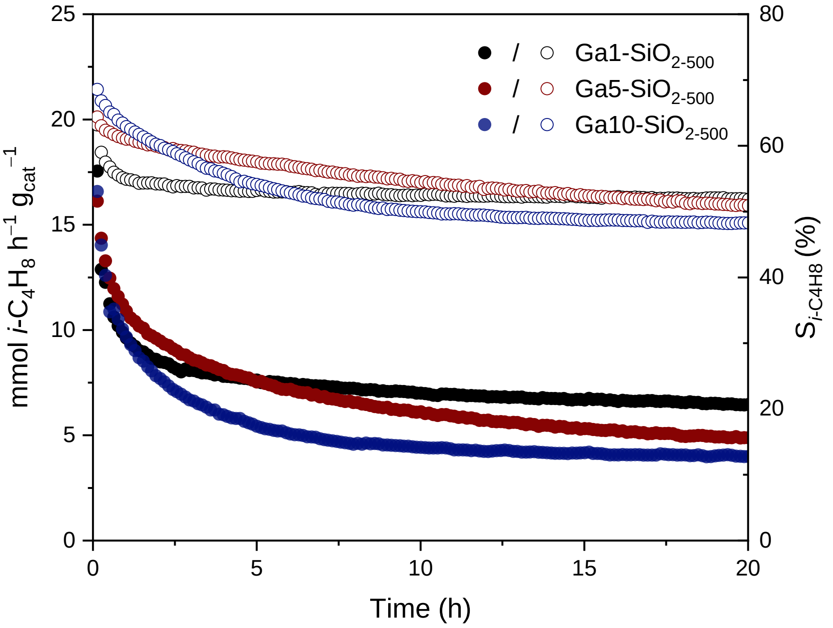

Figure S41. Space-time-yield (mmol i-C_4H_8 h^{-1} g_{cat}^{-1}) and selectivity of isobutene over 20 h TOS on Ga1-, Ga5- and Ga10-$SiO_{2\text{-}500}$.

Reaction condition: 10% of i-C_4H_{10} in N_2, WHSV = 8.5 h^{-1}, T = 550 °C.

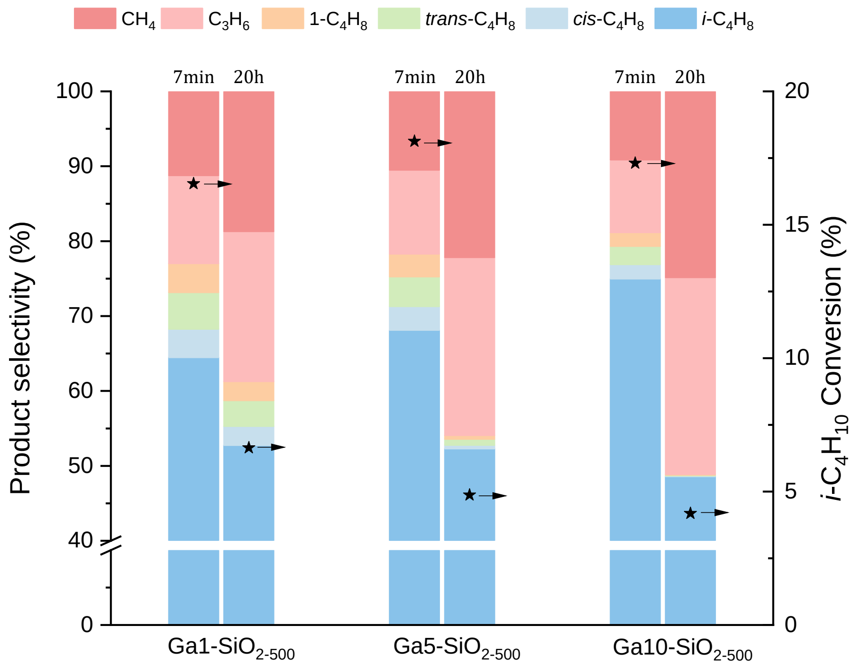

Figure S42. Initial product selectivity and *i*-C_4H_{10} conversion on Ga1-, Ga5-, and Ga10-$SiO_{2\text{–}500}$.

Reaction conditions: 10% of *i*-C_4H_{10} in N_2, WHSV = 8.5 h^{-1}, T = 550 °C.

Table S8. Results from BET, ICP and catalytic tests. Catalytic data is presented after 7 min TOS and, in parentheses, after 5h TOS. Reaction condition: 10% of *i*-C_4H_{10} in N_2, WHSV = 8.5 h^{-1}, T = 500 °C.

Material	S_{BET} (m^2 g^{-1})	Al content (wt%)	Isobutane conversion (%)	Isobutene selectivity (%)
Al1-$SiO_{2\text{–}500}$	287	3.4	0.6 (0.3)	51 (54)
Al5-$SiO_{2\text{–}500}$	167	9.2	0.5 (0.4)	53 (54)
Al10-$SiO_{2\text{–}500}$	171	15.4	0.8 (0.6)	51 (52)
SiC	–	–	0.5 (0.3)	41 (43)

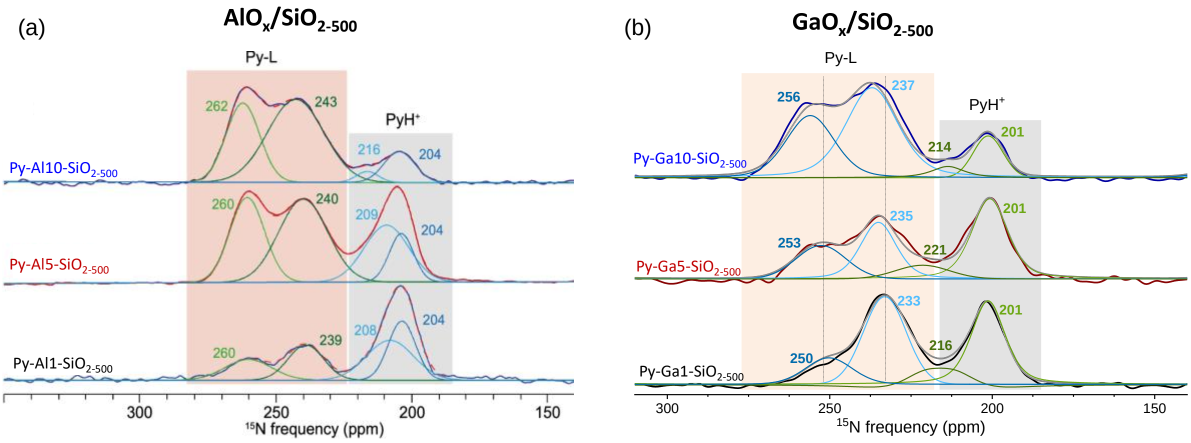

Figure S43. Comparison between ^{15}N DNP SENS spectra of Py-Al1,5,10-SiO_{2-500} (a) and Py-Ga1,5,10-SiO_{2-500} (b) after desorption of pyridine at 150 °C. Note that the ^{15}N DNP SENS spectra of Py-Al1,5,10-SiO_{2-500} have been reported by us previously and are reproduced here for comparison.[8] Copyright 2021, the American Chemical Society (ACS).

References

(1) Comstock, D. J.; Elam, J. W., Atomic Layer Deposition of Ga_2O_3 Films Using Trimethylgallium and Ozone. *Chem. Mater.* **2012,** *24*, 4011-4018.

(2) Castro-Fernández, P.; Blanco, M. V.; Verel, R.; Willinger, E.; Fedorov, A.; Abdala, P. M.; Müller, C. R., Atomic-Scale Insight into the Structure of Metastable γ-Ga_2O_3 Nanocrystals and their Thermally-Driven Transformation to β-Ga_2O_3. *J. Phys. Chem. C.* **2020,** *124*, 20578-20588.

(3) Granlund, L.; Billinge, S. J.; Duxbury, P. M., Algorithm for Systematic Peak Extraction from Atomic Pair Distribution Functions. *Acta Crystallogr. A: Found. Adv.* **2015,** *71*, 392-409.

(4) Matveyeva, A. N.; Omarov, S. O.; Sladkovskiy, D. A.; Murzin, D. Y., Experimental Studies and Kinetic Regularities of Isobutane Dehydrogenation over Ga_2O_3/Al_2O_3. *J. Chem. Eng* **2019,** *372*, 1194-1204.

(5) Otroshchenko, T.; Jiang, G.; Kondratenko, V. A.; Rodemerck, U.; Kondratenko, E. V., Current Status and Perspectives in Oxidative, Non-oxidative and CO_2-mediated Dehydrogenation of Propane and Isobutane over Metal Oxide Catalysts. *Chem. Soc. Rev.* **2021,** *50*, 473-527.

(6) Wang, G.; Li, C.; Shan, H., Catalytic Dehydrogenation of Isobutane over a Ga_2O_3/ZnO Interface: Reaction Routes and Mechanism. *Catal. Sci. Technol.* **2016,** *6*, 3128-3136.

(7) Searles, K.; Chan, K. W.; Mendes Burak, J. A.; Zemlyanov, D.; Safonova, O.; Coperet, C., Highly Productive Propane Dehydrogenation Catalyst Using Silica-Supported Ga-Pt Nanoparticles Generated from Single-Sites. *J. Am. Chem. Soc.* **2018,** *140*, 11674-11679.

(8) Kaushik, M.; Leroy, C.; Chen, Z.; Gajan, D.; Willinger, E.; Müller, C. R.; Fayon, F.; Massiot, D.; Fedorov, A.; Copéret, C.; Lesage, A.; Florian, P., Atomic-Scale Structure and Its Impact on Chemical Properties of Aluminum Oxide Layers Prepared by Atomic Layer Deposition on Silica. *Chem. Mater.* **2021,** *33*, 3335-3348.
